# The impact of expressive language development and the left inferior longitudinal fasciculus on listening and reading comprehension

**DOI:** 10.1186/s11689-019-9296-7

**Published:** 2019-12-16

**Authors:** Stephanie N. Del Tufo, F. Sayako Earle, Laurie E. Cutting

**Affiliations:** 1 Peabody College of Education and Human Development, Vanderbilt University, 416C One Magnolia Circle, Box 228, Nashville, TN 37203 USA; 2 Vanderbilt Brain Institute, Vanderbilt University School of Medicine, 6133 Medical Research Building III, 465 21st Avenue South, Nashville, TN 37232 USA; 3 Vanderbilt Kennedy Center, Vanderbilt University, 110 Magnolia Circle, Nashville, TN 37203 USA; 4 College of Education and Human Development, University of Delaware, 106 Alison Hall West, Newark, DE 19716 USA; 5 Communication Sciences and Disorders, University of Delaware, 100 Discovery Boulevard, Newark, DE 19713 USA

**Keywords:** Expressive language development, Passage comprehension, Inferior longitudinal fasciculus, Genre, Modality, Intervention, Developmental disorder, Socioeconomic, Longitudinal, Survival analysis

## Abstract

**Background:**

During the first 3-years of life, as the brain undergoes dramatic growth, children begin to develop speech and language. Hallmarks of this progression are seen when children reach developmental milestones, forming the foundation of language. Expressive language milestones, such as the production of a child’s first word, are delayed in 5–8% of children. While for some children delays in reaching these milestones are harbingers of developmental disorders, for others expressive language delays appear to resolve. Regardless of whether or not early language skills appear resolved, difficulty with later comprehension is a likely outcome. Whether this heightened risk for poor comprehension differs based on text features, individual characteristics, or receipt of intervention remains unknown. Moreover, this relationship between expressive language development and comprehension is not yet linked to neurobiology, though the inferior longitudinal fasciculus (ILF) is a potential neurobiological correlate. Therefore, we investigated the impact of, and interactions between, expressive language development, early intervention, and the ILF on comprehension.

**Methods:**

Longitudinal recurrent survival analyses predicted the risk of answering a comprehension question incorrectly. Predictors of comprehension included expressive language development, passage features, participant characteristics, fractional anisotropy, receipt of early intervention, and later diagnosis of speech or language disorders.

**Results:**

Children with later expressive language milestones had poorer comprehension. When comprehension text features were examined, children with later milestones had poorer listening and reading comprehension, and poorer narrative and expository comprehension. The left ILF acted as a neurodevelopmental correlate, one that moderated the relationship between expressive language milestones and comprehension. Specifically, the left ILF exacerbated the relationship for those who did not receive early intervention and buffered the relationship for those who received intervention services. Early intervention decreased the risk of poor comprehension by 39% for children later diagnosed with a speech or language disorder.

**Conclusions:**

Early intervention should be provided for children with delayed expressive language milestones, particularly those who are at risk for speech or language disorders. The ILF plays a critical role in the relationship between expressive language development and comprehension, which may be that of a protective factor for children with the most severe early issues with speech and language.

## Background

One of the most common reasons that children are evaluated for early intervention is due to an observed delay in, or a failure to reach, expressive language milestones [67, 126]. Early problems in expressive language may reflect developmental delays in some, while in others missed expressive language milestones are indicative of later emergence of developmental disorders. It is estimated that 5–8% of children experience difficulties in expressive language development [66, 111]. Despite this prevalence of early problems with expressive language, we know relatively little about the neurobiological development of children with such difficulties.

### Early problems in expressive language are associated with later issues with comprehension

Behaviorally, early problems with expressive language development have been linked to a heightened risk for later difficulties in spoken and written language, as well as academic achievement [5, 6, 63, 113, 114]. This heightened risk appears to be tied specifically to global language skills, rather than the ability to perform discrete linguistic tasks. To illustrate, those with a history of early expressive language problems, when tested during school-age, perform within normal limits on vocabulary, grammar, and word-level reading or decoding [76, 88, 96–98]. Despite this, the overall language profiles of such individuals, throughout childhood and adolescence, generally appear to be weak [96–98]. Broad (sub-clinical) language weakness suggests that communication failure is more likely to occur when linguistic demands are high.

Language comprehension is a linguistic skill that places a high demand on one’s linguistic (as well as executive functioning) capacity [13, 17, 18, 27, 83, 84];). There is some evidence to suggest that early problems with expressive language predict later issues with both oral and reading comprehension. For example, Zielinski et al. [137] found that preschoolers with a history of expressive language delay were more likely to have difficulties with reading comprehension, and lower performance overall. Poll and Miller [92] found that delayed expressive language was a significant risk factor for poor oral language and reading comprehension at 8 years old. Similarly, Lee [70] reported that expressive language development predicted passage comprehension in both 3rd and 5th grade, while Bleses et al. [14] found that expressive language development predicted reading comprehension as late as 6th grade. Additionally, Duff et al. [34] found that infant vocabulary between 16 and 24 months (a latent variable comprised of both expressive and receptive measures) predicted reading comprehension, roughly 5 years later. Psyridou et al. [94] found that expressive language at age 2–2.5 years old was associated with reading comprehension in grades 2, 3, 8, and 9 (ages 8–16 years old). While the evidence is stronger for reading comprehension than oral (listening) comprehension, taken together it suggests that early expressive language skills prior to age 3 are associated with later comprehension performance.

The emergence of comprehension difficulties may differ according to comprehension modality. Listening and reading comprehension are highly interrelated [116], as listening comprehension is theorized to play a key role in the development of reading comprehension (e.g., [56]). A number of studies have found that while listening comprehension exceeds reading comprehension performance during pre-K-to-1st grade, the relationship between listening and reading comprehension becomes significantly stronger following second grade [25, 30, 116]. Moreover, the demands on linguistic knowledge for reading increase dramatically between 3rd and 4th grade, due to the instructional focus shifting from learning-to-decode to extracting the learning objectives from the written text (e.g., [118]). Thus, reading comprehension difficulties may be more pronounced than observed difficulties with listening comprehension during later elementary school.

Linguistic demand can also vary across the text category. The category of text, or genre, can be narrative, expository, persuasive, or descriptive [12, 89]. Narrative text is considered easier to read, comprehend, and recall information from, than expository text [47, 48]. This remains true, even after topic familiarity and vocabulary are controlled [47]. Narrative passages typically include characters, goals, settings, and a consistent rhetorical structure [115, 121], while expository passages instead contain factual information and have multiple rhetorical forms [53]. Much of the previous work into comprehension performance has focused on narrative comprehension [15, 36, 75, 139]. However, there are a few studies that have directly compared comprehension across narrative and expository texts. For example, Diakidoy et al. [30] found that expository comprehension level is lower than narrative comprehension level. As such, poorer listening and reading comprehension performance is likely to be greater for expository than narrative text.

In addition to a history of expressive language difficulties, the likelihood of comprehension failure may be influenced by the presence and/or timing of intervention services. For example, targeted instruction has been observed to contribute to comprehension growth in general, but the effect of instruction is more pronounced for younger children [1]. And while the evidence suggests that there are interventions that are effective for comprehension (e.g., [22]), not all children with delays in early expressive language receive intervention services. This may be because some children appear to catch up to their peers [33, 39]; however, as discussed, many children with early problems in expressive language perform within normal limits on tests of vocabulary, grammar, etc., while maintaining a weak language profile overall [96–98]. As such, understanding the neurobiological underpinnings of comprehension issues, specifically those that follow later onsets of reaching expressive language development milestones, may help parents and clinicians to better assess whether a particular child would benefit from intervention.

As mentioned, despite the prevalence of early problems with expressive language, we know relatively little about the neurobiological development of children with such difficulties. Importantly, this may explain the difficulties in language comprehension that are predicted to emerge later on. To our knowledge, the heightened risk for comprehension failure in this population is not linked to any known neurobiological mechanism. Identifying such a mechanism could provide a means by which to examine the impact of intervention services on the neurodevelopment of structures relevant to language comprehension. Thus, in order to identify a structure of interest as a potential biomarker of comprehension ability, we now turn to the available neuroimaging literature on listening and reading comprehension.

### The neurobiological basis of comprehension

The neurobiological basis of listening and reading comprehension has been studied fairly extensively in adults. A meta-analysis that synthesized the functional magnetic resonance imaging (fMRI) findings on adult reading comprehension indicated the robust involvement of the anterior temporal lobes and the fronto-medial cortex across tasks [38], with the engagement of subcortical structures (e.g., posterior cingulate cortex) modulated by the narrative coherence of stimuli. There are far fewer neuroimaging studies on listening or reading comprehension in school-age children. One such study by Cutting et al. [26] investigated differences in neural activity during word-level reading between school-age children with good reading skills, developmental dyslexia, and specific reading comprehension deficits (S-RCD). Unlike children with developmental dyslexia, children with S-RCD demonstrated neural activation patterns that were similar to children with typical reading ability in general. However, these patterns became atypical when the demands of lexical access were increased, suggesting that children with S-RCD have weaknesses in accessing lexical-semantic representations. Another fMRI study investigated neural activation in children during a narrative comprehension task [57]. The authors found that greater activation of frontal regions and the supramarginal gyrus at ages 5–7 predicted better reading comprehension at age 11. Moreover, at 11 years old, stronger temporal and occipital activation during the same task correlated with better reading comprehension. Taken together, the recruitment of temporal-occipital regions, likely relating to access to lexical-semantic representations, appears to potentially be important to language comprehension during childhood.

While few functional neuroimaging studies have examined reading or listening comprehension in school-aged children, fewer still have tied differences in comprehension ability directly to differences in neurobiological structure. In adults, Saur et al. [108] found that spoken language comprehension is supported by the white matter ventral pathway, which connects the middle temporal lobe and the ventrolateral prefrontal cortex via the extreme capsule, middle longitudinal fasciculus, and the inferior longitudinal fasciculus (ILF). Furthermore, fractional anisotropy of the ILF has been shown to positively correlate with reading comprehension [58, 85], and intensive reading training has been found to cause rapid changes in the left ILF [60]. Thus, we have reasons to suspect that the development of the ILF during childhood may relate to comprehension behavior.

The literature on the behaviors thought to be sub-served by the ILF is emerging. The ILF is a ventral association bundle that connects the occipital lobe with the anterior portion of the temporal lobe with long and short fibers, which lies inferiorly parallel to the lateral wall of the temporal horn. The left ILF appears to primarily play a role in lexical/semantic processes [35, 52, 54, 77, 103, 104, 108, 122], reading [37, 58, 136], and sound-to-word learning [131]. Taken together with the functional neuroimaging work discussed above, the left ILF is likely particularly important for spoken and written language comprehension. As such, the early structure of the ILF may be a potential biomarker for the likelihood of poorer comprehension performance in school-age children.

### Research questions

An overarching aim of this paper is to determine to what extent the developmental timing of expressive language and the fractional anisotropy (FA) of the left ILF alter the risk of poor comprehension in primary school-aged children. To this aim, we first examined the onset of expressive language developmental markers (e.g., age of babbling onset) as a potential predictor of poorer comprehension later in development. We also asked if comprehension was influenced by text features, such as modality (listening vs. reading) and genre (expository vs. narrative), or participant features, such as sex, socioeconomic status, birth (premature vs. full-term), and an early history of frequent ear infections with or without pressure equalizer (PE) tubes placed (part 1). We hypothesized that poorer listening and reading comprehension would be more likely in individuals with greater delays in expressive language development while listening to expository text.

Second, we investigated if the FA of the left ILF would act as a potential neurodevelopmental correlate of listening and reading comprehension, as well as if the left ILF (FA) moderated the effect of expressive language development on comprehension (part 2). We hypothesized that poorer listening and reading comprehension would be more likely in individuals with less FA in their left ILF. Moreover, we hypothesized that those individuals with the highest levels of left ILF FA would manifest a stronger relationship between the onset of expressive language development and poorer comprehension.

Finally, previous research has found that without early intervention, children with delays in expressive language development may or may not catch up to their peers (e.g., [39, 109]). We sought to determine if intervention prior to age 3 would alter the likelihood of poorer comprehension (part 3), as well as if early intervention would decrease the likelihood of poorer comprehension in those later reported to have a speech and/or language disorder (part 4). We hypothesized that those with a speech or language issue who received early intervention would be less likely to have poor comprehension. Below, we present the methods and findings that confirm these hypotheses and highlight the role of the left ILF as an important biomarker of listening and reading comprehension.

## Methods

### Participants

Data included in this manuscript is from two separate longitudinal studies (see below: Cohorts 1 and 2). Both studies were conducted in accordance with approval from the Vanderbilt University Institutional Review Board. A parent or guardian provided informed consent for all participating children. Participating children provided informed assent and received compensation for their participation in studies. A total of 1297 (601 male; 696 female) 1st graders completed prescreening. Prescreening took place either in the classroom or at Vanderbilt’s Education and Brain Sciences Research Laboratory (https://vkc.mc.vanderbilt.edu/ebrl/). When prescreening took place in the classroom, teachers received compensation for their completion of prescreening questionnaires. Of those children who completed prescreening, a total of 340 (159 male; 181 female) children enrolled in the longitudinal studies. The longitudinal studies follow children annually from the end of 1st grade to the end of 4th grade. Longitudinal neuroimaging study inclusion criteria required that children were MRI compatible, native speakers of American English with no known history of neurological problems, had normal hearing, and normal or corrected-to-normal vision.

#### Cohort 1

Prescreening data was collected in the 1st-grade classrooms of greater Nashville, Tennessee area schools with permission of school principals and teachers. During prescreening, children completed 1 hour of in-school standardized assessments. Of the 866 children enrolled, 821 children (377 male; 444 female) completed pre-screening. The remaining 45 children either withdrew from the study or their families moved prior to prescreening taking place. Of the 821 children who completed prescreening, 15 were Asian (1.9%), 206 were Black/African American (25.6%), 510 were Caucasian (63.3%), 26 were Hispanic (3.2%), 1 was Kurdish (0.1%), 37 were more than one race (4.6%), and 26 were not reported (3.2%).

Longitudinal data was collected in two waves at Vanderbilt’s Education and Brain Sciences Research Laboratory and the Vanderbilt Institute of Imaging Science (https://vuiis.vumc.org). Data collection began following children’s completion of 1st grade. During the longitudinal study, children completed standardized and experimental assessments and neuroimaging annually for 4 years. Additionally, the children and their parents completed language and reading questionnaires. From the initial sample, 140 (65 male; 75 female) children chose to participate in longitudinal data collection and met inclusionary criteria. Of the 140 children who participated in the longitudinal study, 1 was Asian (0.7%), 36 were Black/African American (27.3%), 85 were Caucasian (60.7%), 1 was Native American (0.7%), 14 were more than one race (10.1%), and 1 was not reported (0.7%). Additionally, 8 participants were Hispanic/Latino (5.8%), 129 were not Hispanic/Latino (93.5%), and 3 were not reported (2.1%).

#### Cohort 2

Prescreening data was collected in two locations: the 1st-grade classrooms of schools in the greater Nashville area with permission of school principals and teachers, and at Vanderbilt’s Education and Brain Sciences Research Laboratory. Of the 1001 children whose parents consented to prescreening data being collected, prescreening was complete by 476 (224 male; 252 female) children. Of those who completed prescreening, 19 were Asian (4.0%), 75 were Black/African American (15.9%), 361 were Caucasian (76.3%), 1 was Native Hawaiian (0.2%), 1 was Pacific Islander (0.2%), 33 were more than one race (and may or may not have also identified those races) (7.0%), and 5 were not reported (1.0%). Additionally, 14 participants were Hispanic/Latino (2.9%), 454 were not Hispanic/Latino (95.6%), and 8 were not reported (1.6%).

Longitudinal data collection began following children’s completion of 1st grade and was collected as in Cohort 1. From the initial sample, 200 (94 male; 106 female) children chose to participate in longitudinal data collection and met inclusionary criteria. Of the 200 children who participated in the longitudinal study, 9 were Asian (4.5%), 25 were Black/African American (12.6%), 159 were Caucasian (79.9%), 15 were more than one race (and may or may not have also identified those races) (7.5%), and 4 were not reported (2%). Additionally, ten participants were Hispanic/Latino (5.0%), 184 were not Hispanic/Latino (92.0%), and six were not reported (3.0%).

### Data

Data in the current study includes demographics, information from questionnaires, standardized and experimental assessments, as well as neuroimaging data. Children’s demographic information was provided by both teachers and parents. Questionnaire items selected for inclusion focused on the development of, and early developmental issues with, speech and reading. Additional, control questions selected for inclusion were those previously linked with delays in early expressive language. Responses for some of the questionnaire items that were not expected to change over time (e.g., Age at which your child learned to read?) were collected annually. These questions were asked annually to determine parents’ internal response reliability. Experimental and standardized measures of reading and listening comprehension are included. Magnetic resonance imaging (MRI) diffusion and structural gray matter scans are included in the current study.

### Procedure

Across a number of parent questionnaires, items regarding speech/reading development, as well as issues with speech/reading, were selected for the current manuscript. We focused on questions related to the early development of expressive language (see Questionnaires below). From the standardized and experimental assessment measures, we identified the listening or reading comprehension measure that was administered the most frequently at all-time points for recurrent event survival analysis (see Assessments below). In Cohort 1, the Qualitative Reading Inventory (QRI: [72]) measures (administered at four time-points) included both listening and reading comprehension. In Cohort 2, we used an in-house version of passage comprehension that mimicked the QRI, including both listening and reading comprehension, but controlled for additional factors (e.g., number of passage words, number of comprehension questions). We then compared the QRI and our in-house measure of passage comprehension to the less frequently administered measures of reading and listening comprehension, by time point administered (see Additional file 1 Appendix 1 Supplemental Information). Finally, fractional anisotropy (FA) of the left inferior longitudinal fasciculus (ILF) was included by subject and time point (see Diffusion imaging).

### Questionnaires

Questionnaire items, regarding issues with speech and/or reading prior to beginning 1st grade, were selected from among those administered to parents. The Child History Questionnaire is a parent questionnaire developed to assess a child’s history across a broad range of areas [7]. Here, we focused on the early development portion of the Child History Questionnaire. In this section, parents were asked to “Please indicate the age at which your child first demonstrated each behavior.” We focused on three of the infant and preschool behaviors, which related to language: (1) babbled, (2) spoke first word, and (3) put several words together. Parental responses for all three questions were ranges from 0–6 months, 7–12 months, 12–24 months, 2–3 years, 4–5 years, and 5+ years. This data was then coded as an ordinal covariate taking values of 0 (0–6 months), 1, 2, 3, 4, or 5 (5+ years), respectively. Data collected on these questions was only requested from parents at time 1, and as such is considered time-invariant for statistical analysis purposes. Children who are the latest to develop expressive language, are classified as having Late Language Emergence when they fail to combine two or more words by 24 months [92, 93, 102]. Prevalence estimates of these children suggest that between 10 and 20% of our sample will meet this criterion for Late Language Emergence [95, 101, 138].

Parents were asked to complete an in-house Reading Questionnaire [26] and asked to report if their child had a diagnosed speech or language disorder. Parents were also asked if prior to age 3 their child received an early intervention for speech or language difficulty. Question responses were coded as yes (coded 1), no (coded 0), and any “I don’t know” responses were removed. Data collected on these questions was only requested from parents at time 1 and as such is considered time-invariant for statistical analysis purposes. Additionally, in the early development portion of this Reading Questionnaire, parents were asked (1) at what age did your child learn to read? and (2) did your child have trouble learning how to sound-out words? We asked parents to report the age their child learned to read in years; if parents reported a year range (e.g., 3–4 years old), it was re-coded as the average (e.g., 3.5 years). The age children learned to read was a continuous variable. Parent’s response to “Did your child have trouble learning how to sound-out words?” were yes (coded 1), no (coded 0), and I don’t know (responses were removed). On these questions, data was collected at each visit. Responses were not expected to change over time but were instead used to determine parents’ internal response reliability.

Parents were also asked to complete an in-house Medical History Questionnaire focused on issues related to typical early development. Here, we focused on questions that had previously been reported to be associated with delays in early expressive language. Parents were asked to report if their child was premature, had a history of frequent ear infections, and if pressure equalizer (PE) tubes were ever placed in their child’s ears. Parent’s response choices were yes (coded 1), no (coded 0). In the cases of parents who responded “I don’t know,” responses were removed and counted as missing data. A limitation of the current study is that very few parents were able to report their child’s number of gestational weeks; as such, the number of missing data points precludes a more in-depth analysis. Parents were asked to complete the Medical History Questionnaire at time 1; at follow-up visits, parents were asked if any information regarding their child’s medical history had changed. Parents did not, nor were they expected to, report changes on the aforementioned questions; for statistical purposes, the data is considered time-invariant.

Lastly, socioeconomic status has previously been reported to impact expressive language development [40]. Two measures of socioeconomic status were collected, including the Hollingshead’s four-factor socioeconomic status score [55] and Title 1 status of the school the child attends. The Hollingshead’s four-factor socioeconomic status score [55] included parents’ highest level of educational attainment and self-reported current employment. School attended was used to determine if each child attended a school receiving Title 1 assistance. Data collected on these questions was only requested from parents at time 1, as such the data is considered time-invariant for statistical purposes.

### Assessments

At visit 1, both cohorts completed the Wechsler Abbreviated Scale of Intelligence [123, 124]. Participants completed standardized and experimental comprehension assessments at prescreening and at the four longitudinal time points (see Additional file 1: Table S1).

#### Comprehension measures

Across both studies, the Passage Comprehension subtest of the Woodcock-Johnson Test of Achievement (Cohort 2 WJ-IV: [80]; Cohort 1 WJ-III: [133]) and the Gates MacGinitie Reading Test (Gates: [43]) were used to capture reading comprehension. In Cohort 1, the Listening Comprehension subtest of the Woodcock Diagnostic Reading Battery (WDRB: [132]) was used to capture pre-test listening comprehension, while in Cohort 2, the Oral Comprehension subtest of the Woodcock-Johnson Test of Achievement (WJ-IV: [80]) was used to capture listening comprehension. Additionally, in Cohort 1, the Qualitative Reading Inventory, Fifth Edition (QRI: [72]) was included as a measure of reading and listening comprehension (see Additional file 1: Table S2), while in Cohort 2, an in-house Passage Task that mimicked the QRI was used to better control for passage genre differences, as well as passage length and the number of comprehension questions (see Additional file 1: Table S3).

#### Woodcock-Johnson III and IV tests of achievement, passage comprehension subtest (WJ-PC)

The WJ-PC subtest was administered at pre-screening for both Cohort 1 (WJ-III: [134]) and Cohort 2 (WJ-IV: [80]). Additionally, for only Cohort 2, the alternative form (Form A) of the WJ-PC subtest was administered at visits 1–3. The WJ-PC consists of 47 items that assess children’s ability to comprehend short written passages. The first four items involve matching a rebus with an actual picture of an item. The next six items involve selecting the correct picture described by a multi-word phrase. The remaining items involve a cloze format that requires children to read single- and multi-sentence passages and supply the missing word that completes the passage correctly. The passages increase in length, complexity, and vocabulary level. Basal, based on child grade, and ceiling (i.e., six consecutive items responded to incorrectly) rules were used. Median internal consistency for children ages 5–19 years is .83.

#### Gates-MacGinitie-4, reading comprehension subtest (Gates)

The Gates (Gates: [43]) was administered at three of the five time points. This comprehension test is aimed at determining how well students can read and understand entire passages. The Gates consists of 39 items in 42 stories/passages designed to assess children’s ability to read and understand passages. Different passages include both narrative and expository text. Median internal-consistency reliability is .96, and the median test-retest reliability is .88.

##### Cohort 1 visit 2 and Cohort 2 visit 1 (level 2 form S)

The initial practice item consisted of three sentences, where each sentence was followed by three pictures. After reading the sentence, children had to choose the picture that illustrates or answers a question about the text segment. Following the initial practice items, the first nine passages consisted of four text segments comprised of two to four sentences. The final passage consisted of three text segments comprised of two or three sentences each. Accompanying each text segment were three pictures. After reading the text segment, children had to choose the picture that illustrates or answers a question about the text segment. All children, following completion of a practice passage, started with the first passage and completed as many passages as possible within the time limit (35 minutes [min]).

##### Cohort 1 visit 3 and Cohort 2 visit 2 (level 3 form S)

The initial practice item consisted of a text segment comprised of six sentences, followed by three comprehension questions. Following the initial practice item, the 11 passages consisted of text segments comprised of four to ten sentences each. All children, following completion of a practice passage, started with the first passage and completed as many passages as possible within the time limit (35 min).

##### Cohort 1 visit 4 and Cohort 2 visit 3 (level 4 form S)

The initial practice item consisted of a text segment comprised of four sentences, followed by two comprehension questions. Following the initial practice item, the 11 passages consisted of text segments comprised of three to 11 sentences each. After reading the text segments, children answered three to six comprehension questions. All children, following completion of a practice passage, started with the first passage and completed as many passages as possible within the time limit (35 min).

#### Woodcock Diagnostic Reading Battery, listening comprehension subtest

The Woodcock Diagnostic Reading Battery, listening comprehension (WDRB-LC) subtest (WDRB: [132]) was administered only at pre-screening to Cohort 1. The WDRB-LC consisted of 38 items that assess children’s ability to orally comprehend sentences and short passages. There were two practice items. Listening comprehension begins with simple verbal analogies and progresses to comprehension: asking students to discern implications. Reliability ranges were reported from .81 to .98 for children between the ages of 6 and 13 years.

#### Qualitative Reading Inventory-5

The QRI [72] was administered at four of the five time points to Cohort 1. The QRI consists of narrative and expository passages ranging in length from 76 to 346 words that assess children’s ability to comprehend short heard passages. Comprehension was assessed by both retelling the passage and answering explicit (stated in the passage) and implicit (require inference) comprehension questions. Retellings are scored based on the number of idea units recalled. Comprehension questions were scored as correct or incorrect based upon the scoring template. The reliability estimate for the QRI was .94 and validity estimates of word recognition and comprehension ranged from .30 to .62. Passages were counterbalanced by modality (read vs. listened). Passages are listed in Additional file 1: Table S2 in the order administered.

#### Woodcock-Johnson IV Tests of Achievement, Oral Comprehension Subtest

The Woodcock-Johnson IV Tests of Achievement, Oral Comprehension (WJ-OC) subtest (WJ-IV: [80]) was administered at four of the five time points to Cohort 2. The WJ-OC consists of four practice items, followed by 33 test items. Children were asked to listen to passages and identify the missing keyword that makes sense. As the task progresses, passages increase from one to three sentences. Reliability ranges were reported from .78 to .82 from children between the ages of 6 and 13 years.

#### Passages task (passages)

Passages was administered at three of the five time points to Cohort 2. The passages task was developed as part of the larger longitudinal study to examine differences in narrative and expository reading and listening comprehension. Expository and narrative passages were created and matched on indices such as Level level (https://lexile.com) and word frequency (see Additional file 1: Table S3), as well as tested for equivalency using Coh-Metrix [47]. All passages were on unfamiliar topics to reduce background knowledge effects. Comprehension questions, retell procedures, and background knowledge assessments were developed. Comprehension questions are scored as correct or incorrect based upon the scoring template. Central and peripheral ideas were coded for each passage and assessed by calculating the number of each idea type recalled (i.e., coherence and elaborative inference questions). With the exception of visit 1, where children only listened to passages, passages were counterbalanced by modality (read vs. listened). Passages are listed in Additional file 1: Table S3 in the order administered.

### Diffusion imaging

Data was acquired across two Philips 3T Achieva MRI Scanners (www.usa.philips.com) at the Vanderbilt University Institute of Imaging Science. Structural T1 and DWI images at all four-time points were acquired across two Philips Achieva 3T scanners using a standard 32-channel head coil. Children from Cohort 1 were invited to participate in the neuroimaging component of our longitudinal study from time 1 (following the completion of 1st grade), time 2 (following the completion of 2nd grade), time 3 (following the completion of 3rd grade), and time 4 (following the completion of 4th grade). We included the neuroimaging data from children from Cohort 2 from time 1 (following the completion of 1st grade) and time 2 (following the completion of 2nd grade). Children watched a commercially available movie of their choice to encourage stillness and relaxation. Memory foam padding further discouraged head movement.

#### Diffusion imaging acquisition

The diffusion-weighted scan (total time: 9 min, 36.2 s) included six non-diffusion-weighted volumes (*b* = 0) and 60 diffusion-weighted volumes (i.e., 60 diffusion directions) acquired with non-collinear gradient directions (*b* = 2000 s/mm^2^), with an acquisition matrix of 96 × 94 and isotropic voxel resolution 2.5 mm^3^. The high-resolution anatomical image included 170 slices, using a multi-shot, gradient-echo sequence. The anatomical sequence included a flip angle = 8°, echo time (TE) = 4.6 ms, repetition time (TR) = 9.1 ms, field of view (FOV) = 256 × 256 (mm), and a slice thickness/gap = 1.0/0.0 mm. The diffusion-weighted scan included a flip angle = 90°, TE = 66 ms, TR = 8600 ms, FOV = 240 × 240 × 125 (mm), and a slice thickness/gap = 2.5/0.0 mm. Diffusion gradient timing (DELTA/delta) was 31.9 ms/21.8 ms.

#### Diffusion image processing

**Fig. 1:**
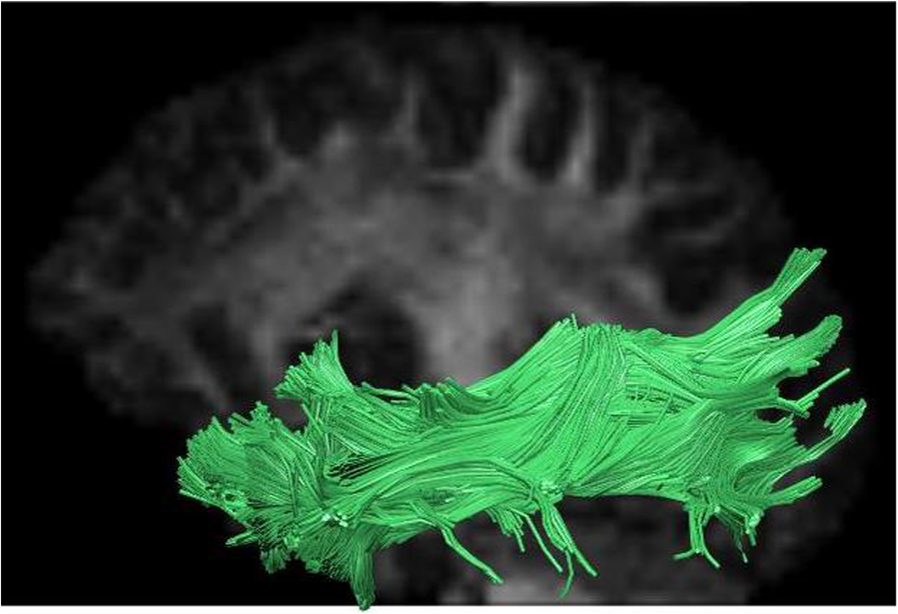
Exemplar left inferior longitudinal fasciculus tract

All data were pre-processed using Explore DTI (version 4.8.6, www.exploredti.com [71]). Diffusion volumes were corrected for eddy-current distortions and subject motion. Motion-induced artifacts were corrected via the reorientation of the b-matrix [71]. Children were excluded at a single time point due to motion artifacts in their diffusion-weighted imaging (DWI) scans, inadequate or no scan acquisitions, or use of an older eight-channel head coil. The diffusion tensor model was fitted to the data and whole-brain tractography was conducted using the following parameters: fractional anisotropy (FA) threshold = 0.2, maximum turning angle between voxels = 40°, step length between calculations = 1 mm, and a fiber length range of 50–500 mm [9]. Diffusion tensors were estimated using non-linear iterative weighting least-squares regression to identify and exclude potential outliers [20, 21] and FA (along with MD, radial/axial (RD/AD) diffusivities and total brain volume were computed) [8]. First, tracts of interest were delineated via automated atlas-based tractography using the ICBM-DTI-81 1mm atlas [82, 87]. Second, TrackVis (www.trackvis.org) was used to perform virtual, in vivo dissections of the primary tract of interest, the left-hemisphere inferior longitudinal fasciculus (ILF). Virtual dissection was performed using two regions of interest (ROIs) defined in temporal and occipital areas as outlined in Catani and Thiebaut de Schotten [19]. Tract-specific measurements quantified by the FA index were extracted for statistical analyses (see Additional file 1: Table S4). See Fig. 1 for exemplar ILF Tract.

### Statistical analyses

Analyses were performed in R (version 3.4.3) via RStudio (version 1.1.456). Statistical software code in R and output from select analyses is provided in Additional file 2 Appendix 2.

### Preliminary analyses

The R package psych (version 1.8.4, https://personality-project.org/r/psych) was used for descriptive data analyses. Correlations by time point were run in the R package ggm (version 2.3, https://CRAN.R-project.org/package=ggm) between comprehension and a composite measure of expressive language development, as well as reading and listening comprehension measures (see Additional file 2, Appendix 2: Manuscript Statistics).

### Survival analyses

Survival analysis is a class of statistics dealing with the time it takes for, or occurrences of, something (e.g., a failure, success, death, relapse) to happen [23, 62]. Here, survival analysis was used to determine the likelihood of poorer comprehension, as indexed by the occurrence of incorrect comprehension responses longitudinally. In recurrent survival analysis (also referred to as multi-spell or multi-episode survival analysis), the outcome variable of interest is a recurrent event—one that can occur more than once in the time period investigated [59, 120, 128]. Longitudinal recurrent survival analysis is characterized by the use of the whole pattern of recurrent events over time, accounting for recurrent events correlating within-subject.

A Cox (semi-parametric) proportional hazards model was used for longitudinal recurrent survival analyses (R package survival; version 2.42-6, https://CRAN.R-project.org/package=survival) allowing for determining how both time-invariant and time-varying predictors were associated with the likelihood of poorer comprehension performance [41]. Prior to all survival analyses, data was transformed into long format with four levels (i.e., passage comprehension scores nested within passage, time, and participants).

The primary outcome (or event indicator variable) across all analyses was an incorrect response to a listening or reading comprehension question on the QRI assessment and Passages task for Cohorts 1 and 2, respectively. In other words, the dependent variable is an incorrect comprehension response. An incorrect comprehension response was considered recurrent because it is possible and theoretically likely, for a child to answer a comprehension question incorrectly within or across multiple time points throughout the study. This outcome variable was measured annually from the end of 1st to the end of 4th grade. Note that at the onset of this analysis we make no claims, theoretical or statistical, about the likelihood of incorrect comprehension responses occurring at any particular time point. All survival analysis interaction effects were decomposed by displaying hazard curves for differing levels of the covariates included in the interaction [79]. The inclusion of subject as a random (frailty) effect did not improve model fit across analyses and was therefore not included (see Additional file 2 Appendix 2).

#### Survival analyses part 1

Parental questionnaire responses were used to determine the onset of childrens’ expressive language development. These early expressive language variables were time-invariant, based on expressive language development milestones that occurred prior to the first time point of the study (e.g., the onset of babbling). The expressive language development could be modeled as a categorical variable, but our assumption that it would be a continuous variable with a linear trend across the four categories performed sufficiently well. This assumption also held in the case of the expressive comprehension composite (onset of babbling + onset of speaking one’s first word + onset of putting several words together). In part 1, we aimed to determine if later expressive language development increased the likelihood of poorer language comprehension. Embedded in this analysis were two additional types of independent predictor variables, participant characteristics, and passage features. Participant characteristics included sex, age, socioeconomic status, birth, as well as a history of frequent early childhood ear infections with and without PE tubes placed (with coding schemas indicated where appropriate in the methods and results sections). Passage features included both passage modality and genre (see results for coding schemas).

#### Survival analyses part 2

Diffusion imaging collected annually was used to determine the FA in children’s left ILF white matter tract. Diffusion data was considered time-varying, as it was collected annually at each time point for Cohort 1 and was collected following 1st and 2nd grade for Cohort 2. The data was coded in 1-year intervals using a continuous-time approach. In part 2, the primary goal was to determine if FA of the left ILF corresponded to the likelihood of poorer language comprehension. A secondary goal was to determine if FA of the left ILF interacted with expressive language development, participant characteristics, or passage features predicting the likelihood of poorer language comprehension.

#### Survival analyses part 3

Early intervention was determined by parental report. Parents were asked to report if their child had received an early intervention prior to age three for a speech or language difficulty. In part 3, we aimed to determine if poorer comprehension could be decreased. The primary goal was to determine the impact of early intervention on later comprehension. A secondary goal was to assess if early intervention interacted with FA of the left ILF, expressive language development, participant characteristics, or passage features to predict the likelihood of poorer language comprehension.

#### Survival analyses 4

The presence of a speech or language disorder was also determined by parental report. Parents were asked to report if their child had a diagnosed speech or language disorder. In part 4, we aimed to determine if the likelihood of poorer comprehension could be decreased in those with a speech or language disorder. We replicated the analyses in part 3, focusing only on the subset of our sample with a speech or language disorder.

## Results

### Preliminary results

Questionnaires, standardized and experimental comprehension assessments, and diffusion imaging data were examined for adequacy of sample size, missing data, normality of the distributions, and absence of outliers. Demographic and descriptive statistics for the full sample of participants can be found in Table 1. Information regarding comprehension assessment performance by time point can be found in Table 2. Where applicable comparisons between cohorts were run to ensure that differences did not preclude collapsing analyses across both cohorts. Correlations between expressive language development and comprehension performance, as well as between FA of the left ILF and comprehension performance were run to ensure an association between the metrics. The number of individuals who showed evidence of delayed expressive language development and the number of individuals with impaired comprehension were determined to confirm an ecologically valid sample of participants; one that is representative of population estimates of those with late language emergence and those considered to be poor comprehenders.

**Table 1:** Demographic and descriptive participant information

	Time point administered					
	Prescreening	Longitudinal sample at prescreening	Time 1 (end of 1st grade)	Time 2 (end of 2nd grade)	Time 3 (end of 3rd grade)	Time 4 (end of 4th grade)
Sample Size (*N*)	1297	340	340	271	151	81
Cohort 1	821	140	140	107	85	81
Cohort 2	476	200	200	164	66	–
Age	7.08 (0.39) [5.83–8.67]	7.09 (0.35) [6.33–8.0]	7.46 (0.35) [6.42–8.67]	8.43 (0.34) [7.75–9.33]	9.42 (0.33) [8.67–10.08]	10.44 (0.33) [9.75–11.17]
Cohort 1	7.01 (0.39) [5.83–8.67]	7.03 (0.34) [6.33–8.0]	7.44 (0.34) [6.75–8.67]	8.45 (0.34) [7.75–9.08]	9.46 (0.33) [8.75–10.08]	10.44 (0.33) [9.75–11.17]
Cohort 2	7.20 (0.36) [6.33–8.33]	7.13 (0.36) [6.33–8.0]	7.47 (0.36) [6.42–8.33]	8.41 (0.34) [7.75–9.33]	9.36 (0.31) [8.67–9.83]	–
Sex	601M, 696F	159M, 181F	159M, 181F	118M, 153F	72M, 79F	35M, 46F
Cohort 1	377M, 444F	65 M, 75F	65 M, 75F	46M, 61F	44M, 41F	35M, 46F
Cohort 2	224M, 252F	94 M, 106F	94 M, 106F	72M, 92F	28M, 38F	–

Notes. () = standard deviation of the mean; [minimum, maximum]; – = data not collected

**Table 2:** Comprehension measure performance by time

	Time point administered			
	Time 1 (end of 1st grade)	Time 2 (end of 2nd grade)	Time 3 (end of 3rd grade)	Time 4 (end of 4th grade)
Reading comprehension				
WJ: Passage comprehension Raw	–	30.09 (4.45) [17–40]	33.06 (4.39) [22–42]	–
WJ: Passage comprehension SS	–	100.21 (11.12) [69–133]	100.76 (12.14) [76–130]	–
Gates: Reading comprehension Raw	28.17 (8.10) [3–39]	32.58 (8.40) [6–47]	35.22 (9.73) [10–48]	36.32 (10.69) [5–48]
Gates: Reading comprehension ESS	436.09 (42.39) [308–540]	471.58 (42.31) [344–577]	504.58 (43.90) [395–619]	524.13 (47.59) [384–619]
QRI: Ideas recalled	0.30 (0.17) [0–.93]	0.33 (0.17) [0–.84]	0.35 (0.16) [.05 – .80]	0.31 (0.14) [.02–.70]
QRI: Comprehension	0.64 (0.48) [0–1]	0.58 (0.49) [0–1]	0.65 (0.48) [0 – 1]	0.57 (0.49) [0–1]
Passages: Ideas recalled	–	0.27 (0.13) [0–.66]	0.27 (0.13) [.04 – .59]	–
Passages: Comprehension	–	0.79 (0.41) [0–1]	0.84 (0.35) [0 – 1]	–
Listening comprehension				
WJ: Oral comprehension Raw	16.55 (2.98) [10–25]	19.28 (3.36) [8–27]	20.75 (3.14) [13–28]	–
WJ: Oral comprehension SS	106.86 (10.99) [77–132]	108.62 (12.59) [63–139]	107.13 (12.37) [73–139]	–
QRI: Ideas recalled	0.30 (0.17) [0–.86]	0.31 (0.17) [0–.9]	0.32 (0.16) [.02–.87]	0.29 (0.15) [0–.70]
QRI: Comprehension	0.60 (0.49) [0–1]	0.55 (0.5) [0–1]	0.59 (0.49) [0–1]	0.54 (0.5) [0–1]
Passages: Ideas recalled	0.19 (0.11) [0–.60]	.29 (0.14) [.02–.74]	0.26 (0.11) [.04–.54]	--
Passages: Comprehension	0.67 (0.47) [0–1]	0.77 (0.42) [0 – 1]	0.84 (0.37) [0–1]	--

At time 3, data for wave 2 has not yet been collected, () = standard deviation of the mean; [minimum, maximum]; – = data not collected; *SS* standard score by age, *ESS* extended scale score

### Child history questionnaire

In response to questions from the early development portion of the Child History Questionnaire [7], parents reported the age at which their child first demonstrated three early language behaviors. Across both cohorts, 297 parents (87.4% of the total sample) reported the age at which their child first babbled (Mean [M] = 0.25, the standard deviation of the mean [SD] = 0.52, minimum [Min] = 0 [0–6 months] – maximum [Max] = 4 [4–5 years]). A total of 306 (90%) parents reported the age at which their child spoke their first word (*M* = 1.04, SD = 0.58, Min = 0 – Max = 4), and 307 (90.3%) parents reported the age at which their child first put several words together (*M* = 1.41, SD = 0.05, Min = 0 – Max = 5 [5+ years]). A Pearson correlation determined that, as expected, there was a positive association between when children began babbling and when they spoke their first word (*r* = 0.47, *t*^292^ = 9.15, *p* < .0001, 95% confidence intervals [CI] 0.38–0.55), as well as when they first put several words together (*r* = 0.35, *t*^294^ = 6.33, *p* < .0001, 95% CI 0.24–0.44). A positive association was also found between when children spoke their first word and when they first put several words together (*r* = 0.53, *t*^303^ = 11.01, *p* < .0001, 95% CI 0.45–0.61). Given the medium-to-high correlations between these variables, we used two approaches when including expressive language development in analyses. First, we created a composite measure of expressive language development timing (onset of babbling + onset of speaking one’s first word + onset of putting several words together). A higher expressive language composite indicated later expressive language development. Second, follow-up analyses were run to look at the individual effects of the developmental timing of each early expressive language milestone.

A core criterion used to identify children with late language emergence is a failure to put two or more words together by 24 months old [92, 93, 102]. In the current sample, 35 children (11.4%) would be considered to have late language emergence. Indicative of an ecologically valid sample, our percentage of those with symptoms of late language emergence is consistent with the population prevalence estimates, which range from 10 to 20% [95, 101, 138].

### Reading questionnaire

Parents were asked if their child had been diagnosed with a speech or language disorder. Of the 337 parents who responded, 41 (12.17%) indicated that their child had a previous diagnosis. Parents were asked if prior to age 3 their child received early intervention for a speech or language difficulty. Of the 318 parents who responded, 19 (5.97%) indicated that their child did receive early intervention for difficulty with speech or language. Parents were also asked some questions regarding early reading and sound development at multiple time points. Their responses were not expected to change, but the multiple responses provided a metric of parental response reliability. Specifically, parents were asked to report the age their child learned to read (*n* = 332, *M* = 4.82, SD = 1.02, Min = 1 – Max = 7). The intra-class correlation coefficient was 0.63 (*F*^44, 135^ = 7.83, *p* < .001, 95% CI 0.50–0.75). Parents were also asked if their child had trouble learning how to sound-out words. At visit 1, 79 children (23.24%) were reported to have had trouble learning to sound-out words and 52 children at visit 2 had trouble learning to sound-out words. The intra-class correlation coefficient was 0.61 (*F*^447, 141^ = 7.19, *p* < .001, 95% CI 0.47–0.73). Therefore, a high degree of reliability was found between repeated parental responses, indicating high agreement between parent responses.

### Medical history questionnaire

On the Medical History Questionnaire, parents were asked if their child was premature, had an early history of frequent ear infections, and if pressure equalizer (PE) tubes were ever placed in their child’s ears. All questions have been previously found to influence comprehension as well as effects of expressive language development (e.g., [28]). Across both cohorts, 323 parents reported that 45 children (13.32%) were premature, 57 children (16.76%) had frequent ear infections during early childhood, 35 (10.29%) of which had pressure equalizer (PE) tubes placed.

### Socioeconomic status

Socioeconomic status has also previously been shown to influence both comprehension and expressive language development. All 340 participants completed the Hollingshead’s Four-Factor Socioeconomic Status Score [55], of which 319 were scoreable (*M* = 49.94, SD = 8.95, Min = 16 [lowest SES] – Max = 62 [highest SES]). Moreover, 106 children attended a school receiving Title 1 assistance; 234 children attended a school not receiving Title 1 assistance (coded: yes = 0, no = 1). A point biserial correlation determined that there was, as expected, an association between whether children attended a Title 1 School and the Hollingshead’s SES score (*r* = 0.38, *t*(317) = 7.40, *p* < .0001, 95% CI 0.29–0.47).

### Assessments

Across both cohorts, 331 children (97.4%) completed the full-scale intelligence quotient (*M* = 105.79 SD *=* 14.79, Min = 60 – Max = 147). In Cohort 1, 138 children (98.5%) completed the full-scale intelligence quotient (*M* = 107.42, SD = 15.97, Min = 65 – Max = 147). In Cohort 2, 193 children (96.5%) completed the full-scale intelligence quotient (*M* = 104.63, SD = 13.81, Min = 60 – Max = 136). An independent-samples *t* test confirmed that no difference in full-scale intelligence existed between the cohorts (*t*^268.11^ = 1.64, *p* = 0.1; 95% CI − 0.55–6.08). Therefore, difference in intelligence quotients did not preclude collapsing analyses across cohorts. Performance on measures of listening and reading comprehension can be found in Table 2. Significant, medium-to-large effect sizes (*r* = .33–.61, *p* < .001) were found for associations between measures of reading, and measures of listening comprehension (see Additional file 1 Appendix 1, Supplemental information for details).

#### QRI and passage comprehension measures

Across cohorts and time points, four children never completed the QRI or the Passages comprehension measure, and are thus not included in subsequent analyses (sample total *n* = 336). Across all four time points, there was a significant association between comprehension performance (here, higher = better) on the QRI and the Passage Task (*r* = 0.42, *t*^818^ = 13.29, *p* < .0001, 95% CI 0.36–0.48). Moreover, the association between the QRI and the Passage Task was significant at visit 1 (*n* = 330; *r* = 0.14, *t*^329^ = 2.63, *p* < .01, 95% CI 0.04–0.25), visit 2 (*n* = 191; *r* = 0.65, *t*^251^ = 13.46, *p* < .0001, 95% CI 0.57–0.71), and visit 3 (*n* = 95; *r* = 0.64, *t*^154^ = 10.27, *p* < .0001, 95% CI 0.53–0.72). At visit 4, 80 children completed the QRI, all of whom were from Cohort 1.

The number of individuals considered to be poor comprehenders was calculated to ensure an ecologically valid sample, one that is representative of previous estimates. Note, that these are not children with specific reading comprehension deficits—those with poor comprehension and intact decoding skills [26, 61], but rather those with poor comprehension regardless of their decoding abilities [61, 64]. As in prior studies, individuals considered to be poor comprehenders are identified based on the low-tail of the distribution and by a difference of 1.5 standard deviations below the mean on the QRI or Passage Task [64]. The bottom 10% of the distribution was comprised of 33 children at visit 1, 25 children at visit 2, 15 children at visit 3, and 8 children at visit 4. When we examined the number of children who performed lower than 1.5 standard deviations below the mean, we found 23 children at visit 1, 22 children at visit 2, 13 children at visit 3, and 5 children at visit 4. As in prior studies, we found the numbers to be roughly comparable, although as noted by Keenan et al. [64] the overlap in identification is not entirely consistent.

### Comprehension correlations

Correlations were run to confirm an association between comprehension, the expressive language composite, and the FA of the left ILF. Across all four time points, there was a significant association between expressive language and comprehension (*r* = − .29, *t*^649^ = 7.71, *p* < .0001, 95% CI − 0.22–− 0.36). Moreover, the association between expressive language and comprehension increased over time. At visit 1, a significant negative correlation was found between comprehension (here, higher = better) and the expressive language development composite (higher = later to reach an expressive language milestone) *r* = − .22, *t*^287^ = 3.80, *p* < .001, 95% CI − 0.11–− 0.33. This association remained at visit 2 (*r* = − .27, *t*^223^ = 4.24, *p* < .0001, 95% CI − 0.15–− 0.39), visit 3 (*r* = − .30, *t*^134^ = 3.61, *p* < .001, 95% CI − 0.14–− 0.44), and visit 4 (*r* = − .40, *t*^60^ = 3.36, *p* < .01, 95% CI − 0.17–− 0.59). An association between comprehension and FA of the left ILF (higher = greater level of FA) was also found (*r* = 0.16, *t*^287^ = 2.82, *p* < .01, 95% CI 0.05–0.27). It was marginally significant at visit 1 (*r* = 0.12, *t*^228^ = 1.89, *p* = .059, 95% CI − 0.01–0.25), and significant by visit 4 (*r* = 0.50, *t*^48^ = 3.96, *p* < .001, 95% CI 0.25–0.69). Therefore, we can confirm an association between comprehension and our two key predictor variables, expressive language and FA of the left ILF.

A correlation was run between the expressive language composite and left ILF FA and was found to be non-significant at visit 1 (*r* = − 0.006, *t*^203^ = 0.08, *p* = 0.94, 95% CI − 0.14–0.13) and at visit 4 (*r* = − 0.073, *t*^38^ = 0.45, *p* = 0.66, 95% CI − 0.38–0.24). The onset of babbling did not correlate with visit 1 left ILF FA (*r* = 0.018, *t*^205^ = 0.25, *p* = 0.80, 95% CI − 0.12–0.15) nor did it correlate with visit 4 left ILF FA (*r* = 0.37, *t*^39^ = 0.23, *p* = 0.82, 95% CI − 0.27–0.34). The onset of speaking one’s first word and Visit 1 left ILF FA was not significant (*r* = 0.009, *t*^209^ = 0.13, *p* = 0.90, 95% CI − 0.13–0.14) and visit 4 left ILF FA was not significant (*r* = − 0.13, *t*^41^ = 0.87, *p* = 0.40, 95% CI − 0.42–0.17). The onset of putting several words together and visit 1 left ILF FA was also not significant (*r* = − 0.029, *t*^209^ = 0.43, *p* = 0.67, 95% CI − 0.16–0.11) nor was visit 4 left ILF FA (*r* = − 0.013, *t*^41^ = 0.87, *p* = 0.39, 95% CI − 0.42–0.17). Therefore, expressive language development and FA of the ILF are unrelated at the onset of the study (following the completion of first grade) and at the last visit of the study (following the completion of fourth grade).

### Survival analyses results

Cox multivariate proportional hazard survival analyses were performed to assess the effect of predictor covariates on comprehension. At every time point, events were defined as the comprehension question response (incorrect = 1, correct = 0), and were considered a separate process (as described in [3, 4]). Across both cohorts, 26 children (7.74%) did not respond incorrectly to any comprehension question at any time point. In other words, 26 children answered correctly on every single comprehension question (120 questions Cohort 1 and 128 questions Cohort 2). As such, across survival analyses, these 26 children were right-censored, which occurs when observation is terminated (e.g., the study ends) prior to an individual experiencing an event. All continuous variables were z-scored prior to model inclusion. Directionality for all categorical variables is indicated by the variable’s coding schema.

### Part 1. Does later expressive language development increase the likelihood of poorer comprehension?

First, we assessed the effect of expressive language development on language comprehension. In addition to the z-scored composite measure of expressive language development, independent (predictor) variables included both participant characteristics and passage features. Participant characteristic variables included: sex (coded: female = 1, male = 0), age (z-scored), socioeconomic status (z-scored, larger = higher SES), birth (coded: full-term = 1, premature = 0), early childhood ear infections with no PE tubes placed (coded: yes = 1, no = 0), and with PE tubes placed (coded: yes = 1, no = 0). Passage features included modality (coded: listened = 0, read = 1) and genre (coded: narrative = 0, expository= 1). Overall model fit was significant (likelihood ratio test (9) = 1311, *p* < .0001; concordance = 0.685, SE = .005).

#### Expressive language development

Children who reached expressive language development milestones later had a greater likelihood of poorer comprehension. The hazard ratio for expressive language was 1.16 (*B* = 0.15, SE = 0.01, *z* = 11.23, *p* < .001, 95% CI 0.93–1.17). Risk of poorer comprehension increased by 13.74% with each period of delay in reaching an expressive language milestone. Planned follow-up analyses determined if the onset of specific types of expressive language milestones predicted later comprehension. The likelihood of poorer comprehension increased developmentally with each specific type of expressive language milestone, i.e., babbling 7.65%, spoke first word 9.19%, and putting several words together 14.05% (see Additional file 1 Appendix 1, Supplemental information). Reaching expressive language milestones later in development conferred a greater risk of poor comprehension from the end of 1st to the end of 4th grade, particularly for developmentally later milestones (i.e., putting several words together).

#### Participant characteristics

As expected based on previous reports, socioeconomic status, age, birth, and the presence of PE tubes following frequent early ear infections all significantly predicted the likelihood of poorer comprehension. The hazard ratio for socioeconomic status was 0.80 (*B* = − 0.22, SE = 0.01, *z* = 17.75, *p* < .001, 95% CI 0.78–0.82). For each unit increase in socioeconomic status, the likelihood of poorer comprehension decreased by 20.05%. Children from high socioeconomic households answered more comprehension questions correctly. The hazard ratio for age was 0.91 (*B* = − 0.10, SE = 0.04, *z* = 2.17, *p* < .05, 95% CI 0.83–0.99). For each unit increase in age, the likelihood of poorer comprehension decreased by 9.16%. Children who were older following completion of each grade answered more comprehension questions correctly. The hazard ratio for birth was 0.86 (*B* = − 0.15, SE = 0.04, *z* = 4.26, *p* < .001, 95% CI 0.80–0.92). Children born at full term have a 13.99% decreased likelihood of poorer comprehension. Full-term children answered more comprehension questions correctly than those born prematurely. The hazard ratio for PE tubes was 0.79 (*B* = − 0.23, SE = 0.05, *z* = 4.61, *p* < .001, 95% CI 0.72–0.87). Children with PE tubes have a 20.84% decreased likelihood of poorer comprehension. Children with a frequent history of ear infections who have PE tubes answered more comprehension questions correctly. Additionally, sex was a marginally significant predictor of poorer comprehension. The hazard ratio for sex was 0.95 (*B* = − 0.05, SE = 0.03, *z* = 1.93, *p* = .05, 95% CI 0.90–1.00). Female children had a 5.28% decreased likelihood of poor comprehension compared to male children. Female children answered more comprehension questions correctly than male children. Additionally, frequent ear infections that were not treated with PE tubes were not found to predict comprehension (*p* = .52).

#### Passage features

As expected, poorer comprehension was predicted by passage features. The hazard ratio for modality was 0.87 (*B* = − 0.13, SE = 0.03, *z* = 4.63, *p* < .001, 95% CI 0.83–0.93). Reading, as opposed to listening to, passages decreased the likelihood of poor comprehension by 12.63%. Children answered more comprehension questions correctly when reading passages. The hazard ratio for genre was 2.18 (*B* = 0.78, SE = 0.03, *z* = 27.06, *p* < .001, 95% CI 2.06–2.31). Expository, as compared to narrative, passages increased the risk of poor comprehension by 54.19%. Children answered more comprehension questions correctly when listening or reading a narrative passage.

##### Modality

The first set of planned follow-up analyses focused on determining if listening and reading comprehension independently were predicted by the expressive language development composite and if any of the participant characteristic variables differed when predicting listening versus reading comprehension. Children who reached expressive language milestones later had an increased risk of poorer comprehension for both reading (17.59%) and listening (11.57%) comprehension (*p* < .001). Moreover, the likelihood of poorer listening comprehension increased significantly (*p* < .001) and developmentally with each specific type of expressive language milestone, i.e., babbling 5.97%, spoke first word 7.39%, and putting several words together 13.17%. The same was true for the likelihood of poorer reading comprehension (*p* < .001): babbling 10.71%, spoke first word 12.17%, and putting several words together 15.47%. While socioeconomic status (*p* < .001) and birth (*p* < .05) continued to predict both reading and listening comprehension (see Additional files 1 and 3 Supplementary methods for details ), two participant characteristics differed when predicting listening versus reading comprehension, early childhood ear infections with PE tubes placed and age following grade completion. Children with PE tubes had a 26.65% decreased likelihood of poorer listening comprehension (*p* < .001), but not reading comprehension (*p* = .18). Additionally, children who were older following grade completion had a 17.55% decreased likelihood of poorer reading comprehension (*p* < .01), but not listening comprehension (*p* = .43).

##### Genre

The second set of planned follow-up analyses focused on determining if expository and narrative comprehension independently were predicted by the expressive language development composite and if any of the participant characteristics differed when predicting expository versus narrative comprehension. Children who reached expressive language milestones later had an increased risk of poorer comprehension for both expository (10.78%) and narrative (18.66%) comprehension (*p* < .001). Moreover, the likelihood of poorer narrative comprehension was significant for all three expressive language milestones (*p* < .001): babbling 7.54%, spoke first word 14.35%, and putting several words together 21.30%, as was the likelihood of poorer expository comprehension (*p* < .001): babbling 7.64%, spoken first word 6.28%, and putting several words together 9.80%. The milestones contributed more equally to poorer expository comprehension. While socioeconomic status (*p* < .001), birth (*p* < .001), and early childhood ear infections with PE tubes placed (*p* < .001) continued to predict both expository and narrative comprehension (see Additional files 1 and 3 Supplementary methods for details), one participant characteristic differed when predicting expository versus narrative comprehension. Children who were older following grade completion had an 11.78% decreased likelihood of poor expository comprehension (*p* < .05), but not narrative comprehension (*p* = .61).

### Part 2. Does fractional anisotropy of the left ILF correspond to the likelihood of poorer comprehension?

In part 1, comprehension from the end of 1st through the end of 4th grade was predicted by expressive language development, socioeconomic status, age, birth, and the presence of PE tubes in those with an early history of frequent ear infections, as well as modality and genre of the passage. Here, building upon the previous survival analysis, we assessed the effect of the left ILF FA on later language comprehension. As in the previous model, the dependent variable (event) was comprehension question response (coded: incorrect = 1, correct = 0). The final model’s independent variables were the same as in the previous analysis, but also included FA of the left ILF (z-scored) as a time-varying predictor. We also aimed to determine if the amount of FA interacted with current predictors of comprehension. Backward fitting model techniques were used to remove all non-significant interactions. As such, three significant two-way interactions were included: (1) FA and expressive language composite, (2) FA and socioeconomic status, and (3) FA and sex. Overall model fit was significant (likelihood ratio test (13) = 864.6, *p* < .0001; concordance = 0.676, SE = .006).

#### Left ILF (FA)

The likelihood of poorer comprehension was indeed predicted by the amount of FA in the left ILF. The hazard ratio for FA was 0.94 (*B* = − 0.05, SE = 0.03, *z* = 2.08, *p* < .05, 95% CI 0.90–1.00). For each increase in FA, the likelihood of poorer comprehension decreased by 5.1%, indicating that children with higher FA answered more comprehension questions correctly.

#### Interaction of the expressive language composite and FA

**Fig. 2:**
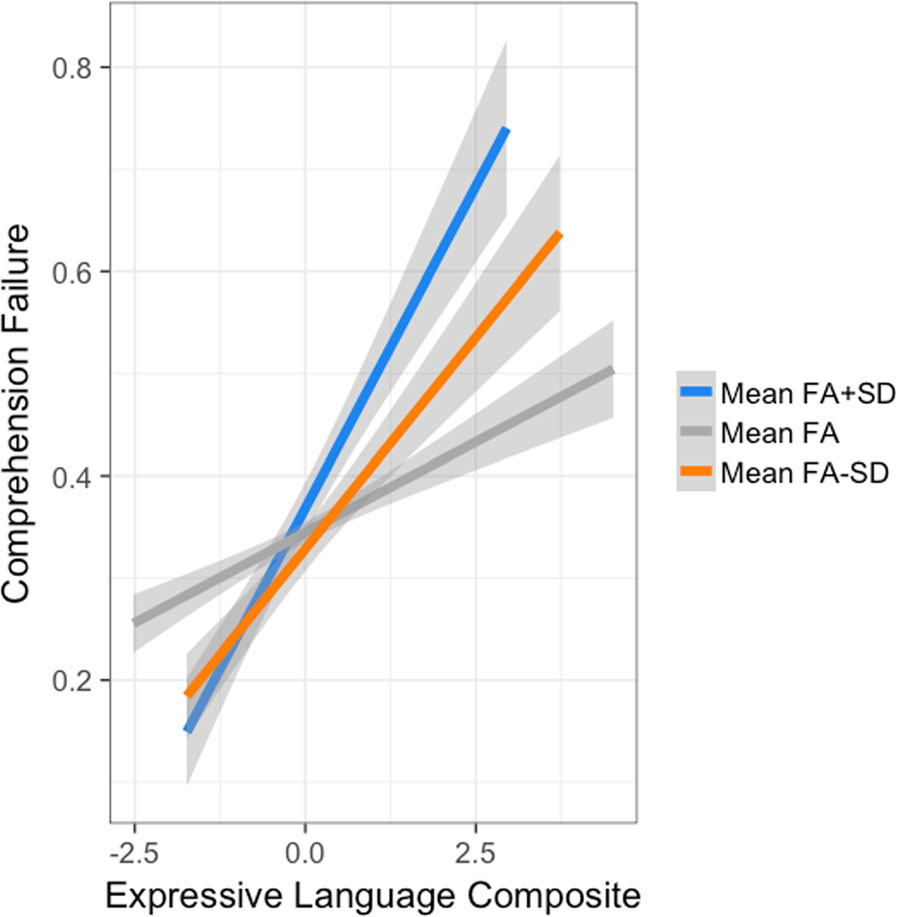
Interaction of the expressive language composite and the fractional anisotropy of the left ILF. The amount of FA in the left ILF was found to moderate the relationship between expressive language development and the likelihood of poorer comprehension

The amount of FA in the left ILF was found to moderate the relationship between expressive language development and the likelihood of poorer comprehension. The interaction had a hazard ratio of 1.05 (*B* = 0.05, SE = 0.02, *z* = 3.266, *p* < .01, 95% CI 1.02–1.08). As recommended by Aiken and West [2], to decompose the interaction and further facilitate its interpretation, hazard probabilities for expressive language development were plotted at the mean FA of the left ILF, one standard deviation below the mean (hazard ratio = 1.23, *B* = 0.20, SE = 0.02, *z* = 8.94, *p* < .001, 95% CI 1.17–1.28), and one standard deviation above the mean (hazard ratio = 1.11, *B* = 0.10, SE = 0.02, *z* = 4.66, *p* < .001, 95% CI 1.06–1.16). FA functioned as an exacerbator between expressive language development and the likelihood of poorer comprehension (see Fig. 2). Children with the highest FA yielded the steepest positive slope between expressive language development and the likelihood of poorer comprehension. The effect of FA as a moderator made less difference for those children with early expressive language development, but exerted a much stronger influence on children with later expressive language development. Those who had the poorest comprehension had reached expressive language development milestones the latest and had the highest FA, while those with the best comprehension had reached expressive language development milestones earlier and also had the highest FA.

Planned follow-up analyses for each expressive language development milestone revealed that the interaction of expressive language development and FA was driven by the developmental onset of putting several words together (*p* < .001) and babbling (*p* < .01), but not when children spoke their first word (*p* = .73) (see Additional file 1 Appendix 1, Supplemental information). FA functioned as an exacerbator between the likelihood of poorer comprehension, putting several words together and babbling. For both expressive language milestones, children with the highest levels of FA had the steepest positive slopes. It was only for the expressive language milestone—putting several words together, that children with the lowest FA had a steeper positive slope than those children whose z-scored FA values ranged within a standard deviation.

#### Interaction of socioeconomic status and FA

There was a significant interaction between the left ILF FA and socioeconomic status. The interaction had a hazard ratio of 1.03 (*B* = 0.03, SE = 0.01, *z* = 2.35, *p* < .05, 95% CI 1–1.06). To decompose the interaction, hazard probabilities for socioeconomic status were plotted at the mean FA of the left ILF, one standard deviation below the mean FA (hazard ratio = 0.83, *B* = − 0.19, SE = 0.02, *z* = 9.08, *p* < .001, 95% CI 0.79–0.86), and one standard deviation above the mean (hazard ratio = 0.77, *B* = − .26, SE = 0.02, *z* = 12.67, *p* < .001, 95% CI 0.74–0.80). FA functioned as a buffer between childhood socioeconomic status and the likelihood of poorer comprehension (see Additional file 1: Figure S1). Children with the highest levels of FA yielded the weakest negative slope between socioeconomic status and the likelihood of poorer comprehension, while the strongest slope was obtained by children with the lowest levels of FA.

#### Interaction of sex and FA

There was a significant interaction between sex and FA, which had a hazard ratio of 1.09 (*B* = 0.08, SE = 0.03, *z* = 2.59, *p* < .01, 95% CI 1.02–1.16). For each unit increase in FA, male children had a 7.94% increase in the likelihood of poorer comprehension compared to female children. In other words, male children with a greater amount of FA in the left ILF were more likely to answer comprehension questions incorrectly.

Finally, irrespective of the inclusion of the interactions in the model, poorer comprehension remained predicted by the expressive language development composite (*p* < .001), socioeconomic status (*p* < .0001), age (*p* < .05), birth (*p* < .05), the presence of PE tubes (*p* < .01), and the modality (*p* < .05) and genre (*p* < .0001) of the passage (see Additional file 2 Appendix B for full model output).

### Part 3. How do you decrease the likelihood of poorer comprehension?

We asked parents to report if their child had an intervention prior to age three for issues with speech and language. Previous research has found that while some children with delays in early expressive language catch up to their peers, others remain delayed (e.g., [39]). As such, whether or not to provide early intervention to children with delays in early expressive language development has been the subject of much controversy, and early intervention is typically only administered in the most clearly delayed cases of expressive language development (e.g., [39]). Therefore, not all of the children whose parents reported later onsets of expressive language development received intervention. Preliminary analyses revealed a significant positive association between early intervention (coded: yes = 1, no = 0), and the expressive language development composite (*r* = .22, *t*^282^ = 3.70, *p* < .001, 95% CI 0.10–0.32). This confirms that, at least in the current sample, there was an association between early issues with expressive language development and receiving early intervention. However, it also indicates that those who received early intervention likely demonstrated more severe issues with expressive language development.

Building again upon the previous analysis, we assessed the effect of receiving intervention prior to age three on later comprehension. As in the previous analyses, at every time point, each event (comprehension question response: incorrect = 1, correct = 0) was considered a separate process. We also aimed to determine if receiving early intervention interacted with previously identified comprehension predictors. Backward fitting model techniques were used to remove all non-significant effects and interactions. As such, two additional significant interactions were included: a two-way interaction of the FA and early intervention, and a three-way interaction of the FA, early intervention, and the expressive language development composite. Overall model fit was significant (likelihood ratio test (16) = 861.5, *p* < .0001; concordance = 0.677, SE = .006).

#### Early Intervention

The likelihood of poorer comprehension was predicted by receiving early intervention. The hazard ratio for receiving early intervention is 1.25 (*B* = 0.22, SE = 0.07, *z* = 2.97, *p* < .01, 95% CI 1.07–1.44). As indicated by the preliminary correlations, those who received early intervention had a 19.69% increase in the likelihood of poorer comprehension. This is likely due to the children who received early intervention having the greatest issues with expressive language development.

#### Interaction of the expressive language composite, FA, and early intervention

**Fig. 3.**
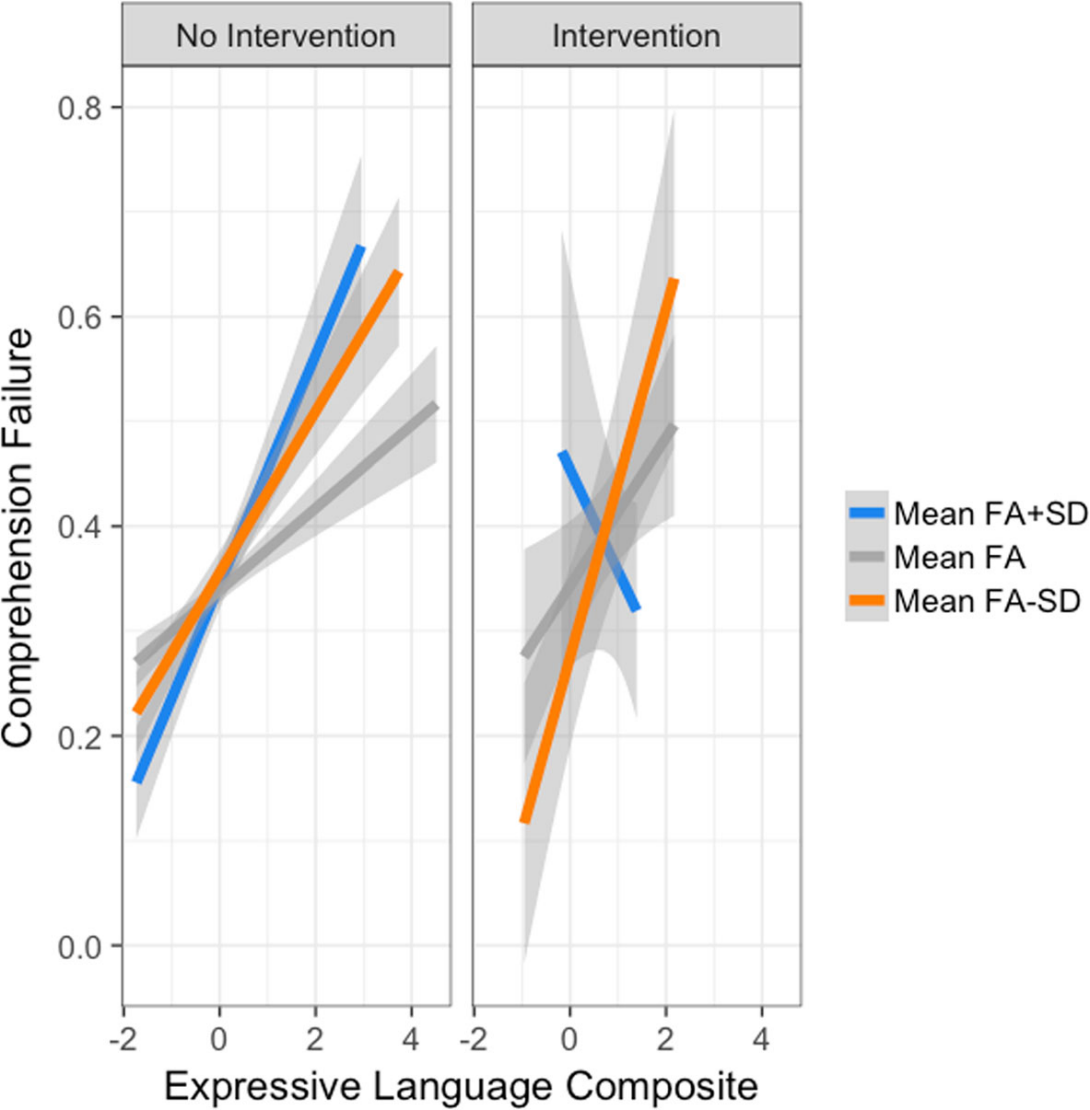
Interaction of the expressive language composite, fractional anisotropy of the left ILF, and early intervention. The left ILF was found to differentially moderate the relationship between expressive language development and poorer comprehension based on receipt of early intervention

The left ILF was found to moderate the relationship between expressive language development and poorer comprehension for both those who had received and those who had not received early intervention (Fig. 3). As such, the three-way interaction of early intervention, the expressive language development composite, and left ILF had a hazard ratio of 0.58 (*B* = 0.55, SE = 0.12, *z* = 4.53, *p* < .001, 95% CI 0.46–0.73).

##### No intervention

To decompose the interaction and further facilitate its interpretation, hazard probabilities for expressive language development for those who did not receive intervention were plotted at the mean FA of the left ILF, one standard deviation below the mean FA (hazard ratio = 1.21, *B* = 0.19, SE = 0.05, *z* = 3.68, *p* < .001, 95% CI 1.10–1.35), and one standard deviation above the mean FA (hazard ratio = 1.17, *B* = 0.17, SE = 0.09, *z* = 1.91, *p* = .057, 95% CI 0.99–1.42) (see Fig. 3, left panel). The left ILF functioned as an exacerbator between expressive language development and the likelihood of poorer comprehension for those who had not received early intervention. Again here, we see that the effect of FA as a moderator made less difference for those children with early expressive language development, but exerted a much stronger influence on children with later expressive language development. Children who had not received early intervention, who had the highest amount of FA yielded the steepest positive slope between expressive language development and the likelihood of poorer comprehension. Of those who did not have early intervention, children with the poorest comprehension had both the greatest delay in expressive language development and the highest FA, followed by those with the lowest FA and finally those with average FA. Children with the best comprehension had the least delay in expressive language development and the highest FA, followed by those with the lowest FA, and finally those with average FA.

##### Intervention

Hazard probabilities for expressive language development for those who did receive intervention were plotted at the mean FA of the left ILF, one standard deviation below the mean FA (hazard ratio = 0.82, *B* = 0.20, SE = 0.05, *z* = 3.68, *p* < .001, 95% CI 0.74–0.91), and one standard deviation above mean FA (hazard ratio = 0.84, *B* = − 0.17, SE =0.09, *z* = 1.91, *p* = .056, 95% CI 0.70–1.00) (see Fig. 3, right panel). For those children who received early intervention, the left ILF FA functioned as a buffer between expressive language development and the likelihood of poorer comprehension. Children who received early intervention and had the lowest levels of FA yielded the steepest positive slope between expressive language development and the likelihood of poorer comprehension. Of those who did have early intervention, children with the poorest comprehension had the greatest delay in expressive language development and the lowest FA. The relationship was inverted for those with the highest FA; high FA children with the poorest comprehension had the least delay in expressive language development.

Follow-up analyses of the three-way interaction by type of expressive language development revealed that the interaction of early intervention, onset of expressive language development, and FA was driven by the developmental onset of putting several words together (*p* < .01), speaking one’s first word (*p* < .01), and babbling (*p* < .01; see Additional file 1 Appendix 1, Supplemental information). Moreover, as in the previous analyses, the likelihood of poorer comprehension was predicted by expressive language development (*p* < .001), FA (*p* < .05), socioeconomic status (*p* < .0001), age (*p* < .01), birth (*p* = .058), PE tube presence (*p* < .01), as well as modality (*p* < .01) and genre (*p* < .0001) of the passage (see Additional file 2 Appendix B for details).

### Part 4. How do you decrease the likelihood of poorer comprehension in those with a speech or language disorder?

In addition to asking parents to report if their child had an intervention prior to age 3 for issues with speech and/or language, we also asked if their child had later been diagnosed with a speech and/or language disorder. Based upon parental report, 41 children (12.17%) had a diagnostic history of a speech and/or language disorder, 19 of whom had previously (prior to age 3) received an early intervention for issues with speech and/or language. A point biserial correlation analysis confirmed that there was a significant positive association between if children had an early intervention and if they were later diagnosed with a speech and/or language disorder (coded: yes = 1, no = 0) *r* = .19, *t*^316^ = 3.51, *p* < .001, 95% CI 0.08–0.30. As a significant positive relationship was found, we assessed the effect of receiving an early intervention on later comprehension, in only those who were diagnosed with a speech and/or language disorder.

A survival analysis was run on only the subset of individuals who had been diagnosed with a speech and/or language disorder. Across those 41 participants, there was a total of 2450 unique comprehension question responses. As in the previous analyses, at every time point, each event (comprehension question response: incorrect = 1, correct = 0) was considered a separate process. Backward fitting model techniques were used to remove all non-significant effects and interactions. The following predictor covariates remained in the model: socioeconomic status, expressive language development composite, genre, and receiving early intervention prior to age three. The remaining interaction, between sex and the left ILF, was only marginally significant. Overall model fit was significant (likelihood ratio test (6) = 199.6, *p* < .0001; concordance = 0.75, SE = .015).

#### Early intervention

The likelihood of poor comprehension was predicted by receiving early intervention. The hazard ratio for receiving early intervention is 0.61 (*B* = − 0.49, SE = 0.18, *z* = 2.75, *p* < .01, 95% CI 0.43–0.87). In the prior whole-sample analysis, receiving early intervention seemed to indicate the severity of issues in early language development, where intervention (increased severity) corresponded to an increased likelihood of poorer comprehension. However, within the current sample of children—only those children later diagnosed with a speech and/or language disorder—those who received early intervention had a 38.99% decrease in their likelihood of poorer comprehension. Therefore, we can conclude that for those with speech and language disorders, the impact of early intervention is the reduction of poorer comprehension from the end of 1st to the end of 4th grade. In other words, those who are later diagnosed with speech and language disorders would have benefitted from early intervention for speech and language issues.

#### Interaction of sex and FA

There was also a marginally significant interaction of sex and FA in the left ILF. This interaction mirrored the results of the whole-sample analysis. Females had a hazard ratio of 0.91 (*B* = − 0.10, SE = 0.05, *z* = 1.86, *p* = .06, 95% CI 0.82–1.01). For each unit increase in FA in the left ILF, female children with a speech and/or language disorder had a 9.44% decrease in the likelihood of poorer comprehension.

#### Expressive language development

Children with a speech and/or language disorder who reached expressive language development milestones later had a greater likelihood of poorer comprehension. The hazard ratio for expressive language was 1.33 (*B* = 0.28, SE = 0.05, *z* = 6.10, *p* < .001, 95% CI 1.21–1.46). Thus, for those with a speech and/or language disorder, risk of poorer comprehension increased by 24.73% with each period increase in reaching an expressive language milestone. This indicates that in children with speech and/or language disorders, risk of poorer comprehension is almost double that of the whole sample for each period increase in reaching an expressive language milestone. Follow-up analyses determined if the onset of specific types of expressive language milestones predicted later comprehension in those children diagnosed with a speech and/or language disorder. As in the whole-sample analysis, the likelihood of poorer comprehension for children diagnosed with a speech and/or language disorder increased developmentally with each specific type of expressive language milestone, i.e., babbling 9.36%, spoke first word 18.61%, and putting several words together 31.65% (see Additional file 1 Appendix 1, Supplemental information). Reaching expressive language milestones later in development continued to confer a greater risk of poor comprehension from the end of 1st to the end of 4th grade, particularly for developmentally later milestones (i.e. putting several words together).

#### Participant characteristics

The hazard ratio for socioeconomic status was 0.82 (*B* = − 0.19, SE = 0.03, *z* = 6.637, *p* < .001, 95% CI 0.78–0.87). For each unit increase in socioeconomic status, the likelihood of poorer comprehension in children with a diagnosed speech and/or language disorder decreased by 17.59%. Even when children have a diagnosed speech and/or language disorder, those with a high socioeconomic status answered more comprehension questions correctly.

#### Passage features

The hazard ratio for genre was 2.36 (*B* = 0.86, *SE* = 0.10, *z* = 8.73, *p* < .001, 95% CI 1.94–2.86). Expository, as compared to narrative, passages increased the risk of poor comprehension by 54.19%. Children with a speech and/or language disorder answered more comprehension questions correctly when the passage was narrative.

##### Genre

Planned follow-up analyses of only those children diagnosed with a speech and/or language disorder focused on determining if expository and narrative comprehension were independently predicted by the expressive language development composite and if any of the participant characteristics differed when predicting expository versus narrative comprehension. As in the whole-sample analysis, children who reached expressive language milestones later had an increased risk of poorer comprehension for both expository (25.80%) and narrative (24.47%) comprehension (*p* < .001). Follow-up analyses revealed that the likelihood of poorer expository comprehension for children diagnosed with a speech and/or language disorder increased developmentally with each specific type of expressive language milestone, i.e., babbling 13.39% (*p* < .05), spoke first word 17.66% (*p* < .001), and putting several words together 29.97% (*p* < .001). While the same pattern held true for the likelihood of poorer narrative comprehension for children diagnosed with a speech and/or language disorder, spoke first word 21.24% (*p* < .001), and putting several words together 34.65% (*p* < .001). However, babbling was not significant (*p* = .70).

The effect of receiving early intervention on comprehension (for only those individuals with a speech and/or language disorder) was driven by the expository condition. The hazard ratio for early intervention on only the expository condition was 0.58 (*B* = − 0.54, SE = 0.22, *z* = 2.50, *p* < .05, 95% CI 0.38–0.89). For those children later diagnosed with a speech and/or language disorder who received early intervention, the likelihood of poorer expository comprehension decreased by 41.54%.

## Discussion

The present study was aimed at understanding the impact of expressive language development, a neurobiological correlate—the left ILF, and the impact of early intervention, prior to age 3, on children’s comprehension during primary school. In both theory and practice, whether or not to administer early intervention for later comprehension difficulty is a key question; one that may be guided by understanding the structural neurobiology of later reading comprehension success. To evaluate this, four core questions were addressed. First, we replicated and extended previous findings by examining the influence of the timing of expressive language development milestones on later comprehension. Then we, for the first time, investigated if the left ILF acted as a potential neurodevelopmental correlate of comprehension, as well as if the left ILF moderated the effect of expressive language on comprehension. Third, we sought to determine if children who received intervention services prior to 3 years old for early issues with speech and/or language, including delayed expressive language, would have better comprehension than those children with issues who did not receive early intervention. Finally, we examined the impact of receiving early intervention services on the comprehension of children diagnosed in primary school with a speech and/or language disorder.

### Expressive language development impacts comprehension

The majority of studies on the impact of expressive language development focused on later language development at 5, 6, or 7 years old [99]. However, a handful of studies have reported that primary school children who had reached expressive language milestones later have difficulty with the higher linguistic demands of listening and reading comprehension [14, 70, 92, 137]. In the present study, we replicated and extended those findings, by examining the onset of expressive language milestones as predictors of the likelihood of poorer comprehension later in development, following the completion of 1st–4th grade. We first replicated and extended previous findings, using a longitudinal cohort design and survival analyses. Children who took longer to reach early expressive language milestones did, in fact, have poorer comprehension during primary school. When comprehension modality was examined separately, children with later expressive language milestones were found to have poorer listening and reading comprehension. As the first study to examine the relationship between expressive language development and expository passage comprehension, we found that when comprehension genre was examined separately, children with later expressive language milestones had poorer expository and narrative comprehension. Moreover, regardless of these passage characteristics, the impact of expressive language development on comprehension was found to be driven by all three expressive language milestones: babbling, speaking one’s first word, and putting several words together. Notably, a greater risk of poorer comprehension was conferred by later developmental milestones (i.e., putting several words together), than by earlier developmental milestones (i.e., babbling) for all passage features except for expository comprehension.

### A neurodevelopmental correlate of comprehension

The second core aim of this study was to determine if a white matter tract associated with early language development and ability would predict the likelihood of poorer comprehension. The ILF is located along the central portion of the occipital and temporal lobes and has been proposed to serve as an indirect pathway for the transfer of semantic information during language processing [31, 32, 78]. Results indicated that poorer comprehension was predicted by the amount of FA of the left ILF. Specifically, children with higher FA answered more comprehension questions correctly.

### The left ILF moderated the impact of early expressive language development on later comprehension

The left ILF was found to moderate the relationship between reaching early expressive language milestones and comprehension during primary school, serving as an exacerbator. The effect of FA as a moderator made less difference for those children with early expressive language development but exerted a much stronger influence on those with later expressive language development. Children with the highest amount of FA had the steepest positive slope between the expressive language composite and poorer comprehension. As such, for children who reached their expressive language milestones earliest, those with the highest FA had the best comprehension performance, followed by those with the lowest FA, and those with average FA performed the poorest. In contrast, for children who reached their expressive language milestones latest, those with the highest FA had the worst comprehension performance, followed again by those with the lowest FA, while those with average FA performed the best. Additionally, when expressive language milestones were examined individually, the effect was found to be driven by the development onset of putting several words together and babbling, but not when children spoke their first word. Taken together, this suggests that the strength of the relationship between comprehension performance in primary school and early expressive language development, particularly the developmental onset of putting several words together and babbling, is altered by the development of FA. This may indicate that children with differences in left ILF FA are using different cognitive strategies, or relying on different reading circuits to perform comprehension tasks.

### Early intervention as an indicator of speech and language severity

As noted early on by Fischel et al. [39], “One of the most difficult tasks in developmental pediatrics is the differentiation of developmental anomalies that deserve intervention from those that do not.” They provide the example of a “thumb-sucking” 3-year-old child as a statistical outlier, but one for which evidence does not exist suggesting that the child’s future development is at risk. A more controversial case is found in the developmental timing of reaching expressive language milestones. While some children with delays in expressive language milestone onset do appear to catch-up to their peers, some remain substantially delayed in language production [33, 39, 100]. This has led many to suggest a “wait-and-see” approach and to consider these children “late bloomers” (e.g., [119]). Our preliminary results indicated a positive association between receiving early intervention and expressive language development. The directionality of the association indicated that those who received early intervention likely demonstrated more severe early issues with speech and language. Results of the survival analysis were consistent with the preliminary correlation analysis, those who received early intervention, prior to age 3 years old, had an increased likelihood of poorer comprehension. While this could, of course, be interpreted as early intervention leading to poorer comprehension, it is more likely the impact of severity of early issues with speech and/or language that is leading to poorer comprehension.

### Early intervention differentiates the moderating effect of the left ILF on the relationship between early expressive language development and later comprehension

We found that the left ILF moderated the relationship between expressive language development and poorer comprehension for those who did, and those did not, receive early intervention. Intriguingly, the moderating effect of FA on the relationship between the onset of expressive language development milestones and comprehension was differentiated by the impact of receiving an early speech and/or language intervention. The left ILF functioned as an exacerbator between expressive language development and poorer comprehension for only those children who did not receive early intervention. Yet, the results from the children who received intervention do deviate. For those children who did receive an intervention, the ILF functioned as a buffer, diminishing the relationship between the onset of expressive language development and poorer comprehension. Children with the lowest FA who received intervention and had the poorest comprehension had the greatest delay in expressive language development. The relationship was inverted for those with the highest FA; children with the highest FA who received intervention and had the poorest comprehension had the least delay in expressive language development. In other words, for those who did receive early speech and/or language intervention services, those with higher FA had a weaker relationship between expressive language development and comprehension. This suggests that early intervention diminishes and potentially alters the relationship between early expressive language development, later reading comprehension and left ILF FA.

### Early interventions for those later diagnosed with speech or language disorders

In the whole sample analysis, receiving an early intervention seemed to indicate the severity of the early speech and/or language issues experienced. However, since the majority of the children in our study did not have an early intervention and (consistent with prevalence reports) difficulty with speech and/or language requiring intervention prior to age 3, we followed up our initial finding by investigating only those children who were later diagnosed with a speech and/or language disorder. Put more simply, we investigated the impact of early intervention on those for whom early speech and/or language intervention services may have been needed and impactful.

Preliminary analyses revealed that a little less than half of those children who were diagnosed with a speech or language disorder during primary school had previously received early intervention. Therefore, a separate survival analysis focused on only those who were later diagnosed with a speech or language disorder. We found that early intervention decreased their risk of poorer comprehension by almost 40%. This suggests that children who were later diagnosed with a speech or language disorder would have benefited from early intervention services.

### The impact of participant characteristics on comprehension

A number of participant characteristics have previously been linked to the impact of expressive language development and language comprehension. Here, we found that comprehension performance was also predicted by a number of child characteristics, including socioeconomic status, age following the completion of each grade, sex, the presence of PE tubes following frequent early ear infections and the gestation period prior to birth.

#### Socioeconomic status

The extant data on socioeconomic status has linked it to both reading comprehension (e.g. [16, 51, 86]) and expressive language development (e.g. [40, 50, 81]). In the current study, children from a higher socioeconomic status answered more comprehension questions correctly. When modality and genre were examined separately, children from a higher socioeconomic status answered more listening and reading comprehension questions correctly, as well as more expository and narrative comprehension questions correctly. Thus, regardless of the passage characteristics, higher socioeconomic status decreased the likelihood of poor comprehension. Even in the subsample of children who were later diagnosed with a speech or language disorder, those at higher socioeconomic status answered more comprehension questions correctly. Additionally, the left ILF was found to serve as a buffer, moderating the relationship between socioeconomic status and comprehension. Children with the highest amount of FA had the weakest negative slope between socioeconomic status and poorer comprehension; children with the lowest amount of FA had the strongest negative slope between socioeconomic status and poor comprehension. Therefore, the strength of the impact of socioeconomic status on passage comprehension is the weakest for those with the highest levels of left ILF FA. In other words, socioeconomic status affects passage comprehension the least when children’s FA in the left ILF is high.

#### Age following grade completion

Children who were older following the completion of each grade answered more comprehension questions correctly. When comprehension modality was examined separately, increased age decreased the likelihood of poor reading comprehension but not listening comprehension. As noted in the introduction to this paper, differences between children’s performance on comprehension across modality decreased over time as a result of print exposure, word decoding mastery, and as a function of age. It may be the case that children who were older following the completion of each grade had more print exposure or slightly higher word decoding mastery. Moreover, when comprehension genre was examined separately, increased age decreased the likelihood of poor expository comprehension but not narrative comprehension. Expository passages are generally considered to be harder to comprehend than narrative passages, placing a higher demand on, among other things, the reader’s background knowledge (e.g., [48]). Thus, it may also be the case that children who were older following the completion of each grade had more background knowledge to draw from, facilitating expository comprehension. Here, we chose to examine the effect of comprehension following each grade in an effort to control for classroom reading instruction effects. Future studies may instead want to examine differences in passage features on comprehension performance using age, rather than grade, matched samples.

#### Sex differences

Differences in comprehension performance between male and female children have been the focus of a number of studies (e.g., [42]) and have been found to exist worldwide [74]. As in prior studies, here we found that female children answered more comprehension questions correctly than male children. This remained true even in the sub-sample later diagnosed with a speech or language disorder. A core precursor to children’s comprehension performance is word decoding mastery. Difficulty with word recognition and decoding has been linked to sex differences: the bottom fifth percentile of decoders consists of 7.3% males, whereas only 2.8% are female [107]. Our results likely indicate that sex differences in comprehension are, in part, related to earlier differences in decoding mastery.

We also found sex differences between the association of the left ILF and reading comprehension. Male children with greater FA were more likely to answer comprehension questions incorrectly that female children. Lebel and Beaulieu [68] have shown that the ILF has a prolonged maturation period one that continues into post-adolescence, from 5 to 22 years old. Critically, they found no sex differences in the developmental trajectory of FA in the ILF. This stands in contrast to sex differences found in the FA of the splenium of the corpus callosum, cingulum, corticospinal tracts, superior longitudinal fasciculus, and uncinate fasciculus. This suggests that less FA, a parameter linked to axon packing and myelination [10], may better facilitate comprehension in males.

#### Ear infections and pressure equalizer (PE) tubes

In the United States, an estimated one out of every 15 children will have PE tubes placed prior to their third birthday [65]. In the current study, we found that while frequent early ear infections did not impact comprehension performance, when those ear infections were treated with PE tubes children’s risk of poor comprehension decreased. PE tubes have been found to decrease the frequency of ear infections that persist after antibiotic treatment by allowing air in, and fluid to drain out of the middle ear, which restores and preserves normal hearing [24]. Here, we found that the effect persists across both expository and narrative comprehension, and was driven by listening comprehension performance. This is consistent with previous reports suggesting that PE tube placement allows children to recognized words at lower listening levels (e.g., [106]), despite lower average scores on both verbal comprehension and expressive language at ages 3, 5, 7, and 9 years old [112].

#### Full-term versus premature birth

Children who were born full-term answered more comprehension questions correctly, regardless of the modality or genre of the passage. While this association is consistent with previous results, a noted limitation of the current study is that very few parents were able to report their child’s number of gestational weeks; as such, the number of missing data points precluded a more in-depth analysis (see Limitations).

### Passage features

Comprehension performance was predicted by passage features. This included both modality (listening versus reading) and genre (expository versus narrative) passage features. Children answered more comprehension questions correctly when reading passages than when listening to passages. It has been well established that the relationship between listening and reading comprehension changes as a function of decoding mastery and age (e.g., [25, 30, 90, 116, 117]). For example, Diakidoy et al. [30] replicated previous results showing that reading comprehension becomes stronger than listening comprehension following the mastery of decoding. They also found that the reading comprehension advantage gradually decreases between 2nd and 6th grade as listening and reading comprehension become more interrelated. Moreover, Wolf et al. [129] found that in 1st and 2nd graders, 34 and 40% of the variance in listening and reading comprehension was explained by the other comprehension type. This suggests that comprehension taps a general comprehension process, regardless of the comprehension modality. Therefore, we expected participants to overall perform similarly on comprehension for reading and for listening during this period of development, and that the similarities would increase later in development.

Educational success is dependent on comprehension of both narrative and expository texts (e.g. [49, 130]). When directly compared, we found that children answered more narrative comprehension questions correctly than expository comprehension questions. When directly compared in the subsample of children who were later diagnosed with a speech and/or language disorder, we again found that children answered more narrative comprehension questions correctly. This evidence supports the assertation by Best et al. [11] that expository text may contribute to the “fourth-grade reading slump.” If children are still struggling to comprehend expository text by the end of fourth grade, it will be extremely difficult for them to read expository text in order to learn from it.

While comprehension has previously been shown to be associated with early expressive language development, to our knowledge no study has looked at the association between early expressive language development and expository comprehension. When investigated separately, delays in early expressive language development increased the likelihood of poorer comprehension performance for both narrative and expository comprehension. When investigated separately in the subsample of children who were later diagnosed with a speech and/or language disorder, delays in early expressive language increased the likelihood of poorer comprehension for only expository comprehension. Therefore, delays in early expressive language development do predict expository comprehension performance. Moreover, expository comprehension performance may be more tightly linked to expressive language development than narrative comprehension.

## Limitations

There is no uniformly accepted screening technique for assessing children for a speech and/or language delay. However, milestones for speech and language development are generally acknowledged as part of accepted screening techniques. Parent questionnaires and parental concerns are often used to detect early issues with speech and language development. To reach the point where an early intervention is prescribed, a specific diagnosis is often made by a speech and language specialist after administering assessments. A limitation of the current study is the lack of specificity regarding parental reports of early intervention prior to age three. Although we asked, the vast majority of the parents in our study were unable to identify the specific intervention that their child received for issues with speech or language development. Therefore, we can only suppose that the general framework of early interventions is based upon what is commonly available and administered. At this age, the administration of intervention may take place across a number of settings, including speech and language clinics, home, or preschool. The intervention itself may be provided by a clinician, caretaker, teacher, or classroom assistant. Intervention may involve only the child, a group of children together, or the child and their family. Moreover, the content of the intervention itself often depends upon the individual issues the child experiences, often expressive language but potentially alongside receptive language, phonology, syntax, and lexical acquisition. Fortunately, a small number of studies have examined the impact of speech and language intervention services administered to children under 3 years old. These studies have largely reported improvements in expressive and receptive language [44, 45, 105], listening comprehension [46], and lexical acquisition [110, 127]. Therefore, while we cannot narrow down the context or content of the early intervention children in our study received, we can assume that intervention was likely administered as a result of voiced parental concern regarding speech and language development, and that the changes corresponding to receipt of intervention services were likely to positively impact expressive language.

A second limitation of the current study is that very few parents were able to report the number of gestational weeks leading up to their child’s birth. Therefore, throughout the manuscript, the predictor variable “birth” was defined categorically as “full-term” or “premature” based on parental report, rather than a gestational cut-off. While this was not ideal, the results found here were consistent with prior studies which reported that preterm children were at greater risk for early issues with speech and language [91, 125, 135], as well as poorer comprehension outcomes (e.g., [29, 73]).

A third limitation of the current study is that we are only looking at one piece (ILF) of the reading circuitry, albeit the one most closely linked to our dependent variable: comprehension. There are a number of additional components of the reading circuitry that may be closely linked to the current framework. This paper is not meant to be all-encompassing, but rather a first attempt to link a neurobiological marker with expressive language development and comprehension. We also only included the left ILF in our analysis. It is likely the case, particularly during early development, that the right ILF plays an important role in expressive language development. Additionally, for diffusion studies, as in all neuroimaging studies, there are a number of available methods and each method requires decision points that influence the measurement outcomes (see [69] for review). Therefore, we acknowledge the possibility that a different diffusion method may reveal a less close, or potentially closer link to comprehension, and to the effect of expressive language on comprehension.

## Conclusions

These findings suggest that expressive language development impacts comprehension regardless of the modality or the genre of the passage. The left ILF was found to act as a neurodevelopmental correlate of comprehension, and to moderate the effect of expressive language development on comprehension. While in the general population sample early intervention acted as an indicator of the severity of early issues with speech and language, intervention services were found to improve comprehension in children who were later diagnosed with a speech or language disorder. Consistent with this difference, early intervention differentially moderated the effect of the left ILF on the relationship between early expressive language development and later comprehension. Left ILF FA decreased the relationship (between expressive language development and comprehension) in those who received an intervention, and increased the relationship in those who did not receive an intervention.

## Supplementary information

**Additional file 1: Appendix 1.** Supplemental information. **Table S1.** Assessment timeline by cohort. **Table S2.** Qualitative reading inventory passages–Cohort 1. **Table S3.** Experimental passages–Cohort 2. **Table S4.** Diffusion descriptive statistics for the left ILF. **Figure S1.** Interaction of socioeconomic status and fractional anisotropy of the left ILF

**Additional file 2: Appendix 2.** Manuscript Statistics

**Additional file 3: Appendix 3.** Supplemental Information Models

## Data Availability

The datasets generated and/or analyzed during the current study are not publicly available due to not all participants providing consent for their data to be shared outside of Vanderbilt University or Vanderbilt University Medical School. A subset of this data, from those participants who did provide consent for their data to be shared outside of the aforementioned institutions, are available from the corresponding author (LEC) on reasonable request.

## Abbreviations

FA: Fractional anisotropy
ILF: Inferior longitudinal fasciculus
