# Supplementary material for: The impact of expressive language development and the left inferior longitudinal fasciculus on listening and reading comprehension
Source: J Neurodev Disord. 2019 Dec 16;11:37. doi: 10.1186/s11689-019-9296-7 (PMC6912995; doi:10.1186/s11689-019-9296-7)

**Appendix 1. Supplemental Information**

**Methods**

**Parent Response Reliability.** Responses for the following questions were not expected to change over time but were asked at more than one-time point to determine parents’ internal response reliability. First, across both cohorts, a moderate degree of reliability was found between parents’ response to “What was the age at which your child learned to read?”. The intraclass correlation was 0.631 with a 95% confidence interval from 0.499 to 0.752 (*F*^44,135^ = 7.83, *p* < .001). One child’s parent did not provide a response to this question at any time point. Similarly, across both cohorts, a moderate degree of reliability was found between parents’ response to “Did your child have trouble learning how to sound-out words?”. The response was coded as yes=1 and no=0. Data was excluded from analyses when parents responded: “I don’t know”. The intraclass correlation was 0.608 with a 95% confidence interval from 0.474 to 0.732 (*F*^46,141^ = 7.19, *p* < .001). Seven children’s parents either did not provide a response to this question at any time point or responded “I don’t know” as a response at all time points.

**Comprehension Measures.** Longitudinal recurrent-event analysis could only be conducted when administration of an assessment occurs with multiple events at multiple time points. Therefore, an assessment such as the Passage Comprehension subtest from the Woodcock-Johnson (WJ: Woodcock, McGrew, Schrank, & Mather, 2007) could not be used in this study due to its administration only at one time-point (Table S1).

Of the 140 children enrolled in Cohort 1, on average 134 individuals completed the four QRI passages at Visit 1 (Completion by Passages: Mouse in the House [n=137]; The Surprise [n=133]; Air [n=136]; Brain & The Five Senses [n=131]). Of the 200 children enrolled in Cohort 2, 198 children completed both of the listening passages (Moths & The Fox and the Tiger) at Visit 1. Of the Cohort 1 children, 96 completed all four QRI passages and an additional subject completed two of the four passages (The Lucky Cricket & Whales and Fish) at Visit 2. Of the Cohort 2 children, 138 children completed the four passages at Visit 2. Of the Cohort 1 children, 93 children completed all four of the QRI passages. Of the Cohort 2 children, 52 of the 92 children in wave one completed the four passages at Visit 3.

| **Table S1: Assessment Timeline by Cohort** | | | | | |
| --- | --- | --- | --- | --- | --- |
|  | Time Point Administered | | | | |
| **Assessments** | Prescreening | Time1 | Time2 | Time3 | Time4 |
| Wechsler Abbreviated Scale of Intelligence (WASI) | -- | C1 & C2 | -- | -- | -- |
| **Reading Comprehension Measures** |  |  |  |  |  |
| Passage Comprehension (WJ) | C1 & C2 | -- | C2 | C2 | -- |
| Reading Comprehension (Gates-4) |  | C2 | C1 & C2 | C1 & C2 | C1 |
| Qualitative Reading Inventory-5 |  | C1 | C1 | C1 | C1 |
| Passages |  | C2 | C2 | C2 | -- |
| **Listening Comprehension Measures** |  |  |  |  |  |
| Listening Comprehension (WDRB) | C1 | -- | -- | -- | -- |
| Oral Comprehension (WJ-IV) | -- | C2* | C2 | C2 | -- |
| Qualitative Reading Inventory-5 | -- | C1 | C1 | C1 | C1 |
| Passages | -- | C2 | C2 | C2 | -- |
| *Notes. C1=Cohort1; C2=Cohort2; – Not administered; *Only Cohort 2 data collection wave 2 received the WJ-IV at Time1* | | | | | |

**Correlations between Comprehension Measures.** Correlations were run between measures of reading and listening comprehension and those not included as the dependent variable across survival analyses of each construct to provided additional assurance of measurement suitability. While we do not expect reading and listening comprehension standardized assessments to be identical (see Cutting & Scarborough, 2006), we do expect correlations between comprehension tests to largely show a medium-to-large correlation coefficient.

| **Table S2. Qualitative Reading Inventory Passages – Cohort 1** | | | | | | | |
| --- | --- | --- | --- | --- | --- | --- | --- |
| Visit | Indices | | | | | | |
| **Visit 1** | Total Words | Fountas & Pinnell Levels | Readability Level | Lexile | No. of Ideas | No. of Comprehension  Questions | Genre |
| Mouse in a House | 250 | H | 1.8 | -- | 44 | 6 | Narrative |
| The Surprise | 210 | I | 1.8 | -- | 44 | 6 | Narrative |
| Air | 85 | H/I | 1.5 | -- | 20 | 6 | Expository |
| Brain & Five Senses | 76 | H | 1.5 | -- | 28 | 6 | Expository |
| **Visit 2** |  |  |  |  |  |  |  |
| The Lucky Cricket | 346 | L | 2.1 | 510 | 52 | 8 | Narrative |
| Father’s New Game | 298 | M | 2.7 | 480 | 49 | 8 | Narrative |
| Whales and Fish | 197 | L | 2.9 | 590 | 49 | 8 | Expository |
| Seasons | 247 | M | 2.4 | 480 | 42 | 8 | Expository |
| **Visit 3** |  |  |  |  |  |  |  |
| The Trip to the Zoo | 312 | N | 4.3 | 650 | 55 | 8 | Narrative |
| The Friend | 357 | P | 3.9 | 710 | 55 | 8 | Narrative |
| Cats: Lions and Tigers in Your House | 261 | N | 2.7 | 750 | 47 | 8 | Expository |
| Wool: From Sheep to You | 221 | P | 4.6 | 700 | 42 | 8 | Expository |
| **Visit 4** |  |  |  |  |  |  |  |
| Johnny Appleseed | 308 | P | 4.3 | 650 | 47 | 8 | Narrative |
| Amelia Earhart | 263 | R | 3.3 | 500 | 47 | 8 | Narrative |
| The Busy Beaver | 281 | R | 3.2 | 670 | 49 | 8 | Expository |
| Plant Structures for Survival | 278 | T | 4.6 | 930 | 57 | 8 | Expository |
| *Notes. Fountas & Pinnell Levels* (Fountas, 2006)*; Readability level* (Harris & Jacobson, 1974)*; Ideas = Number of story ideas recalled; Lexile (*[*https://lexile.com/educators/tools-to-support-reading-at-school/tools-to-determine-a-books-complexity/the-lexile-analyzer/*](https://lexile.com/educators/tools-to-support-reading-at-school/tools-to-determine-a-books-complexity/the-lexile-analyzer/)*)* | | | | | | | |

| **Table S3. Experimental Passages – Cohort 2** | | | | | | | |
| --- | --- | --- | --- | --- | --- | --- | --- |
| **Visit** | **Indices** | | | | | | |
| **Visit 1** | Total Words | Flesch Reading Ease | F-K grade level | Word Concreteness | No. of Ideas | No. of Comprehension  Questions | Genre |
| Moths | 350 | 91.99 | 2.74 | 414.71 | 43 | 8 | Expository |
| The Fox and the Tiger | 350 | 92.15 | 2.59 | 428.23 | 49 | 8 | Narrative |
| **Visit 2** |  |  |  |  |  |  |  |
| The Ants and the Grasshopper | 350 | 88.53 | 3.72 | 432.39 | 48 | 8 | Narrative |
| The Monkey and the Cat | 350 | 90.19 | 3.68 | 413.66 | 45 | 8 | Narrative |
| Hot Air Balloons | 350 | 85.55 | 3.83 | 448.00 | 46 | 8 | Expository |
| Igloos | 350 | 87.95 | 3.71 | 432.54 | 44 | 8 | Expository |
| **Visit 3** |  |  |  |  |  |  |  |
| Deserts | 350 | 84.74 | 4.30 | 413.92 | 48 | 8 | Expository |
| Toads | 350 | 84.92 | 4.44 | 429.81 | 48 | 8 | Expository |
| The Farmer and the Fisherman | 350 | 84.35 | 4.41 | 442.28 | 45 | 8 | Narrative |
| The Fox and the Crow | 350 | 86.92 | 4.37 | 447.50 | 47 | 8 | Narrative |
| *Notes. Flesch Reading Ease* (Farr, Jenkins, & Paterson, 1951; Flesch, 1948)*; F-K grade level = Flesch-Kincaid Grade Level test* (Kincaid, P, Rogers, & Chissom, 1975) *is a linear function of the mean number of syllables per word and the mean number of words per sentence; Ideas = Number of story ideas recalled* | | | | | | | |

**Diffusion Imaging of the ILF.** The present study includes a total of 100 children from Cohort A and 134 children from Cohort B. At Visit 1, no differences were found in in DWI acquisition between the two Cohorts (*F* ^1, 227.5^ = 1.53, *p* = .22), nor were differences found across cohorts between the two Phillip’s scanners (*F* ^1, 162.09^ = 0.43, *p* = .51).

| **Table S4: Diffusion Descriptive Statistics for the left ILF** | | | | |
| --- | --- | --- | --- | --- |
|  | **Time Point** | | | |
|  | **Time 1**  (end of 1^st^ grade) | **Time 2**  (end of 2^nd^ grade) | **Time 3**  (end of 3^rd^ grade) | **Time 4**  (end of 4^th^ grade) |
| **N** | 234 | 181 | 67 | 50 |
| Cohort 1 | 100 | 87 | 67 | 50 |
| Cohort 2 | 134 | 94 | -- | -- |
| **FA** | 0.19 (0.01)  [0.15 – 0.22] | 0.18 (0.01)  [0.16 – 0.22] | 0.20 (0.02)  [0.17 – 0.26] | 0.19 (0.01)  [0.17 – 0.22] |
| Cohort 1 | 0.19 (0.01)  [0.15 – 0.22] | 0.19 (0.01)  [0.17 – 0.22] | 0.20 (0.02)  [0.17 – 0.26] | 0.19 (0.01)  [0.17 – 0.22] |
| Cohort 2 | 0.18 (0.01)  [0.15 – 0.22] | 0.18 (0.01)  [0.16 – 0.20] | -- | -- |
| **Volume (mm^3^)** | 39663.09 (4367.20)  [28385 – 61987] | 39854.11 (4324.62)  [29388 – 60861] | 39117 (4735.1)  [28882 – 622202] | 39887.82 (5416.84)  [30262 – 62561] |
| Cohort 1 | 39034.6 (4441.71)  [28385 – 61987] | 39363.82 (4669.75)  [29388 – 60861] | 39117 (4735.1)  [28882 – 622202] | 39887.82 (5416.84)  [30262 – 62561] |
| Cohort 2 | 40132.09 (4267.16)  [30669 – 53342] | 40307.89 (3949.66)  [32588 – 51341] | -- | -- |
| *Note. () = standard deviation of the mean; [minimum, maximum]; -- = Data not collected.* | | | | |

**Results**

**Preliminary Analyses**

***Reading Comprehension.*** For reading comprehension, the two comparison measures of comprehension were the WJ Passage Comprehension (WJ-PC) subtest Cohort 1 (WJ-III: Woodcock et al., 2007) and Cohort 2 (WJ-IV: Mather & Jaffe, 2016) and the Gates (Gates, MacGinitie, Maria, Dreyer, & Hughes, 2000). The WJ-PC subtest was completed by both cohorts (n = 327) at pre-screening (mean = 105.78, SD=13.01, Min=54, Max=136), and by Cohort 2 at Visit 2 (n = 166) & Visit 3 (n = 66).

*Pearson* correlations determined that there was, as expected, a significant positive association between the average number of correct responses on the QRI and Passage comprehension passages read by participants and the WJ passage comprehension raw score at Visit 2 (*r* = 0.61 (95% CI: 0.50, 0.70), *t*(162)=9.79, *p* < .0001) and Visit 3 (*r* = 0.40 (95% CI: 0.17, 0.59), *t*(63)=3.46, *p* < .001). The Gates was completed by both cohorts at Visit 2 (n = 235) and Visit 3 (n = 140), and by Cohort 1 at Visit 4 (n = 79) and Cohort 2 at Visit 1 (n = 197). A significant positive association was found between the average number of correct responses on the QRI and Passage comprehension passages read by the participants and the Gates comprehension raw score at Visit 2 (*r* = 0.41 (95% CI: 0.29, 0.51), *t*(224)=6.65, *p* < .0001), Visit 3 (*r* = 0.33 (95% CI: 0.17, 0.47), *t*(136)=4.08, *p* < .0001), and Visit 4 (*r* = 0.60 (95% CI: 0.44, 0.73), *t*(76)=6.57, *p* < .0001). Notably, at Visit 1 Gates correlations are not reported under reading comprehension as children in Cohort 2 only listened to passages.

***Listening Comprehension.*** For listening comprehension, the two comparison measures were the WDRB Listening Comprehension subtest (WDRB: Woodcock, 1997) and the WJ Oral Comprehension subtest (WJ-IV: Mather & Jaffe, 2016). The WDRB Listening Comprehension subtest was completed by Cohort 1 (n = 128) at pre-screening (mean = 100.94, SD=16.29, Min=53, Max=144). The WJ Oral Comprehension subtest was completed by Cohort 2 wave two at Visit 1 (n = 107), Cohort 2 at Visit 2 (n = 157), and Cohort 2 wave one at Visit 3 (n = 68). A *Pearson* correlation determined that there was a significant positive association between the average number of correct responses on the QRI and Passage comprehension passages listened to by participants and the listening score at Visit 1 (*r* = 0.41 (95% CI: 0.23, 0.55), *t*(105)=4.55, *p* < .0001), Visit 2 (*r* = 0.57 (95% CI: 0.45, 0.66), *t*(155)=8.54, *p* < .0001), and Visit 3 (*r* = 0.40 (95% CI: 0.18, 0.58), *t*(66)=3.55, *p* < .001). Therefore, both the QRI and Passage task performance was consistent with similar reading and listening comprehension tasks.

**Part 1. Does later expressive language development increase the likelihood of poorer comprehension?**

**Expressive Language Milestones.** Follow-up analyses determined if specific types of expressive language onset predicted later comprehension performance. Moving backward in time, we first asked if the expressive language onset of putting several words together predicted later comprehension performance. ***Putting Several Words Together.*** Overall model fit was significant (likelihood ratio test (9) =1352, *p* < .0001; concordance = 0.682, SE = .005). The primary results of the survival analyses suggest that children with a late language onset for putting several words together were more likely to have poor comprehension performance. Specifically, the hazard ratio for putting several words together is 1.16 (*B* = 0.15*, SE* = 0.01, z = 11.27, *p* < .001, 95% CI: 1.13 – 1.19). The likelihood of poor comprehension performance increased by 14.05% with each period of delay for putting several words together. Second, we asked if the expressive language onset of speaking one’s first word predicted later comprehension performance. ***Spoke First Word.*** Overall model fit was significant (likelihood ratio test (9) = 1275, *p* < .0001; concordance = 0.68, SE = .005). The primary results of the survival analyses suggest that children with a late language onset for the onset of speaking one’s first word were more likely to have poor comprehension performance. Specifically, the hazard ratio for putting several words together is 1.10 (*B* = 0.10*, SE* = 0.01, z = 7.08, *p* < .001, 95% CI: 1.07 – 1.13). The likelihood of poor comprehension performance increased by 9.19% with each period of delay for speaking one’s first word. Third, we asked if the expressive language onset of babbling predicted later comprehension performance. ***Babbled.*** Overall model fit was significant (likelihood ratio test (9) =1236, *p* < .0001; concordance = 0.681, SE = .005). The primary results of the survival analyses suggest that children with a late language onset for babbling would be more likely to have poor comprehension performance. Specifically, the hazard ratio for putting several words together is 1.08 (*B* = 0.08*, SE* = 0.01, z = 5.80, *p* < .001, 95% CI: 1.05 – 1.11). The likelihood of poor comprehension performance increases by 7.65% with each period of delay for beginning to babble. Therefore, moving back in time the onset of specific types of expressive language development allows us to predict later comprehension performance. Moreover, the likelihood of poor comprehension performance increased over development with each specific type of expressive language onset (i.e. babbling 7.65%, spoke first word 9.19%, and putting several words together 14.05%). Thus, delayed expressive language at later developmental markers such as putting several words together accounted were a greater risk of poor comprehension performance from the end of 1^st^ to the end of 4^th^ grade.

**Passage Feature: Modality.** A number of investigators have now found an association between expressive language development and comprehension. While the vast majority of these investigations have focused on reading comprehension, a few have found a link between expressive language development and listening comprehension. Therefore, we ran two separate models, one for only listening comprehension and another for only reading comprehension with the expectation that expressive language development would be a highly significant predictor in both analyses.

The reading model fit was significant (likelihood ratio test (8) = 524.7, *p* < .0001; concordance = 0.688, SE = .008). We found that children with delayed expressive language development had a greater likelihood of poorer reading comprehension. Specifically, the hazard ratio for expressive language is 1.21 (*B* = 0.19*, SE* = 0.02, z = 8.75, *p* < .001, 95% CI: 1.16 – 1.27). Each period of delayed expressive language development increased the risk of poorer reading comprehension by 17.59%. The listening model fit was significant (likelihood ratio test (8) = 789.4, *p* < .0001; concordance = 0.686, SE = .006). We found that children with delayed expressive language development had a greater likelihood of poor listening comprehension. Specifically, the hazard ratio for expressive language is 1.13 (*B* = 0.12*, SE* = 0.02, z = 7.43, *p* < .001, 95% CI: 1.09 – 1.17). Each period of delayed expressive language development increased the risk of poorer listening comprehension by 11.57% (see Appendix 3 for follow-up analyses that determined if specific types of expressive language onset predicted later comprehension performance by modality).

Two differences were found between the reading and listening comprehension models. In the primary analyses, we found an effect of PE tubes. This effect was not significant in the reading comprehension model (*p* = 0.18), but was significant in the listening comprehension model. The hazard ratio for PE tubes is 0.73 (*B* = -0.31*, SE* = 0.06, z = 4.77, *p* < .001, 95% CI: 0.65 – 0.83). Children with PE tubes have a 26.65% decreased likelihood of poor listening comprehension performance. Children with a frequent history of ear infections who have PE tubes inserted answered more listening comprehension questions correctly. Age, also significant in the primary analyses (see manuscript), was not significant in the listening model (*p* = 0.43) but was significant in the reading comprehension model. The hazard ratio for age is 0.82 (*B* = -0.19*, SE* = 0.07, z = 2.72, *p* < .01, 95% CI: 0.71 – 0.95). For each unit increase in age, the likelihood of poor reading comprehension performance decreased by 17.55%. Children who were older, following the completion of each grade, answered more reading comprehension questions correctly. The results of the full reading and listening comprehension models are reported in Appendix 3.

**Passage Feature: Genre.** While previous investigations have focused on the association between expressive language development and narrative listening and reading comprehension, here we aimed to investigate if the link between expressive language development and listening and reading comprehension was the same across genres. Moreover, our prior analysis found that genre did impact the likelihood of poor comprehension performance. Therefore, we ran two separate models one for only expository comprehension and another for only narrative comprehension. Overall model fit was significant (likelihood ratio test (8) = 284.9, *p* < .0001; concordance = 0.605, SE = .007). The primary results of the survival analyses suggest that children with a history of delayed expressive language development had a greater likelihood of poorer expository comprehension. Specifically, the hazard ratio for expressive language is 1.12 (*B* = 0.11*, SE* = 0.02, z = 6.82, *p* < .001, 95% CI: 1.08 – 1.16). The risk of poorer expository comprehension performance increased by 10.78% with each period of delayed expressive language development. Overall model fit was significant (likelihood ratio test (8) = 295.9, *p* < .0001; concordance = 0.629, SE = .008). The primary results of the survival analyses suggest that children with a history of delayed expressive language development had a greater likelihood of poorer narrative comprehension. Specifically, the hazard ratio for expressive language is 1.23 (*B* = 0.21*, SE* = 0.02, z = 9.71, *p* < .001, 95% CI: 1.18 – 1.28). The risk of poorer narrative comprehension performance increased by 18.66% with each period of delayed expressive language development (see Appendix 3 for follow-up analyses that determined if specific types of expressive language onset predicted later comprehension performance by genre).

Significant differences were found between the Expository and Narrative Comprehension models. Age, also significant in the primary analyses (see manuscript), was not significant in the narrative model (*p* = 0.61) but was significant in the expository comprehension model. The hazard ratio for age is 0.88 (*B* = -0.13*, SE* = 0.05, z = 2.29, *p* < .05, 95% CI: 0.79 – 0.98). For each unit increase in age, the likelihood of poor expository comprehension performance decreased by 11.78%. Children who were older, following the completion of each grade, answered more expository comprehension questions correctly. The results of the full expository and narrative comprehension models are reported in Appendix 3.

**Part 2. Does fractional anisotropy of the left ILF correspond to the likelihood of poorer comprehension?**

Follow-up analyses determined if specific types of expressive language onset drove the interaction of expressive language development and FA of the left ILF to predicted later comprehension performance. ***Putting Several Words Together.*** Overall model fit was significant (likelihood ratio test (13) = 932, *p* < .0001; concordance = 0.678, SE = .006). There was a significant interaction of FA of the left ILF and when children put several words together. The hazard ratio was 1.05 (*B* = 0.05*, SE* = 0.01, z = 3.79, *p* < .001, 95% CI: 1.03 – 1.08). For each increase in FA and putting several words together the likelihood of poor comprehension performance increased by 5.14%. ***Spoke First Word.*** Overall model fit was significant (likelihood ratio test (13) = 833.9, *p* < .0001; concordance = 0.67, SE = .006). significant interaction of FA of the left ILF and when children spoke their first word comprehension (*p* = .73). ***Babbled.*** Overall model fit was significant (likelihood ratio test (13) =809.3, *p* < .0001; concordance = 0.671, SE = .006). There was a significant interaction of FA of the left ILF and when children began babbling. The hazard ratio was 1.08 (*B* = 0.05*, SE* = 0.02, z = 3.05, *p* < .01, 95% CI: 1.04 – 1.12). For each increase in FA and babbling the likelihood of poor comprehension performance increased by 4.82%. Therefore, we find that the interaction of expressive language development and FA of the left ILF was driven by the developmental onset of babbling and putting several words together.

**Part 3. How do you decrease the likelihood of poorer comprehension?**

Follow-up independent sample t-tests revealed the children who received some type of early speech and language intervention were significantly delayed (*t*^17.87^ = 2.61, *p* < .05) in putting several words together (*M* = 2.44*, SD* = 0.92) compared to peers who did not receive early intervention (*M* = 1.87*, SD* = 0.58). Children who received intervention began putting several words together between 24-71 months old, while those who did not receive early intervention began putting several words together between 7-24 months old. A significant delay in speaking their first word (*t*^18.11^ = 2.31, *p* < .05) was also found between those who did (*M* = 1.44*, SD* = 0.78) and did not receive early intervention (*M* = 1.01*, SD* = 0.55). Children who received intervention began speaking their first word between 7-24 months old, while those who did not receive early intervention began speaking their first word from 7-12 months old. No difference in delay in babbling was found (*p* = .25) between those who did (*M* = 0.53*, SD* = 1.0) and did not receive early intervention (*M* = 0.23*, SD* = 0.47). Taken together this suggests that children who received early intervention demonstrated the most severe delays in putting two or more words together and in speaking their first word.

Children who had not received early intervention and had the highest levels of FA yielded the steepest positive slope between the developmental onset of putting several words together and the likelihood of poorer comprehension. This was also the case between the developmental onset of babbling and the likelihood of poorer comprehension, while those who had the lowest levels of FA yielded the steepest positive slope between the developmental onset of speaking one’s first word and the likelihood of poorer comprehension. Children who had received early intervention and had the lowest levels of FA yielded the steepest positive slope between putting several words together, speaking one’s first word, babbling, and the likelihood of poorer comprehension.

**References**

Cutting, L. E., & Scarborough, H. S. (2006). Prediction of reading comprehension: Relative contributions of word recognition, language proficiency, and other cognitive skills can depend on how comprehension is measured. *Scientific Studies of Reading*, *10*(3), 277–299. https://doi.org/10.1207/s1532799xssr1003_5

Farr, J. N., Jenkins, J. J., & Paterson, D. G. (1951). Simplification of Flesch reading ease formula. *Journal of Applied Psychology*, *35*(5), 333–337. https://doi.org/10.1037/h0062427

Flesch, R. (1948). A new readability yardstick. *Journal of Applied Psychology*, *32*(3), 221–233.

Fountas, I. C. (2006). The Fountas and Pinnell leveled book list K-8. Portsmouth, NH: Heinemann.

Gates, A. I., MacGinitie, R. K., Maria, K., Dreyer, L. G., & Hughes, K. E. (2000). Gates-MacGinitie Reading Tests (4th ed). Itasca, Illinois: Riverside Publishing.

Harris, A. J., & Jacobson, M. D. (1974). *Revised Harris-Jacobson readability formulas*.

Kincaid, J. P., P, F. R., Rogers, R. L., & Chissom, B. S. (1975). *Derivation of new readability formulas (automated readability index, fog count and flesch reading ease formula) for navy enlisted personnel*.

Mather, N., & Jaffe, L. E. (2016). Woocock-Johnson IV (WJ-IV). Hoboken, NJ: John Wiley & Sons.

Woodcock. (1997). Woodcock Diagnostic Reading Battery. Itasca, IL: Riverside Publishing Company.

Woodcock, R. W., McGrew, K. S., Schrank, F. A., & Mather, N. (2007). Woodcock-Johnson III Normative Update Test of Cognitive Abilities. Rolling Meadows, IL: Riverside Publishing.

**Figure S1. Interaction of socioeconomic status and fractional anisotropy of the left ILF.**

There was a significant interaction between the left ILF FA and socioeconomic status. FA functioned as a buffer between childhood socioeconomic status and the likelihood of poorer comprehension.


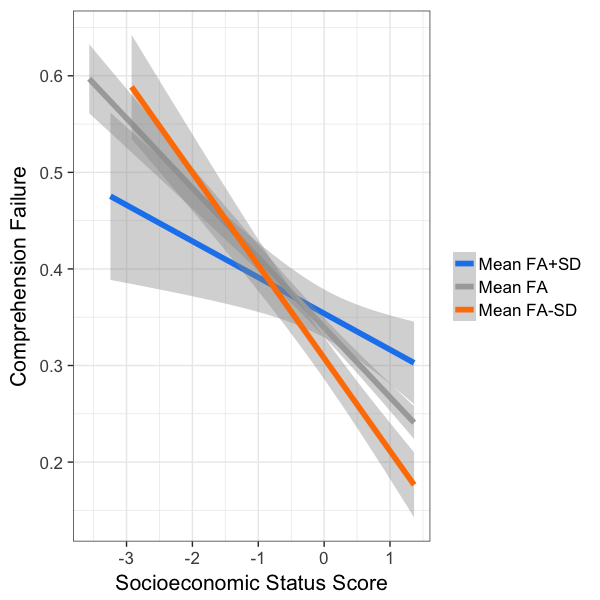

Supplement: Supplementary file 1 — Additional file 1: Appendix 1. Supplemental information. Table S1. Assessment timeline by cohort. Table S2. Qualitative reading inventory passages–Cohort 1. Table S3. Experimental passages–Cohort 2. Table S4. Diffusion descriptive statistics for the left ILF. Figure S1. Interaction of socioeconomic status and fractional anisotropy of the left ILF [file 11689_2019_9296_MOESM1_ESM.docx]
