# Supplementary material for: The impact of expressive language development and the left inferior longitudinal fasciculus on listening and reading comprehension
Source: J Neurodev Disord. 2019 Dec 16;11:37. doi: 10.1186/s11689-019-9296-7 (PMC6912995; doi:10.1186/s11689-019-9296-7)
Supplement: Supplementary file 2 — Additional file 2: Appendix 2. Manuscript Statistics [file 11689_2019_9296_MOESM2_ESM.docx]

**Appendix 2. Manuscript Statistics**

**Parent Response Reliability**

icc(data_agelearnedtoread, model="oneway", type="agreement")

Single Score Intraclass Correlation

Model: oneway

Type : agreement

Subjects = 45

Raters = 4

ICC(1) = 0.631

F-Test, H0: r0 = 0 ; H1: r0 > 0

F(44,135) = 7.83 , p = 8.58e-21

95%-Confidence Interval for ICC Population Values:

0.498 < ICC < 0.752

icc(data_soundout, model="oneway", type="agreement")

Single Score Intraclass Correlation

Model: oneway

Type : agreement

Subjects = 47

Raters = 4

ICC(1) = 0.608

F-Test, H0: r0 = 0 ; H1: r0 > 0

F(46,141) = 7.19 , p = 5.08e-20

95%-Confidence Interval for ICC Population Values:

0.474 < ICC < 0.732

**Multicollinearity Check**

#Analysis 1 Output [initial communalities (Squared Multiple Correlations) > .90]

smc(n,covar=FALSE)

babbled spokefirstword putseveralwordstogether

0.2350561 0.3792216 0.2972220

smc(n,covar=TRUE) #if covar= TRUE then return the smc * variance for each item

babbled spokefirstword putseveralwordstogether

0.06140279 0.12633066 0.11631858

**Relative Importance Metrics**

#Analysis 1 Output – Assessment of Shared Variance

library(relaimpo)

calc.relimp(test1,rela = TRUE)

test1<-lm(zputseveralwordstogether ~ zbabbled + zspokefirstword, data=testdata)

lmg

zbabbled 0.1338955

zspokefirstword 0.8661045 (86.6%)

test2<-lm(zbabbled ~ zspokefirstword + zputseveralwordstogether, data=testdata)

calc.relimp(test2,rela = TRUE)

lmg

zspokefirstword 0.7319839 (73.2%)

zputseveralwordstogether 0.2680161

**Part 1 | Survival Analyses**

#Survival Analyses Comparison

> anova(model1_rando, model1)

Analysis of Deviance Table

Cox model: response is Surv(StartTime, StopTime, Fail)

Model 1: ~ SEXcat + zSES_score + zAge + Fullterm_1yesCAT + PEtubesCAT + EarInfect_NoPECAT + Zexpressive + READ_1yesCAT + Expository_1yesCAT + frailty(SUBCAT)

Model 2: ~ SEXcat + zSES_score + zAge + Fullterm_1yesCAT + PEtubesCAT + EarInfect_NoPECAT + Zexpressive + READ_1yesCAT + Expository_1yesCAT

loglik Chisq Df P(>|Chi|)

1 -42248

2 -42614 732.98 162.94 < 2.2e-16 ***

---

Signif. codes: 0 ‘***’ 0.001 ‘**’ 0.01 ‘*’ 0.05 ‘.’ 0.1 ‘ ’ 1

#Survival Analyses (Expressive Composite)

coxph(formula = Surv(StartTime, StopTime, Fail) ~ SEXcat + zSES_score +

zAge + Fullterm_1yesCAT + PEtubesCAT + EarInfect_NoPECAT +

Zexpressive + READ_1yesCAT + Expository_1yesCAT, data = total)

n= 15469, number of events= 5326

(5547 observations deleted due to missingness)

coef exp(coef) se(coef) z Pr(>|z|)

SEXcatMALE -0.05429 0.94715 0.02816 -1.928 0.0539 .

zSES_score -0.22372 0.79954 0.01260 -17.751 < 2e-16 ***

zAge -0.09606 0.90841 0.04424 -2.171 0.0299 *

Fullterm_1yesCAT1 -0.15069 0.86012 0.03539 -4.258 2.07e-05 ***

PEtubesCAT1 -0.23368 0.79162 0.05070 -4.609 4.05e-06 ***

EarInfect_NoPECAT1 0.03829 1.03904 0.05932 0.646 0.5186

Zexpressive 0.14781 1.15929 0.01316 11.230 < 2e-16 ***

READ_1yesCAT1 -0.13497 0.87374 0.02915 -4.630 3.65e-06 ***

Expository_1yesCAT1 0.78073 2.18306 0.02885 27.064 < 2e-16 ***

---

Signif. codes: 0 ‘***’ 0.001 ‘**’ 0.01 ‘*’ 0.05 ‘.’ 0.1 ‘ ’ 1

exp(coef) exp(-coef) lower .95 upper .95

SEXcatMALE 0.9472 1.0558 0.8963 1.0009

zSES_score 0.7995 1.2507 0.7800 0.8195

zAge 0.9084 1.1008 0.8330 0.9907

Fullterm_1yesCAT1 0.8601 1.1626 0.8025 0.9219

PEtubesCAT1 0.7916 1.2632 0.7167 0.8743

EarInfect_NoPECAT1 1.0390 0.9624 0.9250 1.1671

Zexpressive 1.1593 0.8626 1.1298 1.1896

READ_1yesCAT1 0.8737 1.1445 0.8252 0.9251

Expository_1yesCAT1 2.1831 0.4581 2.0631 2.3100

Concordance= 0.685 (se = 0.005 )

Rsquare= 0.081 (max possible= 0.996 )

Likelihood ratio test= 1311 on 9 df, p=<2e-16

Wald test = 1310 on 9 df, p=<2e-16

Score (logrank) test = 1348 on 9 df, p=<2e-16

#Survival Analyses (Put Several Words Together)

coxph(formula = Surv(StartTime, StopTime, Fail) ~ SEXcat + zSES_score +

zAge + Fullterm_1yesCAT + PEtubesCAT + EarInfect_NoPECAT +

zputseveralwordstogether + READ_1yesCAT + Expository_1yesCAT,

data = total)

n= 16397, number of events= 5655

(4619 observations deleted due to missingness)

coef exp(coef) se(coef) z Pr(>|z|)

SEXcatMALE -0.01796 0.98220 0.02734 -0.657 0.51126

zSES_score -0.22112 0.80162 0.01249 -17.702 < 2e-16 ***

zAge -0.10037 0.90450 0.04325 -2.321 0.02029 *

Fullterm_1yesCAT1 -0.10734 0.89822 0.03494 -3.072 0.00213 **

PEtubesCAT1 -0.25481 0.77507 0.05049 -5.047 4.49e-07 ***

EarInfect_NoPECAT1 -0.03186 0.96864 0.05978 -0.533 0.59402

**zputseveralwordstogether 0.15138 1.16344 0.01343 11.269 < 2e-16 *****

READ_1yesCAT1 -0.13251 0.87589 0.02810 -4.716 2.41e-06 ***

Expository_1yesCAT1 0.77926 2.17986 0.02799 27.836 < 2e-16 ***

---

Signif. codes: 0 ‘***’ 0.001 ‘**’ 0.01 ‘*’ 0.05 ‘.’ 0.1 ‘ ’ 1

exp(coef) exp(-coef) lower .95 upper .95

SEXcatMALE 0.9822 1.0181 0.9309 1.0363

zSES_score 0.8016 1.2475 0.7822 0.8215

zAge 0.9045 1.1056 0.8310 0.9845

Fullterm_1yesCAT1 0.8982 1.1133 0.8388 0.9619

PEtubesCAT1 0.7751 1.2902 0.7020 0.8557

EarInfect_NoPECAT1 0.9686 1.0324 0.8615 1.0891

**zputseveralwordstogether 1.1634 0.8595 1.1332 1.1945**

READ_1yesCAT1 0.8759 1.1417 0.8290 0.9255

Expository_1yesCAT1 2.1799 0.4587 2.0635 2.3028

**Concordance= 0.682 (se = 0.005 )**

Rsquare= 0.079 (max possible= 0.996 )

**Likelihood ratio test= 1352 on 9 df, p=<2e-16**

Wald test = 1335 on 9 df, p=<2e-16

Score (logrank) test = 1377 on 9 df, p=<2e-16

#Survival Analyses (Spoke First Word)

coxph(formula = Surv(StartTime, StopTime, Fail) ~ SEXcat + zSES_score +

zAge + Fullterm_1yesCAT + PEtubesCAT + EarInfect_NoPECAT +

zspokefirstword + READ_1yesCAT + Expository_1yesCAT, data = total)

n= 16301, number of events= 5610

(4715 observations deleted due to missingness)

coef exp(coef) se(coef) z Pr(>|z|)

SEXcatMALE -0.04817 0.95297 0.02740 -1.758 0.078732 .

zSES_score -0.23004 0.79450 0.01254 -18.342 < 2e-16 ***

zAge -0.08149 0.92174 0.04369 -1.865 0.062168 .

Fullterm_1yesCAT1 -0.11853 0.88822 0.03529 -3.358 0.000784 ***

PEtubesCAT1 -0.23654 0.78935 0.05066 -4.669 3.03e-06 ***

EarInfect_NoPECAT1 0.04734 1.04848 0.05805 0.815 0.414810

**zspokefirstword 0.09643 1.10124 0.01362 7.078 1.46e-12 *****

READ_1yesCAT1 -0.12989 0.87819 0.02825 -4.598 4.26e-06 ***

Expository_1yesCAT1 0.78225 2.18639 0.02812 27.820 < 2e-16 ***

---

Signif. codes: 0 ‘***’ 0.001 ‘**’ 0.01 ‘*’ 0.05 ‘.’ 0.1 ‘ ’ 1

exp(coef) exp(-coef) lower .95 upper .95

SEXcatMALE 0.9530 1.0494 0.9031 1.0055

zSES_score 0.7945 1.2586 0.7752 0.8143

zAge 0.9217 1.0849 0.8461 1.0042

Fullterm_1yesCAT1 0.8882 1.1258 0.8289 0.9518

PEtubesCAT1 0.7894 1.2669 0.7147 0.8718

EarInfect_NoPECAT1 1.0485 0.9538 0.9357 1.1748

**zspokefirstword 1.1012 0.9081 1.0722 1.1310**

READ_1yesCAT1 0.8782 1.1387 0.8309 0.9282

Expository_1yesCAT1 2.1864 0.4574 2.0692 2.3103

**Concordance= 0.68 (se = 0.005 )**

Rsquare= 0.075 (max possible= 0.996 )

**Likelihood ratio test= 1275 on 9 df, p=<2e-16**

Wald test = 1258 on 9 df, p=<2e-16

Score (logrank) test = 1299 on 9 df, p=<2e-16

#Survival Analyses (babbled)

coxph(formula = Surv(StartTime, StopTime, Fail) ~ SEXcat + zSES_score +

zAge + Fullterm_1yesCAT + PEtubesCAT + EarInfect_NoPECAT +

zbabbled + READ_1yesCAT + Expository_1yesCAT, data = total)

n= 15661, number of events= 5392

(5355 observations deleted due to missingness)

coef exp(coef) se(coef) z Pr(>|z|)

SEXcatMALE -0.05977 0.94198 0.02790 -2.142 0.0322 *

zSES_score -0.21688 0.80503 0.01268 -17.104 < 2e-16 ***

zAge -0.04990 0.95133 0.04395 -1.135 0.2562

Fullterm_1yesCAT1 -0.20257 0.81663 0.03554 -5.699 1.20e-08 ***

PEtubesCAT1 -0.25122 0.77785 0.05065 -4.960 7.05e-07 ***

EarInfect_NoPECAT1 0.07015 1.07267 0.05955 1.178 0.2388

**zbabbled 0.07960 1.08285 0.01372 5.803 6.51e-09 *****

READ_1yesCAT1 -0.12971 0.87835 0.02897 -4.477 7.57e-06 ***

Expository_1yesCAT1 0.77915 2.17961 0.02867 27.180 < 2e-16 ***

---

Signif. codes: 0 ‘***’ 0.001 ‘**’ 0.01 ‘*’ 0.05 ‘.’ 0.1 ‘ ’ 1

exp(coef) exp(-coef) lower .95 upper .95

SEXcatMALE 0.9420 1.0616 0.8919 0.9949

zSES_score 0.8050 1.2422 0.7853 0.8253

zAge 0.9513 1.0512 0.8728 1.0369

Fullterm_1yesCAT1 0.8166 1.2245 0.7617 0.8756

PEtubesCAT1 0.7779 1.2856 0.7043 0.8590

EarInfect_NoPECAT1 1.0727 0.9323 0.9545 1.2055

**zbabbled 1.0828 0.9235 1.0541 1.1124**

READ_1yesCAT1 0.8784 1.1385 0.8299 0.9297

Expository_1yesCAT1 2.1796 0.4588 2.0605 2.3056

**Concordance= 0.681 (se = 0.005 )**

Rsquare= 0.076 (max possible= 0.996 )

**Likelihood ratio test= 1236 on 9 df, p=<2e-16**

Wald test = 1228 on 9 df, p=<2e-16

Score (logrank) test = 1265 on 9 df, p=<2e-16

**Part 2 | Survival Analyses**

#Survival Analyses (Expressive Composite)

coxph(formula = Surv(StartTime, StopTime, Fail) ~ zSES_score *

zFA + zAge + Fullterm_1yesCAT + PEtubesCAT + EarInfect_NoPECAT +

Zexpressive * zFA + SEXcat * zFA + READ_1yesCAT + Expository_1yesCAT,

data = total)

n= 10721, number of events= 3705

(10295 observations deleted due to missingness)

coef exp(coef) se(coef) z Pr(>|z|)

zSES_score -0.22499 0.79852 0.01521 -14.790 < 2e-16 ***

**zFA -0.05239 0.94895 0.02516 -2.083 0.03728 ***

zAge -0.13330 0.87521 0.05610 -2.376 0.01749 *

Fullterm_1yesCAT1 -0.09904 0.90571 0.04578 -2.163 0.03051 *

PEtubesCAT1 -0.18546 0.83072 0.06049 -3.066 0.00217 **

EarInfect_NoPECAT1 -0.00303 0.99697 0.06863 -0.044 0.96479

Zexpressive 0.15404 1.16654 0.01636 9.417 < 2e-16 ***

SEXcatMALE -0.01685 0.98330 0.03392 -0.497 0.61942

READ_1yesCAT1 -0.07389 0.92878 0.03471 -2.128 0.03330 *

Expository_1yesCAT1 0.74448 2.10534 0.03442 21.626 < 2e-16 ***

**zSES_score:zFA 0.03315 1.03370 0.01412 2.348 0.01886 ***

**zFA:Zexpressive 0.05072 1.05203 0.01553 3.266 0.00109 ****

**zFA:SEXcatMALE 0.08265 1.08616 0.03196 2.586 0.00970 ****

---

Signif. codes: 0 ‘***’ 0.001 ‘**’ 0.01 ‘*’ 0.05 ‘.’ 0.1 ‘ ’ 1

exp(coef) exp(-coef) lower .95 upper .95

zSES_score 0.7985 1.2523 0.7751 0.8227

**zFA 0.9490 1.0538 0.9033 0.9969**

zAge 0.8752 1.1426 0.7841 0.9769

Fullterm_1yesCAT1 0.9057 1.1041 0.8280 0.9907

PEtubesCAT1 0.8307 1.2038 0.7378 0.9353

EarInfect_NoPECAT1 0.9970 1.0030 0.8715 1.1405

Zexpressive 1.1665 0.8572 1.1297 1.2045

SEXcatMALE 0.9833 1.0170 0.9201 1.0509

READ_1yesCAT1 0.9288 1.0767 0.8677 0.9942

Expository_1yesCAT1 2.1053 0.4750 1.9680 2.2523

**zSES_score:zFA 1.0337 0.9674 1.0055 1.0627**

**zFA:Zexpressive 1.0520 0.9505 1.0205 1.0845**

**zFA:SEXcatMALE 1.0862 0.9207 1.0202 1.1564**

Concordance= 0.676 (se = 0.006 )

Rsquare= 0.077 (max possible= 0.995 )

Likelihood ratio test= 864.6 on 13 df, p=<2e-16

Wald test = 857.9 on 13 df, p=<2e-16

Score (logrank) test = 881.6 on 13 df, p=<2e-16

#Survival Analyses (Babbled)

coxph(formula = Surv(StartTime, StopTime, Fail) ~ zSES_score *

zFA + zAge + Fullterm_1yesCAT + PEtubesCAT + EarInfect_NoPECAT +

zbabbled * zFA + SEXcat * zFA + READ_1yesCAT + Expository_1yesCAT,

data = total)

n= 10857, number of events= 3749

(10159 observations deleted due to missingness)

coef exp(coef) se(coef) z Pr(>|z|)

zSES_score -0.22055 0.80208 0.01541 -14.309 < 2e-16 ***

zFA -0.05381 0.94761 0.02499 -2.154 0.031273 *

zAge -0.10418 0.90106 0.05585 -1.865 0.062134 .

Fullterm_1yesCAT -0.12369 0.88366 0.04629 -2.672 0.007539 **

PEtubesCAT1 -0.21541 0.80621 0.06059 -3.555 0.000378 ***

EarInfect_NoPECAT1 -0.01273 0.98735 0.06913 -0.184 0.853898

**zbabbled 0.07608 1.07905 0.01677 4.536 5.74e-06 *****

SEXcatMALE -0.01463 0.98548 0.03372 -0.434 0.664356

READ_1yesCAT1 -0.06560 0.93650 0.03453 -1.900 0.057419 .

Expository_1yesCAT1 0.74315 2.10256 0.03422 21.714 < 2e-16 ***

zSES_score:zFA 0.04180 1.04268 0.01437 2.909 0.003621 **

**zFA:zbabbled 0.04933 1.05057 0.01617 3.051 0.002284 ****

zFA:SEXcatMALE 0.09689 1.10174 0.03177 3.049 0.002294 **

---

Signif. codes: 0 ‘***’ 0.001 ‘**’ 0.01 ‘*’ 0.05 ‘.’ 0.1 ‘ ’ 1

exp(coef) exp(-coef) lower .95 upper .95

zSES_score 0.8021 1.2468 0.7782 0.8267

zFA 0.9476 1.0553 0.9023 0.9952

zAge 0.9011 1.1098 0.8076 1.0053

Fullterm_1yesCAT 0.8837 1.1317 0.8070 0.9676

PEtubesCAT1 0.8062 1.2404 0.7159 0.9079

EarInfect_NoPECAT1 0.9874 1.0128 0.8622 1.1306

**zbabbled 1.0791 0.9267 1.0442 1.1151**

SEXcatMALE 0.9855 1.0147 0.9225 1.0528

READ_1yesCAT1 0.9365 1.0678 0.8752 1.0021

Expository_1yesCAT1 2.1026 0.4756 1.9661 2.2484

zSES_score:zFA 1.0427 0.9591 1.0137 1.0725

**zFA:zbabbled 1.0506 0.9519 1.0178 1.0844**

zFA:SEXcatMALE 1.1017 0.9077 1.0352 1.1725

Concordance= 0.671 (se = 0.006 )

Rsquare= 0.072 (max possible= 0.995 )

Likelihood ratio test= 809.3 on 13 df, p=<2e-16

Wald test = 801.1 on 13 df, p=<2e-16

Score (logrank) test = 825 on 13 df, p=<2e-16

> (1-(1/1.0506))*100

[1] **4.816295**

#Survival Analyses (Spoke First Word)

coxph(formula = Surv(StartTime, StopTime, Fail) ~ zSES_score *

zFA + zAge + Fullterm_1yesCAT + PEtubesCAT + EarInfect_NoPECAT +

zspokefirstword * zFA + SEXcat * zFA + READ_1yesCAT + Expository_1yesCAT,

data = total)

n= 11145, number of events= 3845

(9871 observations deleted due to missingness)

coef exp(coef) se(coef) z Pr(>|z|)

zSES_score -0.238173 0.788067 0.015029 -15.847 < 2e-16 ***

zFA -0.041319 0.959523 0.024739 -1.670 0.09488 .

zAge -0.147007 0.863288 0.055251 -2.661 0.00780 **

Fullterm_1yesCAT -0.022492 0.977759 0.044823 -0.502 0.61581

PEtubesCAT1 -0.186284 0.830038 0.060431 -3.083 0.00205 **

EarInfect_NoPECAT1 0.011744 1.011814 0.066723 0.176 0.86028

**zspokefirstword 0.080886 1.084247 0.017509 4.620 3.85e-06 *****

SEXcatMALE -0.009100 0.990942 0.033327 -0.273 0.78482

READ_1yesCAT1 -0.074942 0.927797 0.034007 -2.204 0.02754 *

Expository_1yesCAT1 0.759740 2.137720 0.033863 22.436 < 2e-16 ***

zSES_score:zFA 0.018740 1.018916 0.013712 1.367 0.17172

**zFA:zspokefirstword -0.007271 0.992756 0.020901 -0.348 0.72795**

zFA:SEXcatMALE 0.092100 1.096475 0.032481 2.835 0.00458 **

---

Signif. codes: 0 ‘***’ 0.001 ‘**’ 0.01 ‘*’ 0.05 ‘.’ 0.1 ‘ ’ 1

exp(coef) exp(-coef) lower .95 upper .95

zSES_score 0.7881 1.2689 0.7652 0.8116

zFA 0.9595 1.0422 0.9141 1.0072

zAge 0.8633 1.1584 0.7747 0.9620

Fullterm_1yesCAT 0.9778 1.0227 0.8955 1.0675

PEtubesCAT1 0.8300 1.2048 0.7373 0.9344

EarInfect_NoPECAT1 1.0118 0.9883 0.8878 1.1532

**zspokefirstword 1.0842 0.9223 1.0477 1.1221**

SEXcatMALE 0.9909 1.0091 0.9283 1.0578

READ_1yesCAT1 0.9278 1.0778 0.8680 0.9917

Expository_1yesCAT1 2.1377 0.4678 2.0004 2.2844

zSES_score:zFA 1.0189 0.9814 0.9919 1.0467

**zFA:zspokefirstword 0.9928 1.0073 0.9529 1.0343**

zFA:SEXcatMALE 1.0965 0.9120 1.0288 1.1685

Concordance= 0.672 (se = 0.006 )

Rsquare= 0.072 (max possible= 0.995 )

Likelihood ratio test= 833.9 on 13 df, p=<2e-16

Wald test = 817.3 on 13 df, p=<2e-16

Score (logrank) test = 844 on 13 df, p=<2e-16

#Survival Analyses (Put Several Words Together)

coxph(formula = Surv(StartTime, StopTime, Fail) ~ zSES_score *

zFA + zAge + Fullterm_1yesCAT + PEtubesCAT + EarInfect_NoPECAT +

zputseveralwordstogether * zFA + SEXcat * zFA + READ_1yesCAT +

Expository_1yesCAT, data = total)

n= 11185, number of events= 3868

(9831 observations deleted due to missingness)

coef exp(coef) se(coef) z Pr(>|z|)

zSES_score -0.21462 0.80685 0.01518 -14.134 < 2e-16 ***

zFA -0.04136 0.95948 0.02474 -1.672 0.094594 .

zAge -0.16575 0.84725 0.05503 -3.012 0.002594 **

Fullterm_1yesCAT -0.02815 0.97224 0.04428 -0.636 0.524955

PEtubesCAT1 -0.17698 0.83779 0.06027 -2.936 0.003320 **

EarInfect_NoPECAT1 -0.07708 0.92581 0.06945 -1.110 0.267063

**zputseveralwordstogether 0.17529 1.19159 0.01658 10.572 < 2e-16 *****

SEXcatMALE 0.01915 1.01933 0.03336 0.574 0.565940

READ_1yesCAT1 -0.07911 0.92394 0.03385 -2.337 0.019453 *

Expository_1yesCAT1 0.75869 2.13548 0.03375 22.479 < 2e-16 ***

zSES_score:zFA 0.03846 1.03920 0.01443 2.666 0.007686 **

**zFA:zputseveralwordstogether 0.05281 1.05423 0.01395 3.786 0.000153 *****

zFA:SEXcatMALE 0.08394 1.08756 0.03120 2.690 0.007145 **

---

Signif. codes: 0 ‘***’ 0.001 ‘**’ 0.01 ‘*’ 0.05 ‘.’ 0.1 ‘ ’ 1

exp(coef) exp(-coef) lower .95 upper .95

zSES_score 0.8068 1.2394 0.7832 0.8312

zFA 0.9595 1.0422 0.9141 1.0072

zAge 0.8473 1.1803 0.7606 0.9437

Fullterm_1yesCAT 0.9722 1.0286 0.8914 1.0604

PEtubesCAT1 0.8378 1.1936 0.7444 0.9428

EarInfect_NoPECAT1 0.9258 1.0801 0.8080 1.0608

**zputseveralwordstogether 1.1916 0.8392 1.1535 1.2309**

SEXcatMALE 1.0193 0.9810 0.9548 1.0882

READ_1yesCAT1 0.9239 1.0823 0.8646 0.9873

Expository_1yesCAT1 2.1355 0.4683 1.9988 2.2815

zSES_score:zFA 1.0392 0.9623 1.0102 1.0690

**zFA:zputseveralwordstogether 1.0542 0.9486 1.0258 1.0835**

zFA:SEXcatMALE 1.0876 0.9195 1.0230 1.1562

Concordance= 0.678 (se = 0.006 )

Rsquare= 0.08 (max possible= 0.995 )

Likelihood ratio test= 932 on 13 df, p=<2e-16

Wald test = 921.8 on 13 df, p=<2e-16

Score (logrank) test = 947.7 on 13 df, p=<2e-16

> (1-(1/1.0542))*100

[1] **5.141339**

**Part 3 | Survival Analyses**

#Survival Analyses (Expressive Composite)

coxph(formula = Surv(StartTime, StopTime, Fail) ~ SEXcat * zFA +

zSES_score * zFA + zAge + Fullterm_1yesCAT + PEtubesCAT +

EarInfect_NoPECAT + Zexpressive * zFA + earlyint_CAT * zFA +

earlyint_CAT:zFA:Zexpressive + READ_1yesCAT + Expository_1yesCAT,

data = total)

n= 10329, number of events= 3547

(10687 observations deleted due to missingness)

coef exp(coef) se(coef) z Pr(>|z|)

SEXcatMALE -0.05978 0.94197 0.03565 -1.677 0.093565 .

zFA -0.05868 0.94301 0.02541 -2.310 0.020915 *

zSES_score -0.21962 0.80282 0.01590 -13.815 < 2e-16 ***

zAge -0.16591 0.84712 0.05777 -2.872 0.004077 **

Fullterm_1yesCAT -0.08857 0.91523 0.04667 -1.898 0.057713 .

PEtubesCAT1 -0.16749 0.84579 0.06083 -2.753 0.005898 **

EarInfect_NoPECAT1 0.01214 1.01222 0.06921 0.175 0.860699

Zexpressive 0.15478 1.16741 0.01715 9.025 < 2e-16 ***

**earlyint_CAT1 0.21932 1.24523 0.07388 2.968 0.002993 ****

READ_1yesCAT1 -0.09596 0.90850 0.03562 -2.694 0.007054 **

Expository_1yesCAT1 0.74600 2.10855 0.03519 21.202 < 2e-16 ***

SEXcatMALE:zFA 0.10377 1.10934 0.03328 3.118 0.001820 **

**zFA:zSES_score 0.04254 1.04345 0.01512 2.813 0.004913 ****

**zFA:Zexpressive 0.06274 1.06475 0.01615 3.885 0.000102 *****

**zFA:earlyint_CAT1 0.47417 1.60667 0.14492 3.272 0.001068 ****

**zFA:Zexpressive:earlyint_CAT1 -0.54824 0.57797 0.12112 -4.526 6e-06 *****

---

Signif. codes: 0 ‘***’ 0.001 ‘**’ 0.01 ‘*’ 0.05 ‘.’ 0.1 ‘ ’ 1

exp(coef) exp(-coef) lower .95 upper .95

SEXcatMALE 0.9420 1.0616 0.8784 1.0101

zFA 0.9430 1.0604 0.8972 0.9912

zSES_score 0.8028 1.2456 0.7782 0.8282

zAge 0.8471 1.1805 0.7564 0.9487

Fullterm_1yesCAT 0.9152 1.0926 0.8352 1.0029

PEtubesCAT1 0.8458 1.1823 0.7507 0.9529

EarInfect_NoPECAT1 1.0122 0.9879 0.8838 1.1593

Zexpressive 1.1674 0.8566 1.1288 1.2073

**earlyint_CAT1 1.2452 0.8031 1.0774 1.4393**

READ_1yesCAT1 0.9085 1.1007 0.8472 0.9742

Expository_1yesCAT1 2.1086 0.4743 1.9680 2.2591

SEXcatMALE:zFA 1.1093 0.9014 1.0393 1.1841

**zFA:zSES_score 1.0435 0.9584 1.0130 1.0748**

**zFA:Zexpressive 1.0648 0.9392 1.0316 1.0990**

**zFA:earlyint_CAT1 1.6067 0.6224 1.2094 2.1345**

**zFA:Zexpressive:earlyint_CAT1 0.5780 1.7302 0.4558 0.7328**

Concordance= 0.677 (se = 0.006 )

Rsquare= 0.08 (max possible= 0.995 )

Likelihood ratio test= 861.5 on 16 df, p=<2e-16

Wald test = 854.1 on 16 df, p=<2e-16

Score (logrank) test = 876.2 on 16 df, p=<2e-16

#Survival Analyses (Put Several Words Together)

coxph(formula = Surv(StartTime, StopTime, Fail) ~ SEXcat * zFA +

zSES_score * zFA + zAge + Fullterm_1yesCAT + PEtubesCAT +

EarInfect_NoPECAT + zputseveralwordstogether * zFA + earlyint_CAT *

zFA + earlyint_CAT:zFA:zputseveralwordstogether + READ_1yesCAT +

Expository_1yesCAT, data = total)

n= 10793, number of events= 3710

(10223 observations deleted due to missingness)

coef exp(coef) se(coef) z Pr(>|z|)

SEXcatMALE -0.02870 0.97170 0.03504 -0.819 0.412666

zFA -0.04709 0.95400 0.02499 -1.885 0.059497 .

zSES_score -0.20272 0.81650 0.01590 -12.747 < 2e-16 ***

zAge -0.17893 0.83617 0.05647 -3.169 0.001531 **

Fullterm_1yesCAT -0.01603 0.98410 0.04508 -0.356 0.722102

PEtubesCAT1 -0.15016 0.86057 0.06071 -2.474 0.013376 *

EarInfect_NoPECAT1 -0.05534 0.94617 0.07001 -0.790 0.429302

zputseveralwordstogether 0.18468 1.20283 0.01800 10.259 < 2e-16 ***

earlyint_CAT1 0.03045 1.03092 0.07734 0.394 0.693770

READ_1yesCAT1 -0.09413 0.91016 0.03468 -2.714 0.006643 **

Expository_1yesCAT1 0.76013 2.13855 0.03447 22.052 < 2e-16 ***

SEXcatMALE:zFA 0.09039 1.09460 0.03224 2.804 0.005055 **

zFA:zSES_score 0.03574 1.03638 0.01516 2.357 0.018423 *

**zFA:zputseveralwordstogether 0.05610 1.05771 0.01453 3.861 0.000113 *****

zFA:earlyint_CAT1 0.15945 1.17287 0.07666 2.080 0.037529 *

**zFA:zputseveralwordstogether:earlyint_CAT1 -0.28304 0.75349 0.09504 -2.978 0.002901 ****

---

Signif. codes: 0 ‘***’ 0.001 ‘**’ 0.01 ‘*’ 0.05 ‘.’ 0.1 ‘ ’ 1

exp(coef) exp(-coef) lower .95 upper .95

SEXcatMALE 0.9717 1.0291 0.9072 1.0408

zFA 0.9540 1.0482 0.9084 1.0019

zSES_score 0.8165 1.2247 0.7914 0.8424

zAge 0.8362 1.1959 0.7486 0.9340

Fullterm_1yesCAT 0.9841 1.0162 0.9009 1.0750

PEtubesCAT1 0.8606 1.1620 0.7640 0.9693

EarInfect_NoPECAT1 0.9462 1.0569 0.8248 1.0853

zputseveralwordstogether 1.2028 0.8314 1.1611 1.2460

earlyint_CAT1 1.0309 0.9700 0.8859 1.1997

READ_1yesCAT1 0.9102 1.0987 0.8503 0.9742

Expository_1yesCAT1 2.1386 0.4676 1.9988 2.2880

SEXcatMALE:zFA 1.0946 0.9136 1.0276 1.1660

zFA:zSES_score 1.0364 0.9649 1.0060 1.0676

**zFA:zputseveralwordstogether 1.0577 0.9454 1.0280 1.0883**

zFA:earlyint_CAT1 1.1729 0.8526 1.0092 1.3630

**zFA:zputseveralwordstogether:earlyint_CAT1 0.7535 1.3272 0.6254 0.9078**

Concordance= 0.679 (se = 0.006 )

Rsquare= 0.082 (max possible= 0.995 )

Likelihood ratio test= 921.2 on 16 df, p=<2e-16

Wald test = 913.3 on 16 df, p=<2e-16

Score (logrank) test = 937.1 on 16 df, p=<2e-16

#Survival Analyses (Spoke First Word)

coxph(formula = Surv(StartTime, StopTime, Fail) ~ SEXcat * zFA +

zSES_score * zFA + zAge + Fullterm_1yesCAT + PEtubesCAT +

EarInfect_NoPECAT + zspokefirstword * zFA + earlyint_CAT *

zFA + earlyint_CAT:zFA:zspokefirstword + READ_1yesCAT + Expository_1yesCAT,

data = total)

n= 10753, number of events= 3687

(10263 observations deleted due to missingness)

coef exp(coef) se(coef) z Pr(>|z|)

SEXcatMALE -0.054449 0.947007 0.034908 -1.560 0.11881

zFA -0.043637 0.957301 0.024970 -1.748 0.08053 .

zSES_score -0.239018 0.787400 0.015646 -15.277 < 2e-16 ***

zAge -0.176332 0.838339 0.056980 -3.095 0.00197 **

Fullterm_1yesCAT -0.010589 0.989466 0.045672 -0.232 0.81665

PEtubesCAT1 -0.168643 0.844811 0.060742 -2.776 0.00550 **

EarInfect_NoPECAT1 0.029780 1.030228 0.067320 0.442 0.65822

zspokefirstword 0.086928 1.090818 0.018438 4.715 2.42e-06 ***

earlyint_CAT1 0.329142 1.389775 0.079314 4.150 3.33e-05 ***

READ_1yesCAT1 -0.094036 0.910250 0.034855 -2.698 0.00698 **

Expository_1yesCAT1 0.761455 2.141391 0.034585 22.017 < 2e-16 ***

SEXcatMALE:zFA 0.105463 1.111225 0.033958 3.106 0.00190 **

zFA:zSES_score 0.023843 1.024130 0.014296 1.668 0.09535 .

**zFA:zspokefirstword -0.003819 0.996188 0.023099 -0.165 0.86867**

zFA:earlyint_CAT1 0.289227 1.335395 0.124066 2.331 0.01974 *

**zFA:zspokefirstword:earlyint_CAT1 -0.291655 0.747026 0.099486 -2.932 0.00337 ****

---

Signif. codes: 0 ‘***’ 0.001 ‘**’ 0.01 ‘*’ 0.05 ‘.’ 0.1 ‘ ’ 1

exp(coef) exp(-coef) lower .95 upper .95

SEXcatMALE 0.9470 1.0560 0.8844 1.0141

zFA 0.9573 1.0446 0.9116 1.0053

zSES_score 0.7874 1.2700 0.7636 0.8119

zAge 0.8383 1.1928 0.7498 0.9374

Fullterm_1yesCAT 0.9895 1.0106 0.9047 1.0821

PEtubesCAT1 0.8448 1.1837 0.7500 0.9516

EarInfect_NoPECAT1 1.0302 0.9707 0.9029 1.1755

zspokefirstword 1.0908 0.9167 1.0521 1.1310

earlyint_CAT1 1.3898 0.7195 1.1897 1.6235

READ_1yesCAT1 0.9102 1.0986 0.8501 0.9746

Expository_1yesCAT1 2.1414 0.4670 2.0010 2.2916

SEXcatMALE:zFA 1.1112 0.8999 1.0397 1.1877

zFA:zSES_score 1.0241 0.9764 0.9958 1.0532

**zFA:zspokefirstword 0.9962 1.0038 0.9521 1.0423**

zFA:earlyint_CAT1 1.3354 0.7488 1.0471 1.7030

**zFA:zspokefirstword:earlyint_CAT1 0.7470 1.3386 0.6147 0.9079**

Concordance= 0.673 (se = 0.006 )

Rsquare= 0.073 (max possible= 0.995 )

Likelihood ratio test= 819.4 on 16 df, p=<2e-16

Wald test = 805 on 16 df, p=<2e-16

Score (logrank) test = 831 on 16 df, p=<2e-16

#Survival Analyses (Babbled)

coxph(formula = Surv(StartTime, StopTime, Fail) ~ SEXcat * zFA +

zSES_score * zFA + zAge + Fullterm_1yesCAT + PEtubesCAT +

EarInfect_NoPECAT + zbabbled * zFA + earlyint_CAT * zFA +

earlyint_CAT:zFA:zbabbled + READ_1yesCAT + Expository_1yesCAT,

data = total)

n= 10465, number of events= 3591

(10551 observations deleted due to missingness)

coef exp(coef) se(coef) z Pr(>|z|)

SEXcatMALE -0.04692 0.95416 0.03528 -1.330 0.183501

zFA -0.05425 0.94720 0.02516 -2.156 0.031055 *

zSES_score -0.22206 0.80087 0.01608 -13.811 < 2e-16 ***

zAge -0.13007 0.87803 0.05777 -2.252 0.024343 *

Fullterm_1yesCAT -0.10898 0.89675 0.04714 -2.312 0.020798 *

PEtubesCAT1 -0.20654 0.81340 0.06105 -3.383 0.000717 ***

EarInfect_NoPECAT1 -0.01429 0.98581 0.06980 -0.205 0.837801

zbabbled 0.06674 1.06902 0.01752 3.809 0.000140 ***

earlyint_CAT1 0.27736 1.31964 0.07841 3.537 0.000404 ***

READ_1yesCAT1 -0.08484 0.91866 0.03542 -2.395 0.016605 *

Expository_1yesCAT1 0.74402 2.10438 0.03497 21.276 < 2e-16 ***

SEXcatMALE:zFA 0.10639 1.11226 0.03274 3.249 0.001156 **

zFA:zSES_score 0.05344 1.05490 0.01529 3.496 0.000473 ***

**zFA:zbabbled 0.06265 1.06465 0.01735 3.611 0.000304 *****

zFA:earlyint_CAT1 0.17158 1.18718 0.10791 1.590 0.111811

**zFA:zbabbled:earlyint_CAT1 -0.24211 0.78497 0.08352 -2.899 0.003745 ****

---

Signif. codes: 0 ‘***’ 0.001 ‘**’ 0.01 ‘*’ 0.05 ‘.’ 0.1 ‘ ’ 1

exp(coef) exp(-coef) lower .95 upper .95

SEXcatMALE 0.9542 1.0480 0.8904 1.0225

zFA 0.9472 1.0557 0.9016 0.9951

zSES_score 0.8009 1.2486 0.7760 0.8265

zAge 0.8780 1.1389 0.7840 0.9833

Fullterm_1yesCAT 0.8968 1.1151 0.8176 0.9836

PEtubesCAT1 0.8134 1.2294 0.7217 0.9168

EarInfect_NoPECAT1 0.9858 1.0144 0.8598 1.1303

zbabbled 1.0690 0.9354 1.0329 1.1064

earlyint_CAT1 1.3196 0.7578 1.1317 1.5388

READ_1yesCAT1 0.9187 1.0885 0.8570 0.9847

Expository_1yesCAT1 2.1044 0.4752 1.9650 2.2537

SEXcatMALE:zFA 1.1123 0.8991 1.0431 1.1860

zFA:zSES_score 1.0549 0.9480 1.0238 1.0870

**zFA:zbabbled 1.0647 0.9393 1.0291 1.1015**

zFA:earlyint_CAT1 1.1872 0.8423 0.9609 1.4668

**zFA:zbabbled:earlyint_CAT1 0.7850 1.2739 0.6664 0.9246**

Concordance= 0.672 (se = 0.006 )

Rsquare= 0.072 (max possible= 0.995 )

Likelihood ratio test= 785.3 on 16 df, p=<2e-16

Wald test = 777 on 16 df, p=<2e-16

Score (logrank) test = 800 on 16 df, p=<2e-16

**Part 4 | Survival Analyses**

#Survival Analyses for only those with later speech language issues (Expressive Composite)

Call:

coxph(formula = Surv(StartTime, StopTime, Fail) ~ zSES_score +

Zexpressive + earlyint_CAT + Expository_1yesCAT + SEXcat:zFA,

data = SLdata)

n= 1264, number of events= 469

(1186 observations deleted due to missingness)

coef exp(coef) se(coef) z Pr(>|z|)

**zSES_score -0.19340 0.82415 0.02914 -6.637 3.21e-11 *****

**Zexpressive 0.28412 1.32859 0.04655 6.103 1.04e-09 *****

**earlyint_CAT1 -0.49412 0.61011 0.17939 -2.754 0.00588 ****

**Expository_1yesCAT1 0.85719 2.35652 0.09817 8.732 < 2e-16 *****

**SEXcatFEMALE:zFA -0.09917 0.90559 0.05326 -1.862 0.06260 .**

SEXcatMALE:zFA 0.01491 1.01502 0.08536 0.175 0.86131

---

Signif. codes: 0 ‘***’ 0.001 ‘**’ 0.01 ‘*’ 0.05 ‘.’ 0.1 ‘ ’ 1

exp(coef) exp(-coef) lower .95 upper .95

zSES_score 0.8241 1.2134 0.7784 0.8726

Zexpressive 1.3286 0.7527 1.2127 1.4555

earlyint_CAT1 0.6101 1.6391 0.4292 0.8672

Expository_1yesCAT1 2.3565 0.4244 1.9441 2.8565

SEXcatFEMALE:zFA 0.9056 1.1043 0.8158 1.0052

SEXcatMALE:zFA 1.0150 0.9852 0.8587 1.1999

Concordance= 0.749 (se = 0.015 )

Rsquare= 0.146 (max possible= 0.984 )

Likelihood ratio test= 199.6 on 6 df, p=<2e-16

Wald test = 202.6 on 6 df, p=<2e-16

Score (logrank) test = 216.4 on 6 df, p=<2e-16

#Survival Analyses for only those with later speech language issues (Babbled)

coxph(formula = Surv(StartTime, StopTime, Fail) ~ zSES_score +

zbabbled + earlyint_CAT + Expository_1yesCAT + SEXcat:zFA,

data = SLdata)

n= 1264, number of events= 469

(1186 observations deleted due to missingness)

coef exp(coef) se(coef) z Pr(>|z|)

zSES_score -0.217680 0.804383 0.032145 -6.772 1.27e-11 ***

**zbabbled 0.098333 1.103330 0.050931 1.931 0.053518 .**

earlyint_CAT1 -0.344681 0.708446 0.183634 -1.877 0.060519 .

Expository_1yesCAT1 0.844833 2.327589 0.098139 8.608 < 2e-16 ***

SEXcatFEMALE:zFA -0.176415 0.838270 0.052589 -3.355 0.000795 ***

SEXcatMALE:zFA -0.004478 0.995532 0.089634 -0.050 0.960157

---

Signif. codes: 0 ‘***’ 0.001 ‘**’ 0.01 ‘*’ 0.05 ‘.’ 0.1 ‘ ’ 1

exp(coef) exp(-coef) lower .95 upper .95

zSES_score 0.8044 1.2432 0.7553 0.8567

**zbabbled 1.1033 0.9063 0.9985 1.2192**

earlyint_CAT1 0.7084 1.4115 0.4943 1.0154

Expository_1yesCAT1 2.3276 0.4296 1.9203 2.8213

SEXcatFEMALE:zFA 0.8383 1.1929 0.7562 0.9293

SEXcatMALE:zFA 0.9955 1.0045 0.8351 1.1867

Concordance= 0.722 (se = 0.016 )

Rsquare= 0.125 (max possible= 0.984 )

Likelihood ratio test= 169 on 6 df, p=<2e-16

Wald test = 170.6 on 6 df, p=<2e-16

Score (logrank) test = 180.6 on 6 df, p=<2e-16

#Survival Analyses for only those with later speech language issues (Spoke First Word)

coxph(formula = Surv(StartTime, StopTime, Fail) ~ zSES_score +

zspokefirstword + earlyint_CAT + Expository_1yesCAT + SEXcat:zFA,

data = SLdata)

n= 1384, number of events= 507

(1066 observations deleted due to missingness)

coef exp(coef) se(coef) z Pr(>|z|)

zSES_score -0.23572 0.79000 0.02742 -8.597 < 2e-16 ***

**zspokefirstword 0.20595 1.22870 0.03999 5.151 2.6e-07 *****

earlyint_CAT1 -0.53686 0.58458 0.18394 -2.919 0.00352 **

Expository_1yesCAT1 0.87725 2.40428 0.09475 9.259 < 2e-16 ***

SEXcatFEMALE:zFA -0.11063 0.89527 0.05274 -2.097 0.03595 *

SEXcatMALE:zFA 0.03067 1.03114 0.08318 0.369 0.71235

---

Signif. codes: 0 ‘***’ 0.001 ‘**’ 0.01 ‘*’ 0.05 ‘.’ 0.1 ‘ ’ 1

exp(coef) exp(-coef) lower .95 upper .95

zSES_score 0.7900 1.2658 0.7487 0.8336

**zspokefirstword 1.2287 0.8139 1.1361 1.3289**

earlyint_CAT1 0.5846 1.7106 0.4076 0.8383

Expository_1yesCAT1 2.4043 0.4159 1.9968 2.8949

SEXcatFEMALE:zFA 0.8953 1.1170 0.8074 0.9928

SEXcatMALE:zFA 1.0311 0.9698 0.8760 1.2137

Concordance= 0.731 (se = 0.015 )

Rsquare= 0.139 (max possible= 0.984 )

Likelihood ratio test= 207.4 on 6 df, p=<2e-16

Wald test = 210.6 on 6 df, p=<2e-16

Score (logrank) test = 224.5 on 6 df, p=<2e-16

#Survival Analyses for only those with later speech language issues (Put Several Words Together)

coxph(formula = Surv(StartTime, StopTime, Fail) ~ zSES_score +

zputseveralwordstogether + earlyint_CAT + Expository_1yesCAT +

SEXcat:zFA, data = SLdata)

n= 1384, number of events= 507

(1066 observations deleted due to missingness)

coef exp(coef) se(coef) z Pr(>|z|)

zSES_score -0.20200 0.81709 0.02916 -6.928 4.26e-12 ***

**zputseveralwordstogether 0.38046 1.46295 0.05593 6.803 1.03e-11 *****

earlyint_CAT1 -0.21838 0.80382 0.17992 -1.214 0.2248

Expository_1yesCAT1 0.87135 2.39013 0.09472 9.200 < 2e-16 ***

SEXcatFEMALE:zFA -0.05796 0.94368 0.05591 -1.037 0.2998

SEXcatMALE:zFA 0.18344 1.20135 0.08300 2.210 0.0271 *

---

Signif. codes: 0 ‘***’ 0.001 ‘**’ 0.01 ‘*’ 0.05 ‘.’ 0.1 ‘ ’ 1

exp(coef) exp(-coef) lower .95 upper .95

zSES_score 0.8171 1.2239 0.7717 0.8651

**zputseveralwordstogether 1.4630 0.6835 1.3111 1.6324**

earlyint_CAT1 0.8038 1.2441 0.5650 1.1437

Expository_1yesCAT1 2.3901 0.4184 1.9852 2.8777

SEXcatFEMALE:zFA 0.9437 1.0597 0.8457 1.0530

SEXcatMALE:zFA 1.2013 0.8324 1.0210 1.4136

Concordance= 0.749 (se = 0.014 )

Rsquare= 0.15 (max possible= 0.984 )

Likelihood ratio test= 224.7 on 6 df, p=<2e-16

Wald test = 233 on 6 df, p=<2e-16

Score (logrank) test = 249.9 on 6 df, p=<2e-16

#Survival Analyses (Expository) for only those with later speech language issues (Expressive Composite)

coxph(formula = Surv(StartTime, StopTime, Fail) ~ zSES_score +

Zexpressive + earlyint_CAT + SEXcat:zFA, data = SLdata_EXP)

n= 640, number of events= 312

(592 observations deleted due to missingness)

coef exp(coef) se(coef) z Pr(>|z|)

zSES_score -0.16076 0.85150 0.03679 -4.370 1.24e-05 ***

Zexpressive 0.29841 1.34771 0.05739 5.200 2.00e-07 ***

earlyint_CAT1 -0.53681 0.58461 0.21558 -2.490 0.0128 *

SEXcatFEMALE:zFA -0.07594 0.92687 0.06587 -1.153 0.2490

SEXcatMALE:zFA 0.13764 1.14756 0.09943 1.384 0.1663

---

Signif. codes: 0 ‘***’ 0.001 ‘**’ 0.01 ‘*’ 0.05 ‘.’ 0.1 ‘ ’ 1

exp(coef) exp(-coef) lower .95 upper .95

zSES_score 0.8515 1.1744 0.7923 0.9152

Zexpressive 1.3477 0.7420 1.2043 1.5082

earlyint_CAT1 0.5846 1.7105 0.3832 0.8920

SEXcatFEMALE:zFA 0.9269 1.0789 0.8146 1.0546

SEXcatMALE:zFA 1.1476 0.8714 0.9444 1.3945

Concordance= 0.693 (se = 0.022 )

Rsquare= 0.1 (max possible= 0.991 )

Likelihood ratio test= 67.38 on 5 df, p=4e-13

Wald test = 73.25 on 5 df, p=2e-14

Score (logrank) test = 77.83 on 5 df, p=2e-15

#Survival Analyses (Narrative) for only those with later speech language issues (Expressive Composite)

coxph(formula = Surv(StartTime, StopTime, Fail) ~ zSES_score +

Zexpressive + earlyint_CAT + SEXcat:zFA, data = SLdata_NAR)

n= 624, number of events= 157

(594 observations deleted due to missingness)

coef exp(coef) se(coef) z Pr(>|z|)

zSES_score -0.24176 0.78524 0.04884 -4.950 7.41e-07 ***

Zexpressive 0.28060 1.32393 0.08060 3.481 0.000499 ***

earlyint_CAT1 -0.51678 0.59644 0.34178 -1.512 0.130528

SEXcatFEMALE:zFA -0.13098 0.87723 0.09293 -1.409 0.158693

SEXcatMALE:zFA -0.33437 0.71579 0.16172 -2.068 0.038678 *

---

Signif. codes: 0 ‘***’ 0.001 ‘**’ 0.01 ‘*’ 0.05 ‘.’ 0.1 ‘ ’ 1

exp(coef) exp(-coef) lower .95 upper .95

zSES_score 0.7852 1.2735 0.7136 0.8641

Zexpressive 1.3239 0.7553 1.1305 1.5505

earlyint_CAT1 0.5964 1.6766 0.3052 1.1654

SEXcatFEMALE:zFA 0.8772 1.1399 0.7312 1.0525

SEXcatMALE:zFA 0.7158 1.3971 0.5213 0.9827

Concordance= 0.71 (se = 0.026 )

Rsquare= 0.105 (max possible= 0.915 )

Likelihood ratio test= 68.9 on 5 df, p=2e-13

Wald test = 71.19 on 5 df, p=6e-14

Score (logrank) test = 79.63 on 5 df, p=1e-15

#Survival Analyses (Expository) for only those with later speech language issues (Babbled)

coxph(formula = Surv(StartTime, StopTime, Fail) ~ zSES_score +

zbabbled + earlyint_CAT + SEXcat * zFA, data = SLdata_EXP)

n= 640, number of events= 312

(592 observations deleted due to missingness)

coef exp(coef) se(coef) z Pr(>|z|)

zSES_score -0.17577 0.83881 0.04276 -4.111 3.94e-05 ***

**zbabbled 0.14379 1.15464 0.06784 2.119 0.0341 ***

earlyint_CAT1 -0.31974 0.72634 0.31639 -1.011 0.3122

SEXcatMALE -0.08523 0.91830 0.18267 -0.467 0.6408

zFA -0.15263 0.85845 0.06478 -2.356 0.0185 *

SEXcatMALE:zFA 0.22916 1.25754 0.12388 1.850 0.0643 .

---

Signif. codes: 0 ‘***’ 0.001 ‘**’ 0.01 ‘*’ 0.05 ‘.’ 0.1 ‘ ’ 1

exp(coef) exp(-coef) lower .95 upper .95

zSES_score 0.8388 1.1922 0.7714 0.9121

**zbabbled 1.1546 0.8661 1.0109 1.3188**

earlyint_CAT1 0.7263 1.3768 0.3907 1.3504

SEXcatMALE 0.9183 1.0890 0.6419 1.3136

zFA 0.8584 1.1649 0.7561 0.9747

SEXcatMALE:zFA 1.2575 0.7952 0.9865 1.6031

Concordance= 0.676 (se = 0.023 )

Rsquare= 0.074 (max possible= 0.991 )

Likelihood ratio test= 48.99 on 6 df, p=7e-09

Wald test = 54.17 on 6 df, p=7e-10

Score (logrank) test = 56.42 on 6 df, p=2e-10

#Survival Analyses (Expository) for only those with later speech language issues (Spoke First Word)

coxph(formula = Surv(StartTime, StopTime, Fail) ~ zSES_score +

zspokefirstword + earlyint_CAT + SEXcat * zFA, data = SLdata_EXP)

n= 700, number of events= 340

(532 observations deleted due to missingness)

coef exp(coef) se(coef) z Pr(>|z|)

zSES_score -0.21005 0.81054 0.03504 -5.995 2.04e-09 ***

**zspokefirstword 0.19433 1.21449 0.05062 3.839 0.000124 *****

earlyint_CAT1 -0.44510 0.64076 0.30708 -1.449 0.147212

SEXcatMALE -0.10294 0.90218 0.17299 -0.595 0.551790

zFA -0.09539 0.90902 0.06502 -1.467 0.142341

SEXcatMALE:zFA 0.22528 1.25268 0.12095 1.863 0.062509 .

---

Signif. codes: 0 ‘***’ 0.001 ‘**’ 0.01 ‘*’ 0.05 ‘.’ 0.1 ‘ ’ 1

exp(coef) exp(-coef) lower .95 upper .95

zSES_score 0.8105 1.2337 0.7567 0.8682

**zspokefirstword 1.2145 0.8234 1.0998 1.3412**

earlyint_CAT1 0.6408 1.5606 0.3510 1.1697

SEXcatMALE 0.9022 1.1084 0.6427 1.2663

zFA 0.9090 1.1001 0.8003 1.0326

SEXcatMALE:zFA 1.2527 0.7983 0.9883 1.5878

Concordance= 0.656 (se = 0.022 )

Rsquare= 0.084 (max possible= 0.991 )

Likelihood ratio test= 61.6 on 6 df, p=2e-11

Wald test = 67.34 on 6 df, p=1e-12

Score (logrank) test = 70.39 on 6 df, p=3e-13

#Survival Analyses (Expository) for only those with later speech language issues (Put Several Words Together)

coxph(formula = Surv(StartTime, StopTime, Fail) ~ zSES_score +

zputseveralwordstogether + earlyint_CAT + SEXcat * zFA, data = SLdata_EXP)

n= 700, number of events= 340

(532 observations deleted due to missingness)

coef exp(coef) se(coef) z Pr(>|z|)

zSES_score -0.18764 0.82891 0.03673 -5.109 3.24e-07 ***

**zputseveralwordstogether 0.35624 1.42796 0.06852 5.199 2.00e-07 *****

earlyint_CAT1 0.09580 1.10054 0.29386 0.326 0.7444

SEXcatMALE -0.30788 0.73501 0.17298 -1.780 0.0751 .

zFA -0.05121 0.95008 0.06780 -0.755 0.4501

SEXcatMALE:zFA 0.25370 1.28879 0.12271 2.068 0.0387 *

---

Signif. codes: 0 ‘***’ 0.001 ‘**’ 0.01 ‘*’ 0.05 ‘.’ 0.1 ‘ ’ 1

exp(coef) exp(-coef) lower .95 upper .95

zSES_score 0.8289 1.2064 0.7713 0.8908

**zputseveralwordstogether 1.4280 0.7003 1.2485 1.6332**

earlyint_CAT1 1.1005 0.9086 0.6187 1.9577

SEXcatMALE 0.7350 1.3605 0.5237 1.0316

zFA 0.9501 1.0525 0.8319 1.0851

SEXcatMALE:zFA 1.2888 0.7759 1.0133 1.6392

Concordance= 0.7 (se = 0.021 )

Rsquare= 0.098 (max possible= 0.991 )

Likelihood ratio test= 72.23 on 6 df, p=1e-13

Wald test = 79.71 on 6 df, p=4e-15

Score (logrank) test = 83.66 on 6 df, p=6e-1

#Survival Analyses (Narrative) for only those with later speech language issues (Babbled)

coxph(formula = Surv(StartTime, StopTime, Fail) ~ zSES_score +

zbabbled + earlyint_CAT + SEXcat * zFA, data = SLdata_NAR)

n= 624, number of events= 157

(594 observations deleted due to missingness)

coef exp(coef) se(coef) z Pr(>|z|)

zSES_score -0.28253 0.75388 0.05655 -4.996 5.86e-07 ***

zbabbled 0.03786 1.03859 0.09735 0.389 0.6973

earlyint_CAT1 -0.37400 0.68797 0.50999 -0.733 0.4633

SEXcatMALE 0.04067 1.04151 0.28866 0.141 0.8880

zFA -0.21765 0.80441 0.09167 -2.374 0.0176 *

SEXcatMALE:zFA -0.04892 0.95225 0.23368 -0.209 0.8342

---

Signif. codes: 0 ‘***’ 0.001 ‘**’ 0.01 ‘*’ 0.05 ‘.’ 0.1 ‘ ’ 1

exp(coef) exp(-coef) lower .95 upper .95

zSES_score 0.7539 1.3265 0.6748 0.8422

zbabbled 1.0386 0.9628 0.8582 1.2569

earlyint_CAT1 0.6880 1.4535 0.2532 1.8693

SEXcatMALE 1.0415 0.9601 0.5915 1.8339

zFA 0.8044 1.2431 0.6721 0.9627

SEXcatMALE:zFA 0.9523 1.0501 0.6023 1.5054

Concordance= 0.678 (se = 0.028 )

Rsquare= 0.088 (max possible= 0.915 )

Likelihood ratio test= 57.8 on 6 df, p=1e-10

Wald test = 60.38 on 6 df, p=4e-11

Score (logrank) test = 67.57 on 6 df, p=1e-12

#Survival Analyses (Narrative) for only those with later speech language issues (Spoke First Word)

coxph(formula = Surv(StartTime, StopTime, Fail) ~ zSES_score +

zspokefirstword + earlyint_CAT + SEXcat * zFA, data = SLdata_NAR)

n= 684, number of events= 167

(534 observations deleted due to missingness)

coef exp(coef) se(coef) z Pr(>|z|)

zSES_score -0.27880 0.75669 0.04690 -5.944 2.78e-09 ***

**zspokefirstword 0.23868 1.26957 0.07079 3.372 0.000747 *****

earlyint_CAT1 -0.80751 0.44597 0.48060 -1.680 0.092919 .

SEXcatMALE 0.19242 1.21218 0.26753 0.719 0.471973

zFA -0.13120 0.87704 0.09303 -1.410 0.158451

SEXcatMALE:zFA -0.09206 0.91205 0.22267 -0.413 0.679287

---

Signif. codes: 0 ‘***’ 0.001 ‘**’ 0.01 ‘*’ 0.05 ‘.’ 0.1 ‘ ’ 1

exp(coef) exp(-coef) lower .95 upper .95

zSES_score 0.7567 1.3215 0.6902 0.8296

**zspokefirstword 1.2696 0.7877 1.1051 1.4585**

earlyint_CAT1 0.4460 2.2423 0.1739 1.1439

SEXcatMALE 1.2122 0.8250 0.7175 2.0478

zFA 0.8770 1.1402 0.7309 1.0525

SEXcatMALE:zFA 0.9120 1.0964 0.5895 1.4111

Concordance= 0.679 (se = 0.027 )

Rsquare= 0.099 (max possible= 0.914 )

Likelihood ratio test= 71.65 on 6 df, p=2e-13

Wald test = 76.36 on 6 df, p=2e-14

Score (logrank) test = 85.85 on 6 df, p=<2e-16

#Survival Analyses (Narrative) for only those with later speech language issues (Put Several Words Together)

coxph(formula = Surv(StartTime, StopTime, Fail) ~ zSES_score +

zputseveralwordstogether + earlyint_CAT + SEXcat * zFA, data = SLdata_NAR)

n= 684, number of events= 167

(534 observations deleted due to missingness)

coef exp(coef) se(coef) z Pr(>|z|)

zSES_score -0.25023 0.77862 0.04997 -5.008 5.51e-07 ***

**zputseveralwordstogether 0.42547 1.53031 0.09411 4.521 6.16e-06 *****

earlyint_CAT1 -0.12542 0.88213 0.47027 -0.267 0.790

SEXcatMALE -0.05695 0.94464 0.26997 -0.211 0.833

zFA -0.08139 0.92184 0.09666 -0.842 0.400

SEXcatMALE:zFA -0.02621 0.97413 0.23153 -0.113 0.910

---

Signif. codes: 0 ‘***’ 0.001 ‘**’ 0.01 ‘*’ 0.05 ‘.’ 0.1 ‘ ’ 1

exp(coef) exp(-coef) lower .95 upper .95

zSES_score 0.7786 1.2843 0.7060 0.8587

**zputseveralwordstogether 1.5303 0.6535 1.2725 1.8403**

earlyint_CAT1 0.8821 1.1336 0.3509 2.2173

SEXcatMALE 0.9446 1.0586 0.5565 1.6035

zFA 0.9218 1.0848 0.7627 1.1141

SEXcatMALE:zFA 0.9741 1.0266 0.6188 1.5336

Concordance= 0.713 (se = 0.026 )

Rsquare= 0.11 (max possible= 0.914 )

Likelihood ratio test= 79.36 on 6 df, p=5e-15

Wald test = 88.39 on 6 df, p=<2e-16

Score (logrank) test = 101 on 6 df, p=<2e-16

Score (logrank) test = 282.4 on 8 df, p=<2e-16
