# Supplementary material for: The impact of expressive language development and the left inferior longitudinal fasciculus on listening and reading comprehension
Source: J Neurodev Disord. 2019 Dec 16;11:37. doi: 10.1186/s11689-019-9296-7 (PMC6912995; doi:10.1186/s11689-019-9296-7)
Supplement: Supplementary file 3 — Additional file 3: Appendix 3. Supplemental Information Models [file 11689_2019_9296_MOESM3_ESM.docx]

**Appendix 3. Supplemental Information Models**

**Survival Analysis S1**

###SURVIVAL MODALITY MODELS ####

#Survival Analyses (Reading)

coxph(formula = Surv(StartTime, StopTime, Fail) ~ SEXcat + zSES_score +

zAge + Premature_0yesCAT + EarInfect_NoPECAT + PEtubesCAT +

Zexpressive + Expository_1yesCAT, data = data_Read)

n= 6248, number of events= 2048

(2189 observations deleted due to missingness)

coef exp(coef) se(coef) z Pr(>|z|)

SEXcatMALE -0.01787 0.98229 0.04576 -0.391 0.69616

zSES_score -0.26143 0.76995 0.01983 -13.181 < 2e-16 ***

**zAge -0.19292 0.82455 0.07368 -2.619 0.00883 ****

Premature_0yesCAT1 -0.11839 0.88835 0.05540 -2.137 0.03258 *

EarInfect_NoPECAT1 0.03894 1.03970 0.09309 0.418 0.67577

PEtubesCAT1 -0.10889 0.89683 0.08124 -1.340 0.18015

Zexpressive 0.19344 1.21341 0.02211 8.747 < 2e-16 ***

**Expository_1yesCAT1 0.72430 2.06329 0.04624 15.664 < 2e-16 *****

---

Signif. codes: 0 ‘***’ 0.001 ‘**’ 0.01 ‘*’ 0.05 ‘.’ 0.1 ‘ ’ 1

exp(coef) exp(-coef) lower .95 upper .95

SEXcatMALE 0.9823 1.0180 0.8980 1.0745

zSES_score 0.7699 1.2988 0.7406 0.8005

**zAge 0.8245 1.2128 0.7137 0.9526**

Premature_0yesCAT1 0.8883 1.1257 0.7969 0.9902

EarInfect_NoPECAT1 1.0397 0.9618 0.8663 1.2478

PEtubesCAT1 0.8968 1.1150 0.7648 1.0516

Zexpressive 1.2134 0.8241 1.1619 1.2672

**Expository_1yesCAT1 2.0633 0.4847 1.8845 2.2590**

Concordance= 0.688 (se = 0.008 )

Rsquare= 0.081 (max possible= 0.991 )

Likelihood ratio test= 524.7 on 8 df, p=<2e-16

Wald test = 536.3 on 8 df, p=<2e-16

Score (logrank) test = 547.9 on 8 df, p=<2e-16

#Survival Analyses (Reading) predicted by babbling

Model_Reading <- coxph(Surv(StartTime, StopTime, Fail) ~ SEXcat+ zSES_score + zAge + Premature_0yesCAT + EarInfect_NoPECAT + PEtubesCAT + zbabbled + Expository_1yesCAT, data=data_reading)

summary(Model_Reading)

coxph(formula = Surv(StartTime, StopTime, Fail) ~ SEXcat + zSES_score +

zAge + Premature_0yesCAT + EarInfect_NoPECAT + PEtubesCAT +

zbabbled + Expository_1yesCAT, data = data_reading)

n= 6336, number of events= 2077

(2101 observations deleted due to missingness)

coef exp(coef) se(coef) z Pr(>|z|)

SEXcatMALE -0.03874 0.96200 0.04511 -0.859 0.390488

zSES_score -0.24706 0.78110 0.02002 -12.340 < 2e-16 ***

zAge -0.13423 0.87439 0.07332 -1.831 0.067137 .

Premature_0yesCAT1 -0.19388 0.82376 0.05594 -3.466 0.000528 ***

EarInfect_NoPECAT1 0.09972 1.10486 0.09400 1.061 0.288754

PEtubesCAT1 -0.13123 0.87701 0.08117 -1.617 0.105949

**zbabbled 0.11320 1.11985 0.02346 4.824 1.4e-06 *****

Expository_1yesCAT1 0.72085 2.05619 0.04589 15.707 < 2e-16 ***

---

Signif. codes: 0 ‘***’ 0.001 ‘**’ 0.01 ‘*’ 0.05 ‘.’ 0.1 ‘ ’ 1

exp(coef) exp(-coef) lower .95 upper .95

SEXcatMALE 0.9620 1.0395 0.8806 1.0509

zSES_score 0.7811 1.2803 0.7510 0.8124

zAge 0.8744 1.1437 0.7574 1.0095

Premature_0yesCAT1 0.8238 1.2140 0.7382 0.9192

EarInfect_NoPECAT1 1.1049 0.9051 0.9190 1.3284

PEtubesCAT1 0.8770 1.1402 0.7480 1.0283

**zbabbled 1.1199 0.8930 1.0695 1.1726**

Expository_1yesCAT1 2.0562 0.4863 1.8793 2.2497

Concordance= 0.682 (se = 0.008 )

Rsquare= 0.072 (max possible= 0.991 )

Likelihood ratio test= 474.3 on 8 df, p=<2e-16

Wald test = 482 on 8 df, p=<2e-16

Score (logrank) test = 492.9 on 8 df, p=<2e-16

#Survival Analyses(Reading) predicted by spoke first word

Model_Reading <- coxph(Surv(StartTime, StopTime, Fail) ~ SEXcat+ zSES_score + zAge + Premature_0yesCAT + EarInfect_NoPECAT + PEtubesCAT + zspokefirstword + Expository_1yesCAT, data=data_reading)

summary(Model_Reading)

coxph(formula = Surv(StartTime, StopTime, Fail) ~ SEXcat + zSES_score +

zAge + Premature_0yesCAT + EarInfect_NoPECAT + PEtubesCAT +

zspokefirstword + Expository_1yesCAT, data = data_reading)

n= 6656, number of events= 2188

(1781 observations deleted due to missingness)

coef exp(coef) se(coef) z Pr(>|z|)

SEXcatMALE -0.01508 0.98503 0.04402 -0.343 0.7320

zSES_score -0.26443 0.76764 0.01974 -13.395 < 2e-16 ***

zAge -0.17774 0.83716 0.07253 -2.451 0.0143 *

Premature_0yesCAT1 -0.06345 0.93852 0.05529 -1.148 0.2511

EarInfect_NoPECAT1 0.08629 1.09012 0.09056 0.953 0.3407

PEtubesCAT1 -0.10977 0.89604 0.08116 -1.353 0.1762

**zspokefirstword 0.12976 1.13856 0.02206 5.883 4.02e-09 *****

Expository_1yesCAT1 0.71650 2.04726 0.04470 16.028 < 2e-16 ***

---

Signif. codes: 0 ‘***’ 0.001 ‘**’ 0.01 ‘*’ 0.05 ‘.’ 0.1 ‘ ’ 1

exp(coef) exp(-coef) lower .95 upper .95

SEXcatMALE 0.9850 1.0152 0.9036 1.0738

zSES_score 0.7676 1.3027 0.7385 0.7979

zAge 0.8372 1.1945 0.7262 0.9650

Premature_0yesCAT1 0.9385 1.0655 0.8421 1.0459

EarInfect_NoPECAT1 1.0901 0.9173 0.9128 1.3018

PEtubesCAT1 0.8960 1.1160 0.7643 1.0505

**zspokefirstword 1.1386 0.8783 1.0904 1.1889**

Expository_1yesCAT1 2.0473 0.4885 1.8755 2.2347

Concordance= 0.68 (se = 0.008 )

Rsquare= 0.07 (max possible= 0.992 )

Likelihood ratio test= 484.6 on 8 df, p=<2e-16

Wald test = 486.7 on 8 df, p=<2e-16

Score (logrank) test = 499 on 8 df, p=<2e-16

#Survival Analyses(Reading) predicted by put several words together

coxph(formula = Surv(StartTime, StopTime, Fail) ~ SEXcat + zSES_score +

zAge + Premature_0yesCAT + EarInfect_NoPECAT + PEtubesCAT +

zputseveralwordstogether + Expository_1yesCAT, data = data_reading)

n= 6712, number of events= 2209

(1725 observations deleted due to missingness)

coef exp(coef) se(coef) z Pr(>|z|)

SEXcatMALE 0.009078 1.009120 0.043864 0.207 0.83604

zSES_score -0.254629 0.775204 0.019671 -12.944 < 2e-16 ***

zAge -0.190822 0.826279 0.071471 -2.670 0.00759 **

Premature_0yesCAT1 -0.066902 0.935287 0.054434 -1.229 0.21905

EarInfect_NoPECAT1 -0.044780 0.956208 0.094008 -0.476 0.63383

PEtubesCAT1 -0.129083 0.878901 0.080916 -1.595 0.11065

**zputseveralwordstogether 0.168016 1.182955 0.021193 7.928 2.23e-15 *****

Expository_1yesCAT1 0.711439 2.036920 0.044471 15.998 < 2e-16 ***

---

Signif. codes: 0 ‘***’ 0.001 ‘**’ 0.01 ‘*’ 0.05 ‘.’ 0.1 ‘ ’ 1

exp(coef) exp(-coef) lower .95 upper .95

SEXcatMALE 1.0091 0.9910 0.9260 1.0997

zSES_score 0.7752 1.2900 0.7459 0.8057

zAge 0.8263 1.2102 0.7183 0.9505

Premature_0yesCAT1 0.9353 1.0692 0.8406 1.0406

EarInfect_NoPECAT1 0.9562 1.0458 0.7953 1.1497

PEtubesCAT1 0.8789 1.1378 0.7500 1.0299

**zputseveralwordstogether 1.1830 0.8453 1.1348 1.2331**

Expository_1yesCAT1 2.0369 0.4909 1.8669 2.2224

Concordance= 0.677 (se = 0.008 )

Rsquare= 0.073 (max possible= 0.992 )

Likelihood ratio test= 511.3 on 8 df, p=<2e-16

Wald test = 513.2 on 8 df, p=<2e-16

Score (logrank) test = 525.9 on 8 df, p=<2e-16

#Survival Analyses (Listening)

coxph(formula = Surv(StartTime, StopTime, Fail) ~ SEXcat + zSES_score +

zAge + Premature_0yesCAT + EarInfect_NoPECAT + PEtubesCAT +

Zexpressive + Expository_1yesCAT, data = data_Listen)

n= 9221, number of events= 3278

(2594 observations deleted due to missingness)

coef exp(coef) se(coef) z Pr(>|z|)

SEXcatMALE -0.06793 0.93433 0.03585 -1.895 0.058153 .

zSES_score -0.19663 0.82149 0.01646 -11.944 < 2e-16 ***

zAge -0.04353 0.95740 0.05543 -0.785 0.432178

Premature_0yesCAT1 -0.17700 0.83778 0.04618 -3.833 0.000127 ***

EarInfect_NoPECAT1 0.03589 1.03655 0.07714 0.465 0.641723

**PEtubesCAT1 -0.30998 0.73346 0.06495 -4.773 1.82e-06 *****

Zexpressive 0.12293 1.13080 0.01655 7.427 1.11e-13 ***

**Expository_1yesCAT1 0.81683 2.26331 0.03692 22.123 < 2e-16 *****

---

Signif. codes: 0 ‘***’ 0.001 ‘**’ 0.01 ‘*’ 0.05 ‘.’ 0.1 ‘ ’ 1

exp(coef) exp(-coef) lower .95 upper .95

SEXcatMALE 0.9343 1.0703 0.8709 1.0023

zSES_score 0.8215 1.2173 0.7954 0.8484

zAge 0.9574 1.0445 0.8588 1.0673

Premature_0yesCAT1 0.8378 1.1936 0.7653 0.9172

EarInfect_NoPECAT1 1.0365 0.9647 0.8911 1.2057

**PEtubesCAT1 0.7335 1.3634 0.6458 0.8330**

Zexpressive 1.1308 0.8843 1.0947 1.1681

**Expository_1yesCAT1 2.2633 0.4418 2.1053 2.4332**

Concordance= 0.686 (se = 0.006 )

Rsquare= 0.082 (max possible= 0.996 )

Likelihood ratio test= 789.4 on 8 df, p=<2e-16

Wald test = 774.7 on 8 df, p=<2e-16

Score (logrank) test = 802.5 on 8 df, p=<2e-16

#Survival Analyses (Listening) predicted by Babbling

Model_Listening <- coxph(Surv(StartTime, StopTime, Fail) ~ SEXcat+ zSES_score + zAge + Premature_0yesCAT + EarInfect_NoPECAT + PEtubesCAT + zbabbled + Expository_1yesCAT, data=data_listening)

summary(Model_Listening)

coxph(formula = Surv(StartTime, StopTime, Fail) ~ SEXcat + zSES_score +

zAge + Premature_0yesCAT + EarInfect_NoPECAT + PEtubesCAT +

zbabbled + Expository_1yesCAT, data = data_listening)

n= 9325, number of events= 3315

(2490 observations deleted due to missingness)

coef exp(coef) se(coef) z Pr(>|z|)

SEXcatMALE -0.066907 0.935282 0.035580 -1.880 0.060043 .

zSES_score -0.193145 0.824362 0.016561 -11.663 < 2e-16 ***

zAge -0.006276 0.993743 0.055051 -0.114 0.909228

Premature_0yesCAT1 -0.216523 0.805314 0.046253 -4.681 2.85e-06 ***

EarInfect_NoPECAT1 0.056663 1.058299 0.077301 0.733 0.463543

PEtubesCAT1 -0.324890 0.722607 0.064885 -5.007 5.52e-07 ***

**zbabbled 0.061547 1.063481 0.017081 3.603 0.000314 *****

Expository_1yesCAT1 0.816975 2.263643 0.036719 22.250 < 2e-16 ***

---

Signif. codes: 0 ‘***’ 0.001 ‘**’ 0.01 ‘*’ 0.05 ‘.’ 0.1 ‘ ’ 1

exp(coef) exp(-coef) lower .95 upper .95

SEXcatMALE 0.9353 1.0692 0.8723 1.0028

zSES_score 0.8244 1.2131 0.7980 0.8516

zAge 0.9937 1.0063 0.8921 1.1070

Premature_0yesCAT1 0.8053 1.2418 0.7355 0.8817

EarInfect_NoPECAT1 1.0583 0.9449 0.9095 1.2314

PEtubesCAT1 0.7226 1.3839 0.6363 0.8206

**zbabbled 1.0635 0.9403 1.0285 1.0997**

Expository_1yesCAT1 2.2636 0.4418 2.1065 2.4326

Concordance= 0.684 (se = 0.006 )

Rsquare= 0.078 (max possible= 0.996 )

Likelihood ratio test= 759.4 on 8 df, p=<2e-16

Wald test = 741.9 on 8 df, p=<2e-16

Score (logrank) test = 769.9 on 8 df, p=<2e-16

#Survival Analyses (Listening) predicted by Spoke First Word

Model_Listening <- coxph(Surv(StartTime, StopTime, Fail) ~ SEXcat+ zSES_score + zAge + Premature_0yesCAT + EarInfect_NoPECAT + PEtubesCAT + zspokefirstword + Expository_1yesCAT, data=data_listening)

summary(Model_Listening)

coxph(formula = Surv(StartTime, StopTime, Fail) ~ SEXcat + zSES_score +

zAge + Premature_0yesCAT + EarInfect_NoPECAT + PEtubesCAT +

zspokefirstword + Expository_1yesCAT, data = data_listening)

n= 9645, number of events= 3422

(2170 observations deleted due to missingness)

coef exp(coef) se(coef) z Pr(>|z|)

SEXcatMALE -0.06170 0.94017 0.03510 -1.758 0.078792 .

zSES_score -0.20600 0.81384 0.01640 -12.564 < 2e-16 ***

zAge -0.02833 0.97207 0.05483 -0.517 0.605388

Premature_0yesCAT1 -0.15927 0.85276 0.04598 -3.464 0.000533 ***

EarInfect_NoPECAT1 0.02203 1.02227 0.07580 0.291 0.771357

PEtubesCAT1 -0.31290 0.73132 0.06491 -4.820 1.43e-06 ***

**zspokefirstword 0.07676 1.07979 0.01737 4.420 9.89e-06 *****

Expository_1yesCAT1 0.82522 2.28238 0.03618 22.808 < 2e-16 ***

---

Signif. codes: 0 ‘***’ 0.001 ‘**’ 0.01 ‘*’ 0.05 ‘.’ 0.1 ‘ ’ 1

exp(coef) exp(-coef) lower .95 upper .95

SEXcatMALE 0.9402 1.0636 0.8777 1.0071

zSES_score 0.8138 1.2287 0.7881 0.8404

zAge 0.9721 1.0287 0.8730 1.0824

Premature_0yesCAT1 0.8528 1.1727 0.7793 0.9332

EarInfect_NoPECAT1 1.0223 0.9782 0.8811 1.1860

PEtubesCAT1 0.7313 1.3674 0.6440 0.8305

**zspokefirstword 1.0798 0.9261 1.0436 1.1172**

Expository_1yesCAT1 2.2824 0.4381 2.1261 2.4501

Concordance= 0.684 (se = 0.006 )

Rsquare= 0.079 (max possible= 0.996 )

Likelihood ratio test= 792.1 on 8 df, p=<2e-16

Wald test = 770.5 on 8 df, p=<2e-16

Score (logrank) test = 801.3 on 8 df, p=<2e-16

#Survival Analyses (Listening) predicted by Put Several Words Together

Model_Listening <- coxph(Surv(StartTime, StopTime, Fail) ~ SEXcat+ zSES_score + zAge + Premature_0yesCAT + EarInfect_NoPECAT + PEtubesCAT + zputseveralwordstogether + Expository_1yesCAT, data=data_listening)

summary(Model_Listening)

coxph(formula = Surv(StartTime, StopTime, Fail) ~ SEXcat + zSES_score +

zAge + Premature_0yesCAT + EarInfect_NoPECAT + PEtubesCAT +

zputseveralwordstogether + Expository_1yesCAT, data = data_listening)

n= 9685, number of events= 3446

(2130 observations deleted due to missingness)

coef exp(coef) se(coef) z Pr(>|z|)

SEXcatMALE -0.03089 0.96959 0.03501 -0.882 0.37766

zSES_score -0.19784 0.82050 0.01631 -12.129 < 2e-16 ***

zAge -0.04978 0.95144 0.05441 -0.915 0.36024

Premature_0yesCAT1 -0.14030 0.86909 0.04565 -3.073 0.00212 **

EarInfect_NoPECAT1 -0.02609 0.97425 0.07766 -0.336 0.73690

PEtubesCAT1 -0.33005 0.71888 0.06468 -5.103 3.34e-07 ***

**zputseveralwordstogether 0.14124 1.15170 0.01738 8.126 4.44e-16 *****

Expository_1yesCAT1 0.82363 2.27877 0.03604 22.852 < 2e-16 ***

---

Signif. codes: 0 ‘***’ 0.001 ‘**’ 0.01 ‘*’ 0.05 ‘.’ 0.1 ‘ ’ 1

exp(coef) exp(-coef) lower .95 upper .95

SEXcatMALE 0.9696 1.0314 0.9053 1.0385

zSES_score 0.8205 1.2188 0.7947 0.8472

zAge 0.9514 1.0510 0.8552 1.0585

Premature_0yesCAT1 0.8691 1.1506 0.7947 0.9504

EarInfect_NoPECAT1 0.9742 1.0264 0.8367 1.1344

PEtubesCAT1 0.7189 1.3910 0.6333 0.8160

**zputseveralwordstogether 1.1517 0.8683 1.1131 1.1916**

Expository_1yesCAT1 2.2788 0.4388 2.1233 2.4456

Concordance= 0.686 (se = 0.006 )

Rsquare= 0.083 (max possible= 0.996 )

Likelihood ratio test= 839.2 on 8 df, p=<2e-16

Wald test = 817.9 on 8 df, p=<2e-16

Score (logrank) test = 848.9 on 8 df, p=<2e-16

###SURVIVAL GENRE MODELS ####

#Survival Analyses (Expository)

coxph(formula = Surv(StartTime, StopTime, Fail) ~ SEXcat + zSES_score +

zAge + Premature_0yesCAT + EarInfect_NoPECAT + PEtubesCAT +

Zexpressive + READ_1yesCAT, data = data_Expository)

n= 7748, number of events= 3484

(2766 observations deleted due to missingness)

coef exp(coef) se(coef) z Pr(>|z|)

**SEXcatMALE -0.07966 0.92343 0.03487 -2.284 0.022352 ***

zSES_score -0.19520 0.82267 0.01586 -12.309 < 2e-16 ***

**zAge -0.12535 0.88219 0.05466 -2.293 0.021840 ***

Premature_0yesCAT1 -0.16100 0.85129 0.04429 -3.635 0.000278 ***

EarInfect_NoPECAT1 0.07880 1.08198 0.07294 1.080 0.280010

PEtubesCAT1 -0.25382 0.77583 0.06247 -4.063 4.85e-05 ***

**Zexpressive 0.11407 1.12083 0.01672 6.822 8.96e-12 *****

READ_1yesCAT1 -0.14872 0.86181 0.03617 -4.112 3.92e-05 ***

---

Signif. codes: 0 ‘***’ 0.001 ‘**’ 0.01 ‘*’ 0.05 ‘.’ 0.1 ‘ ’ 1

exp(coef) exp(-coef) lower .95 upper .95

**SEXcatMALE 0.9234 1.0829 0.8624 0.9888**

zSES_score 0.8227 1.2156 0.7975 0.8486

**zAge 0.8822 1.1335 0.7926 0.9820**

Premature_0yesCAT1 0.8513 1.1747 0.7805 0.9285

EarInfect_NoPECAT1 1.0820 0.9242 0.9379 1.2483

PEtubesCAT1 0.7758 1.2889 0.6864 0.8769

Zexpressive 1.1208 0.8922 1.0847 1.1582

READ_1yesCAT1 0.8618 1.1604 0.8028 0.9251

Concordance= 0.605 (se = 0.007 )

Rsquare= 0.036 (max possible= 0.999 )

Likelihood ratio test= 284.9 on 8 df, p=<2e-16

Wald test = 301.6 on 8 df, p=<2e-16

Score (logrank) test = 301.8 on 8 df, p=<2e-16

#Survival Analyses (Expository) predicted by babbling

Model_Expository <- coxph(Surv(StartTime, StopTime, Fail) ~ SEXcat+ zSES_score + zAge + Premature_0yesCAT + EarInfect_NoPECAT + PEtubesCAT + zbabbled + READ_1yesCAT, data=data_expository)

summary(Model_Expository)

coxph(formula = Surv(StartTime, StopTime, Fail) ~ SEXcat + zSES_score +

zAge + Premature_0yesCAT + EarInfect_NoPECAT + PEtubesCAT +

zbabbled + READ_1yesCAT, data = data_expository)

n= 7844, number of events= 3526

(2670 observations deleted due to missingness)

coef exp(coef) se(coef) z Pr(>|z|)

SEXcatMALE -0.07896 0.92407 0.03456 -2.285 0.0223 *

zSES_score -0.18690 0.82953 0.01593 -11.734 < 2e-16 ***

zAge -0.09215 0.91197 0.05418 -1.701 0.0890 .

Premature_0yesCAT1 -0.20685 0.81314 0.04446 -4.653 3.27e-06 ***

EarInfect_NoPECAT1 0.11287 1.11948 0.07318 1.542 0.1230

PEtubesCAT1 -0.26329 0.76852 0.06242 -4.218 2.46e-05 ***

**zbabbled 0.07945 1.08270 0.01701 4.671 3.00e-06 *****

READ_1yesCAT1 -0.14562 0.86449 0.03595 -4.050 5.12e-05 ***

---

Signif. codes: 0 ‘***’ 0.001 ‘**’ 0.01 ‘*’ 0.05 ‘.’ 0.1 ‘ ’ 1

exp(coef) exp(-coef) lower .95 upper .95

SEXcatMALE 0.9241 1.0822 0.8636 0.9888

zSES_score 0.8295 1.2055 0.8040 0.8558

zAge 0.9120 1.0965 0.8201 1.0141

Premature_0yesCAT1 0.8131 1.2298 0.7453 0.8872

EarInfect_NoPECAT1 1.1195 0.8933 0.9699 1.2921

PEtubesCAT1 0.7685 1.3012 0.6800 0.8685

**zbabbled 1.0827 0.9236 1.0472 1.1194**

READ_1yesCAT1 0.8645 1.1568 0.8057 0.9276

Concordance= 0.604 (se = 0.007 )

Rsquare= 0.033 (max possible= 0.999 )

Likelihood ratio test= 261.4 on 8 df, p=<2e-16

Wald test = 276.6 on 8 df, p=<2e-16

Score (logrank) test = 276.9 on 8 df, p=<2e-16

#Survival Analyses (Expository) predicted by spoke first word

Model_Expository <- coxph(Surv(StartTime, StopTime, Fail) ~ SEXcat+ zSES_score + zAge + Premature_0yesCAT + EarInfect_NoPECAT + PEtubesCAT + zspokefirstword + READ_1yesCAT, data=data_expository)

summary(Model_Expository)

coxph(formula = Surv(StartTime, StopTime, Fail) ~ SEXcat + zSES_score +

zAge + Premature_0yesCAT + EarInfect_NoPECAT + PEtubesCAT +

zspokefirstword + READ_1yesCAT, data = data_expository)

n= 8164, number of events= 3673

(2350 observations deleted due to missingness)

coef exp(coef) se(coef) z Pr(>|z|)

SEXcatMALE -0.06752 0.93471 0.03390 -1.991 0.046428 *

zSES_score -0.19803 0.82035 0.01576 -12.569 < 2e-16 ***

zAge -0.12033 0.88663 0.05384 -2.235 0.025412 *

Premature_0yesCAT1 -0.14295 0.86680 0.04405 -3.245 0.001175 **

EarInfect_NoPECAT1 0.09188 1.09623 0.07108 1.293 0.196127

PEtubesCAT1 -0.26009 0.77098 0.06242 -4.167 3.09e-05 ***

**zspokefirstword 0.06489 1.06704 0.01697 3.823 0.000132 *****

READ_1yesCAT1 -0.14709 0.86321 0.03506 -4.196 2.72e-05 ***

---

Signif. codes: 0 ‘***’ 0.001 ‘**’ 0.01 ‘*’ 0.05 ‘.’ 0.1 ‘ ’ 1

exp(coef) exp(-coef) lower .95 upper .95

SEXcatMALE 0.9347 1.0698 0.8746 0.9989

zSES_score 0.8203 1.2190 0.7954 0.8461

zAge 0.8866 1.1279 0.7978 0.9853

Premature_0yesCAT1 0.8668 1.1537 0.7951 0.9450

EarInfect_NoPECAT1 1.0962 0.9122 0.9537 1.2601

PEtubesCAT1 0.7710 1.2971 0.6822 0.8713

**zspokefirstword 1.0670 0.9372 1.0321 1.1031**

READ_1yesCAT1 0.8632 1.1585 0.8059 0.9246

Concordance= 0.598 (se = 0.007 )

Rsquare= 0.03 (max possible= 0.999 )

Likelihood ratio test= 248.9 on 8 df, p=<2e-16

Wald test = 260.3 on 8 df, p=<2e-16

Score (logrank) test = 261.3 on 8 df, p=<2e-16

#Survival Analyses (Expository) predicted by put several words together

Model_Expository <- coxph(Surv(StartTime, StopTime, Fail) ~ SEXcat+ zSES_score + zAge + Premature_0yesCAT + EarInfect_NoPECAT + PEtubesCAT + zputseveralwordstogether + READ_1yesCAT, data=data_expository)

summary(Model_Expository)

coxph(formula = Surv(StartTime, StopTime, Fail) ~ SEXcat + zSES_score +

zAge + Premature_0yesCAT + EarInfect_NoPECAT + PEtubesCAT +

zputseveralwordstogether + READ_1yesCAT, data = data_expository)

n= 8212, number of events= 3699

(2302 observations deleted due to missingness)

coef exp(coef) se(coef) z Pr(>|z|)

SEXcatMALE -0.04537 0.95565 0.03386 -1.340 0.18028

zSES_score -0.19105 0.82609 0.01569 -12.180 < 2e-16 ***

zAge -0.12854 0.87938 0.05346 -2.404 0.01620 *

Premature_0yesCAT1 -0.13353 0.87500 0.04369 -3.056 0.00224 **

EarInfect_NoPECAT1 0.03561 1.03625 0.07337 0.485 0.62739

PEtubesCAT1 -0.27589 0.75889 0.06222 -4.434 9.24e-06 ***

**zputseveralwordstogether 0.10314 1.10865 0.01683 6.130 8.79e-10 *****

READ_1yesCAT1 -0.15099 0.85986 0.03489 -4.328 1.51e-05 ***

---

Signif. codes: 0 ‘***’ 0.001 ‘**’ 0.01 ‘*’ 0.05 ‘.’ 0.1 ‘ ’ 1

exp(coef) exp(-coef) lower .95 upper .95

SEXcatMALE 0.9556 1.046 0.8943 1.0212

zSES_score 0.8261 1.211 0.8011 0.8519

zAge 0.8794 1.137 0.7919 0.9765

Premature_0yesCAT1 0.8750 1.143 0.8032 0.9532

EarInfect_NoPECAT1 1.0363 0.965 0.8975 1.1965

PEtubesCAT1 0.7589 1.318 0.6718 0.8573

**zputseveralwordstogether 1.1086 0.902 1.0727 1.1458**

READ_1yesCAT1 0.8599 1.163 0.8030 0.9207

Concordance= 0.6 (se = 0.007 )

Rsquare= 0.032 (max possible= 0.999 )

Likelihood ratio test= 270.1 on 8 df, p=<2e-16

Wald test = 281.5 on 8 df, p=<2e-16

#Survival Analyses (Narrative)

coxph(formula = Surv(StartTime, StopTime, Fail) ~ SEXcat + zSES_score +

zAge + Premature_0yesCAT + EarInfect_NoPECAT + PEtubesCAT +

Zexpressive + READ_1yesCAT, data = data_Narrative)

n= 7721, number of events= 1842

(2781 observations deleted due to missingness)

coef exp(coef) se(coef) z Pr(>|z|)

SEXcatMALE -0.0005857 0.9994145 0.0478021 -0.012 0.9902

zSES_score -0.2743485 0.7600672 0.0207597 -13.215 <2e-16 ***

zAge -0.0383435 0.9623823 0.0752369 -0.510 0.6103

Premature_0yesCAT1 -0.1436421 0.8661977 0.0589246 -2.438 0.0148 *

EarInfect_NoPECAT1 -0.0272321 0.9731353 0.1019076 -0.267 0.7893

PEtubesCAT1 -0.1952194 0.8226541 0.0867852 -2.249 0.0245 *

**Zexpressive 0.2066212 1.2295167 0.0212796 9.710 <2e-16 *****

READ_1yesCAT1 -0.1098707 0.8959500 0.0492795 -2.230 0.0258 *

---

Signif. codes: 0 ‘***’ 0.001 ‘**’ 0.01 ‘*’ 0.05 ‘.’ 0.1 ‘ ’ 1

exp(coef) exp(-coef) lower .95 upper .95

SEXcatMALE 0.9994 1.0006 0.9100 1.0976

zSES_score 0.7601 1.3157 0.7298 0.7916

zAge 0.9624 1.0391 0.8304 1.1153

Premature_0yesCAT1 0.8662 1.1545 0.7717 0.9722

EarInfect_NoPECAT1 0.9731 1.0276 0.7969 1.1883

PEtubesCAT1 0.8227 1.2156 0.6940 0.9752

Zexpressive 1.2295 0.8133 1.1793 1.2819

READ_1yesCAT1 0.8959 1.1161 0.8135 0.9868

Concordance= 0.629 (se = 0.008 )

Rsquare= 0.038 (max possible= 0.971 )

Likelihood ratio test= 295.9 on 8 df, p=<2e-16

Wald test = 326.1 on 8 df, p=<2e-16

Score (logrank) test = 329 on 8 df, p=<2e-16

#Survival Analyses (Narrative) predicted by babbling

Model_Narrative <- coxph(Surv(StartTime, StopTime, Fail) ~ SEXcat+ zSES_score + zAge + Premature_0yesCAT + EarInfect_NoPECAT + PEtubesCAT + zbabbled + READ_1yesCAT, data=data_narrative)

summary(Model_Narrative)

coxph(formula = Surv(StartTime, StopTime, Fail) ~ SEXcat + zSES_score +

zAge + Premature_0yesCAT + EarInfect_NoPECAT + PEtubesCAT +

zbabbled + READ_1yesCAT, data = data_narrative)

n= 7817, number of events= 1866

(2685 observations deleted due to missingness)

coef exp(coef) se(coef) z Pr(>|z|)

SEXcatMALE -0.019831 0.980364 0.047304 -0.419 0.675044

zSES_score -0.271217 0.762451 0.021017 -12.905 < 2e-16 ***

zAge 0.032556 1.033092 0.075135 0.433 0.664793

Premature_0yesCAT1 -0.203030 0.816253 0.059262 -3.426 0.000613 ***

EarInfect_NoPECAT1 -0.008221 0.991812 0.102462 -0.080 0.936048

PEtubesCAT1 -0.228825 0.795468 0.086684 -2.640 0.008297 **

**zbabbled 0.078425 1.081582 0.023267 3.371 0.000750 *****

READ_1yesCAT1 -0.101465 0.903513 0.048947 -2.073 0.038176 *

---

Signif. codes: 0 ‘***’ 0.001 ‘**’ 0.01 ‘*’ 0.05 ‘.’ 0.1 ‘ ’ 1

exp(coef) exp(-coef) lower .95 upper .95

SEXcatMALE 0.9804 1.0200 0.8936 1.0756

zSES_score 0.7625 1.3116 0.7317 0.7945

zAge 1.0331 0.9680 0.8916 1.1970

Premature_0yesCAT1 0.8163 1.2251 0.7267 0.9168

EarInfect_NoPECAT1 0.9918 1.0083 0.8114 1.2124

PEtubesCAT1 0.7955 1.2571 0.6712 0.9428

**zbabbled 1.0816 0.9246 1.0334 1.1320**

READ_1yesCAT1 0.9035 1.1068 0.8209 0.9945

Concordance= 0.619 (se = 0.008 )

Rsquare= 0.028 (max possible= 0.971 )

Likelihood ratio test= 224.4 on 8 df, p=<2e-16

Wald test = 246.7 on 8 df, p=<2e-16

Score (logrank) test = 248 on 8 df, p=<2e-16

#Survival Analyses (Narrative) predicted by spoke first word

Model_Narrative <- coxph(Surv(StartTime, StopTime, Fail) ~ SEXcat+ zSES_score + zAge + Premature_0yesCAT + EarInfect_NoPECAT + PEtubesCAT + zspokefirstword + READ_1yesCAT, data=data_narrative)

summary(Model_Narrative)

coxph(formula = Surv(StartTime, StopTime, Fail) ~ SEXcat + zSES_score +

zAge + Premature_0yesCAT + EarInfect_NoPECAT + PEtubesCAT +

zspokefirstword + READ_1yesCAT, data = data_narrative)

n= 8137, number of events= 1937

(2365 observations deleted due to missingness)

coef exp(coef) se(coef) z Pr(>|z|)

SEXcatMALE -0.004590 0.995421 0.046582 -0.099 0.9215

zSES_score -0.288100 0.749686 0.020767 -13.873 < 2e-16 ***

zAge -0.007612 0.992417 0.074701 -0.102 0.9188

Premature_0yesCAT1 -0.082013 0.921260 0.059030 -1.389 0.1647

EarInfect_NoPECAT1 -0.028383 0.972016 0.100587 -0.282 0.7778

PEtubesCAT1 -0.191845 0.825435 0.086750 -2.211 0.0270 *

**zspokefirstword 0.154866 1.167501 0.022876 6.770 1.29e-11 *****

READ_1yesCAT1 -0.097757 0.906869 0.047731 -2.048 0.0406 *

---

Signif. codes: 0 ‘***’ 0.001 ‘**’ 0.01 ‘*’ 0.05 ‘.’ 0.1 ‘ ’ 1

exp(coef) exp(-coef) lower .95 upper .95

SEXcatMALE 0.9954 1.0046 0.9086 1.0906

zSES_score 0.7497 1.3339 0.7198 0.7808

zAge 0.9924 1.0076 0.8573 1.1489

Premature_0yesCAT1 0.9213 1.0855 0.8206 1.0343

EarInfect_NoPECAT1 0.9720 1.0288 0.7981 1.1838

PEtubesCAT1 0.8254 1.2115 0.6964 0.9784

**zspokefirstword 1.1675 0.8565 1.1163 1.2210**

READ_1yesCAT1 0.9069 1.1027 0.8259 0.9958

Concordance= 0.62 (se = 0.008 )

Rsquare= 0.03 (max possible= 0.972 )

Likelihood ratio test= 249 on 8 df, p=<2e-16

Wald test = 267.4 on 8 df, p=<2e-16

Score (logrank) test = 270.7 on 8 df, p=<2e-16

#Survival Analyses (Narrative) predicted by put several words together

Model_Narrative <- coxph(Surv(StartTime, StopTime, Fail) ~ SEXcat+ zSES_score + zAge + Premature_0yesCAT + EarInfect_NoPECAT + PEtubesCAT + zputseveralwordstogether + READ_1yesCAT, data=data_narrative)

summary(Model_Narrative)

coxph(formula = Surv(StartTime, StopTime, Fail) ~ SEXcat + zSES_score +

zAge + Premature_0yesCAT + EarInfect_NoPECAT + PEtubesCAT +

zputseveralwordstogether + READ_1yesCAT, data = data_narrative)

n= 8185, number of events= 1956

(2317 observations deleted due to missingness)

coef exp(coef) se(coef) z Pr(>|z|)

SEXcatMALE 0.03813 1.03887 0.04639 0.822 0.4111

zSES_score -0.27598 0.75883 0.02072 -13.322 <2e-16 ***

zAge -0.04571 0.95532 0.07335 -0.623 0.5332

Premature_0yesCAT1 -0.07032 0.93210 0.05823 -1.208 0.2272

EarInfect_NoPECAT1 -0.15634 0.85527 0.10311 -1.516 0.1295

PEtubesCAT1 -0.21099 0.80978 0.08642 -2.441 0.0146 *

**zputseveralwordstogether 0.23952 1.27064 0.02223 10.775 <2e-16 *****

READ_1yesCAT1 -0.09658 0.90794 0.04745 -2.035 0.0418 *

---

Signif. codes: 0 ‘***’ 0.001 ‘**’ 0.01 ‘*’ 0.05 ‘.’ 0.1 ‘ ’ 1

exp(coef) exp(-coef) lower .95 upper .95

SEXcatMALE 1.0389 0.9626 0.9486 1.1377

zSES_score 0.7588 1.3178 0.7286 0.7903

zAge 0.9553 1.0468 0.8274 1.1030

Premature_0yesCAT1 0.9321 1.0728 0.8316 1.0448

EarInfect_NoPECAT1 0.8553 1.1692 0.6988 1.0468

PEtubesCAT1 0.8098 1.2349 0.6836 0.9592

**zputseveralwordstogether 1.2706 0.7870 1.2165 1.3272**

READ_1yesCAT1 0.9079 1.1014 0.8273 0.9964

Concordance= 0.62 (se = 0.008 )

Rsquare= 0.038 (max possible= 0.972 )

Likelihood ratio test= 318.4 on 8 df, p=<2e-16

Wald test = 339 on 8 df, p=<2e-16

Score (logrank) test = 343.3 on 8 df, p=<2e-16
